# Synthesis, In Vitro Screening and Docking Studies of New Thiosemicarbazide Derivatives as Antitubercular Agents

**DOI:** 10.3390/molecules24020251

**Published:** 2019-01-11

**Authors:** Monika Pitucha, Zbigniew Karczmarzyk, Marta Swatko-Ossor, Waldemar Wysocki, Maciej Wos, Kamil Chudzik, Grazyna Ginalska, Andrzej Fruzinski

**Affiliations:** 1 Independent Radiopharmacy Unit, Faculty of Pharmacy with Division of Medical Analytics, Medical University of Lublin, 20-093 Lublin, Poland; wos.maciej@gmail.com (M.W.); kamilchudzik@umlub.pl (K.C.); 2 Faculty of Science, Siedlce University of Natural Sciences and Humanities, 08-110 Siedlce, Poland; kar@uph.edu.pl (Z.K.); wwysocki@uph.edu.pl (W.W.); 3 Department of Biochemistry and Biotechnology, Faculty of Pharmacy with Division of Medical Analytics, Medical University of Lublin, 20-093 Lublin, Poland; martaswatkoossor@umlub.pl (M.S.-O.); grazyna.ginalska@umlub.pl (G.G.); 4 Institute of General and Ecological Chemistry, Technical University, 90-924 Łódź, Poland; andrzej.fruzinski@p.lodz.pl

**Keywords:** synthesis, thiosemicarbazide, X-ray analysis, tuberculosis, molecular docking

## Abstract

A series of thiosemicarbazide derivatives was designed and synthesized by reaction of carboxylic acid hydrazide with isothiocyanates. The molecular structures of the investigated thiosemicarbazides were confirmed and characterized by spectroscopic analysis. The conformational preference of carbonylthiosemicarbazide chain and intra- and intermolecular interactions in the crystalline state were characterized using X-ray analysis. The antituberculosis activity of the target compounds were tested in vitro against four *Mycobacterium* strains: M. H37Ra, *M. phlei*, *M. smegmatis*, *M. timereck*. The most active compounds were those with 2-pyridine ring. They exhibited lower minimal inhibitory concentration (MIC) values in the range 7.81–31.25 μg/mL in comparison to the other isomers. Compound **5** had activity against *M. smegmatis* at a concentration of 7.81 μg/mL whereas compound **2** had activity against all tested strains at a concentration of 15.625 μg/mL. The molecular docking studies were performed for investigated compounds using the *Mycobacterium tuberculosis* glutamine synthetase MtGS as their molecular target.

## 1. Introduction

Tuberculosis is the one of the most common infectious diseases worldwide [1]. Next to AIDS (Acquired Immune Deficiency Syndrome), it is the leading cause of death in the world. Despite the availability of tuberculosis treatment, about 9 million new cases of tuberculosis are reported each year, and 1.5 million cases are fatal. The worst epidemiological situation is in Third World countries, where the incidence rates range from 100–300 cases per 100,000 inhabitants [2]. More than 95% of cases fall to the developing countries of Asia, Africa, Brazil and Russia. The results of treatment are still not satisfactory. The success rate for new cases and surgeries reported in 2013 was 76% [3]. Despite intensive efforts to prevent and treat tuberculosis, the problem is serious. MDR-TB (multi-drug-resistant tuberculosis) is a disturbing phenomenon which embraces strains resistant to two basic drugs: Isoniazid and Rifampicin. Moreover, mycobacterial resistance to Rifampicin, Isoniazid, quinolones and aminoglycosides has increased—extensively drug-resistant tuberculosis (XD-TB) [4]. The heterogeneous populations of mycobacteria (active and dormant) and ease of acquisition of resistance requires a combination therapy with antibiotics of different molecular mechanisms of action [5]. This makes the treatment of multidrug-resistant tuberculosis long and costly, and it often fails. Another problem is the interaction between antiviral drugs used in patients infected with the HIV virus and anti-TB drugs [6]. Therefore, interests and efforts of many research groups around the world are focused on tuberculosis problems. Actions taken to reduce the effects of *M. tuberculosis* resistance are multidirectional. Currently, an intensive search for new, effective anti-tuberculosis drugs is under way. It is a long-lasting and costly process, so it is important to understand the mechanism of action of proven and effective antituberculosis drugs. The development of counterparts with better pharmacokinetic properties and less toxicity appears to be one of the methods of searching for new potential drugs.

An excellent example of this type of solution seems to be the modification of Isoniazid, a commonly used antituberculosis drug [7]. Isoniazid is a bactericidal germicide that is isonicotinic acid hydrazide. It is used in the treatment and prevention of tuberculosis, in combination with other antituberculosis agents.

In our work we propose the synthesis of Isoniazide analogs as compounds with potential antitubercular activity. By using duality of biological action, we have designed compounds having a pyridine ring and a thiosemicarbazide system as a well-known carrier antituberculosis agent [8,9].

## 2. Experimental

### 2.1. Synthesis

All chemicals used for the synthesis were purchased from Sigma-Aldrich, AlfaAesar and POCH (Polish Chemical Reagents, Gliwice, Poland) companies and used without further purification. Melting points were determined using Fisher–Johns block and presented without corrections. The ^1^H and ^l3^C NMR spectra were recorded on a Bruker Avance 600 spectrometer (Bruker BioSpin GmbH, Rheinstetten, Germany) in dimethyl sulfoxide (DMSO-d_6_). The chemical shifts are given in δ (ppm) scale using tetramethylsilane (TMS) as the standard reference. The attenuated total reflectance ATR-IR spectra were recorded over the range 4000–400 cm⁻^1^ on the Thermo Scientific Nicolet 6700 FTIR spectrophotometer equipped with ATR attachment with diamond crystal and Omnic software version 8.2. For accurate mass measurements an Agilent Technologies liquid chromatograph 1290 coupled to an Agilent Technologies 6550 iFunnel Q-TOF LC/MS equipped with Jet Stream Technology Ion Source was employed. Flow injection analyses were performed using 0.1% formic acid in water:acetonitrile (50:50 *v*/*v*), mobile phase flow 0.5 mL min^−1^. Ions were acquired in positive polarity. Mass correction was enabled, ions of *m*/*z* 121.0509 and 922.0098 were used as the reference ions. Instrument control, data acquisition and analysis were performed using Agilent MassHunter Workstation B.07 Software. Single-charged protonated ions for all examined compounds were detected.

The homogeneity of the newly obtained compounds was confirmed by TLC method on aluminum oxide 60 F_254_ plates (Merck) in a CHCl_3_/C_2_H_5_OH (10:1 and 10:2) solvent system with UV visualization. Compounds **1**–**4**, **7**, **8**, **11**, **13**, **15**, **16**, **18**, **20**, **21** have been synthesized in our laboratory and reported in literature [10,11,12]. Compounds **5**, **6**, **12**, **14**, and **19** have been published previously [13,14,15,16,17].

#### 2.1.1. General Procedure for the Synthesis of 1-(pyridin-2-,3-,4-yl)carbonyl-4-substituted Thiosemicarbazide (1–19)

The pyridine 2-, 3- or 4-pyridinecarboxylic acid hydrazide (0.01 mol) was dissolved in 15 mL methanol and 0.01 mol of appropriate isothiocyanate was added. Mixtures were heated for 0.5–1 h at reflux temperature. After this time mixture was cooled and appropriate thiosemicarbazide was crystallized from methanol. (Supplementary Material includes experimental data of all compounds).

#### 2.1.2. General Procedure for the Synthesis of 1-(pyridin-4-ylacetyl)-4-substituted Thiosemicarbazide (20–25)

A mixture of 4-pyridine acetic acid hydrazide (0.01 mol) and appropriate isothiocyanate (0.01 mol) in anhydrous diethyl ether (10 mL) was kept at room temperature for 24 h. The product was filtered off and crystallized from ethanol. (Supplementary Material includes experimental data of all compounds).

### 2.2. X-ray Structure Determination

X-ray data of **4**, **7**, **11**, **13** and **14** were collected on the Rigaku Oxford XtaLAB Synergy, Pilatus 300K diffractometer; crystal sizes 0.45 × 0.20 × 0.13 mm for **4**, 0.59 × 0.07 × 0.07 mm for **7**, 0.59 × 0.28 × 0.14 mm for **11**, 0.58 × 0.27 × 0.06 mm for **13** and 0.50 × 0.16 × 0.03 mm for **14**; CuK*α* (*λ* = 1.54178 Å) radiation, *ω* scans, *T* = 293 K, absorption correction: multi-scan [18], *T*_min_/*T*_max_ of 0.344/1.000 for **4**, 0.419/1.000 for **7**, 0.282/1.000 for **11**, 0.349/1.000 for **13** and 0.759/1.000 for **14**. The structures were solved by direct methods using SHELXS97 [19] and refined by full-matrix least-squares with SHELXL97 [19]. The N-bound H atoms were located by difference Fourier synthesis and refined freely. The remaining H atoms were positioned geometrically and treated as riding on their parent C atoms with C–H distances of 0.93 Å (aromatic), 0.98 Å (CH), 0.97 Å (CH_2_) and 0.96 Å (CH_3_) and O–H distance of 0.82 Å. All H atoms were refined with isotropic displacement parameters taken as 1.5 times those of the respective parent atoms. The compound **7** crystallizes in non-centrosymmetric space group *Cc*. The absolute structure of crystal was confirmed by Flack parameter of 0.00(2) with 1116 Friedel pairs [20]. The residual electron density map for **14** reveals the molecule of dimethyl-diazene-*N*,*N*’-dioxide lying on 2-fold axis passing through the center of the central N=N bond. In this molecule the N–C and N–O bond lengths were fixed to 1.507(9) and 1.430(9) Å, respectively, due to the unstable geometry of this molecule in the refinement procedure. Moreover, the final residual electron-density maps showed that the C14 and C15 atoms of the benzene ring are disordered over two sites designed as A and B in the refinement procedure. The occupancy factors (sof) for the split carbon atoms were refined and finally fixed at 0.5. Additionally, the geometry (bond lengths and angles) of disordered fragments of benzene ring was corrected using appropriate DFIX instructions of SHELXL97.

All calculations were performed using WINGX version 1.64.05 package [21]. CCDC-1864683 for **4**, 1,864,679 for **7**, 1,864,681 for **11**, 1,864,682 for **13** and 1,864,680 for **14** contain the supplementary crystallographic data for this paper. These data can be obtained free of charge at www.ccdc.cam.ac.uk/conts/retrieving.html [or from the Cambridge Crystallographic Data Centre (CCDC), 12 Union Road, Cambridge CB2 1EZ, UK; fax: +44(0) 1223 336 033; email: deposit@ccdc.cam.ac.uk]. (Supplementary Material includes experimental data of compounds **4**, **7**, **11**, **13** and **14** in CIF files).

*Crystal data of **4***: C_13_H_10_N_4_OSCl_2_,*M* = 341.21, triclinic, space group *P*$\bar{1}$, *a* = 7.4539(2), *b* = 7.8084(2), *c* = 14.3223(2) Å, *α* = 104.442(2), *β* = 92.188(2), *γ* = 112.898(2)^°^, *V* = 735.05(3) Å^3^, *Z* = 2, *d*_calc_ = 1.542 Mg m^−3^, *F*(000) = 348, *μ*(Cu K*α*) = 5.339 mm^−1^, *T* = 293 K, 14,710 measured reflections (*θ* range 6.31–78.65^°^), 3041 unique reflections, final *R* = 0.034, *wR* = 0.092, *S* = 1.068 for 2964 reflections with *I* > 2*σ*(*I*).

*Crystal data of **7***: C_14_H_14_N_4_OS_2_,*M* = 318.41, monoclinic, space group *Cc*, *a* = 7.9236(4), *b* = 25.2269(2), *c* = 7.9636(2) Å, *β* = 113.018(2)^o^, *V* = 1465.09(9) Å^3^, *Z* = 4, *d*_calc_ = 1.444 Mg m^−3^, *F*(000) = 664, *μ*(Cu K*α*) = 3.332 mm^−1^, *T* = 293K, 7385 measured reflections (*θ* range 6.32–78.94^°^), 2622 unique reflections, final *R* = 0.039, *wR*= 0.107, *S* = 1.076 for 2597 reflections with *I* > 2*σ*(*I*).

*Crystal data of **11***: C_13_H_10_N_4_OSCl_2_,*M* = 341.21, monoclinic, space group *P*2_1_/*c*, *a* = 4.5176(1), *b* = 27.8468(4), *c* = 11.8424(1) Å, *β* = 98.573(1)^°^, *V* = 1473.14(4) Å^3^, *Z* = 4, *d*_calc_ = 1.538 Mg m^−3^, *F*(000) = 696, *μ*(Cu K*α*) = 5.328 mm^−1^, *T* = 293K, 15,369 measured reflections (*θ* range 4.09–78.76^°^), 3028 unique reflections, final *R* = 0.040, *wR* = 0.111, *S* = 1.044 for 2761 reflections with *I* > 2*σ*(*I*).

*Crystal data of **13***: 2(C_13_H_11_N_4_OSF)· C_3_H_8_O,*M* = 640.72, monoclinic, space group *P*2_1_/*n*, *a* = 13.4658(2), *b* = 9.8028(1), *c* = 23.9666(3) Å, *β* = 104.402(1)^°^, *V* = 3064.23(7) Å^3^, *Z* = 4, *d*_calc_ = 1.389 Mg m^−3^, *F*(000) = 1336, *μ*(Cu K*α*) = 2.070 mm^−1^, *T* = 293 K, 32,030 measured reflections (*θ* range 3.81–78.86^°^), 6328 unique reflections, final *R* = 0.057, *wR* = 0.166, *S* = 1.062 for 5725 reflections with *I* > 2*σ*(*I*).

*Crystal data of **14***: C_13_H_11_N_4_OSCl· 0.5·C_2_H_6_N_2_O_2_,*M* = 351.81, monoclinic, space group *C*2/*c*, *a* = 24.0692(5), *b* = 9.7448(2), *c* = 13.4379(3) Å, *β* = 103.383(2)^°^, *V* = 3066.26(12) Å^3^, *Z* = 8, *d*_calc_ = 1.524 Mg m^−3^, *F*(000) = 1456, *μ*(Cu K*α*) = 3.640 mm^−1^, *T* = 293 K, 8718 measured reflections (*θ* range 3.77–78.55^°^), 3143 unique reflections, final *R* = 0.066, *wR* = 0.189, *S* = 1.125 for 2901 reflections with *I* > 2*σ*(*I*).

### 2.3. Antimycobacterial Assay

Four strains of mycobacteria, *M. tuberculosis H37Ra, M. phlei, M. smegmatis* and *M. timereck,* were used for the study. The strains were donated by National Tuberculosis and Lung Diseases Research Institute (Warsaw, Poland). The microorganisms were stored on Lewenstein–Jensen media at 4 °C, then transferred to vials containing Middlebrook 7H9 broth supplemented with sodium chloride, bovine albumin, dextrose and catalase (ADC-BBL/Becton-Dickinson, USA) and grown at 37 °C. The MIC values (minimal inhibitory concentration) for the tested compounds were determined by log2 dilution method (Turnidge and Jorgensen, 1999).

The stock solutions of the tested compounds were freshly prepared in dimethyl sulfoxide (DMSO). The concentrations of test substances were in the range of 500–1.95 µg/mL.

Inoculum for susceptibility testing was prepared by scraping microorganisms stored on Lewnstein’s slants followed by their transfer to the vial containing Middlebrook 7H9 broth with ADC enrichment. The bacterial suspension was then disrupted by vortexing with glass beads for 1 min. The supernatant was collected and equalized to 1.0 McFarland with fresh medium. Turbidity of the suspensions was measured using nephelometer (Becton, Dickinson and Company, Franklin Lake, MD, USA). Then, 12 μL of inoculum was added to each tube containing the tested compounds and incubated at 37 °C. The observations were carried out for 21 days for *M. 37Ra* and 7 days for other strains. Rifampicin was used as a control.

The growth of mycobacteria was assessed using a visual method. The MIC value was determined as the lowest concentration of the drug at which no growth of microorganisms was observed (no turbidity).

A sample containing the same volume of an inoculum (but cultured in the absence of drug) served as a positive control, the medium alone (without the bacteria) was a negative control. Controls were incubated in the same condition as a test tube. In addition, a control containing DMSO at the highest concentration used in the experiment (1.25%) was prepared to exclude the bactericidal action of the solvent itself.

The zones of growth inhibition were determined by a diffusion well test on Middlebrook 7H10 medium containing the enrichment of OADC (oleic acid, bovine albumin fraction, dextrose and catalase (Becton, Dickinson and Company, Franklin Lake, MD, USA). Petri dishes (φ 10 cm) were filled with 25 mL of sterilized medium and allowed to solidify. Then, 200 microliters of bacterial suspension with a density corresponding to 0.5 McFarland was spread over the surface and wells with a diameter of 4 mm were cut out (3 per plate). Afterwards, 50 microliters of the tested drugs were introduced into the wells. The tested compounds were dissolved in DMSO. The concentration of the stock solution was 5 mg/mL. Rifampicin was used as a control.

The plates were incubated at 37 °C for 21 days for M. H37Ra and 7 days for the remaining strains. Zones of inhibition were evaluated using colony counter (SCAN^®^ 500, Interscience, St Nom la Breteche, France).

### 2.4. Computational Details

The crystal structure of *Mycobacterium tuberculosis* glutamine synthetase MtGS in complex with magnesium, adenosine-5’-diphosphate (ADP) and L-methionine-s-sulfoximine phosphate MSO-P was downloaded from Protein Data Bank (PDB ID: 2BVC [22]). Docking studies were performed using the GOLD Suite v.5.5 software [23]. Preparation of enzyme (addition of hydrogens, removal of water molecules, extraction of original ligands from the protein active site,) were done with GOLD as per default settings. Binding site was determined using the previous knowledge of the original ligand’s interaction site. In docking stimulations, each ligand was kept flexible but the amino acid residues of the enzyme were held rigid. For the simulation runs default parameter values were taken. The selection of atoms in the active site within 6 Å of original ligands was chosen as default. ChemPLP was selected as the scoring function to rank the compounds to be investigated. Protein–ligand interactions were analyzed using Hermes v1.8.2 [23]. The molecular structures of ligands were fully optimized without any constraint at the DFT/B3LYP [24,25] level with 6–311^++^G(d,p.) basis set using GAUSSIAN 09 [26]. The conformational preferences of **4** were calculated using semiempirical AM1 method implemented in GAUSSIAN 09.

## 3. Results and Discussion

### 3.1. Chemistry

**Scheme 1:**
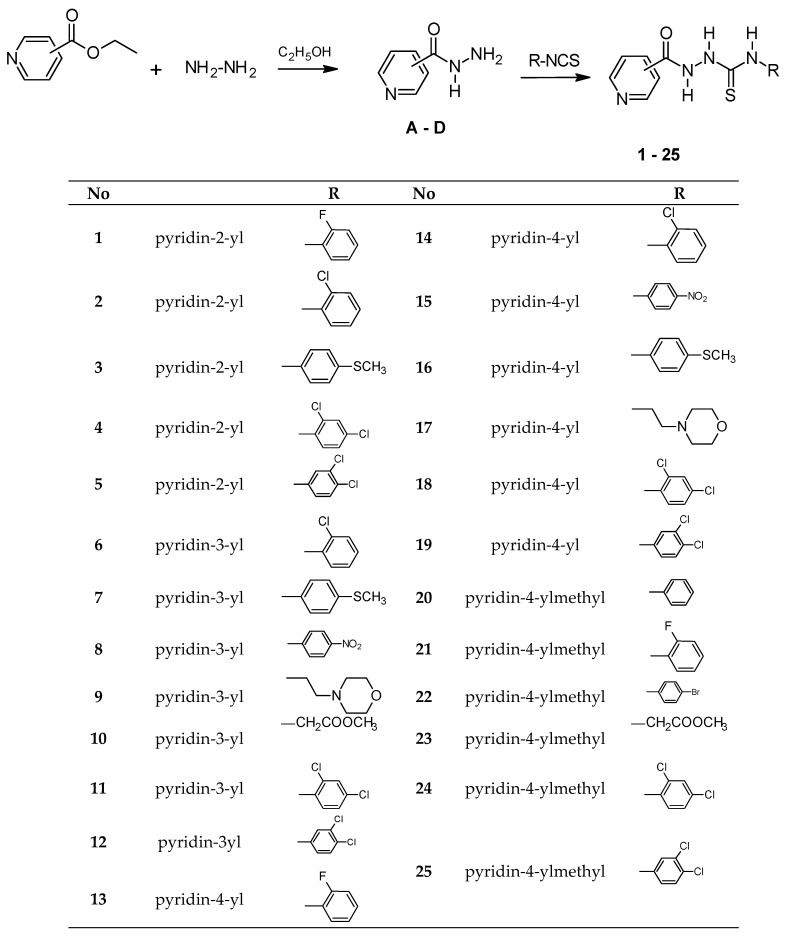
Synthesis of title compounds.

The substrates for the planned synthesis were hydrazides of carboxylic acids: 2-pyridinecarboxylic acid hydrazide (**A**), 3-pyridinecarboxylic acid hydrazide (**B**), 4-pyridinecarboxylic acid hydrazide (**C**) and 4-pyridineacetic acid hydrazide (**D**). Hydrazide C is available commercially. Other hydrazides were prepared by the method described in the literature [10,12]. The title compounds were obtained by reaction of the corresponding hydrazide with commercially available isothiocyanates: 2-fluorophenyl, 2-chlorophenyl, 4-methyltiophenyl, 2,4-dichlorophenyl, phenyl, 3,4-dichlorophenyl, 4-nitrophenyl, 4-bromophenyl, morpholinoethyl, and methoxycarbonylomethyl isothiocyanate. The reaction was carried out at the boiling point of methanol for 0.5–1 h (Scheme 1). Although the methods of synthesis of thiosemicarbazide derivatives are known and described in the literature [27,28,29] they require continuous modifications depending on the substrates used. Two methods for the preparation of thiosemicarbazide derivatives are commonly used: in solution and at the melting point. The reaction carried out at high temperature melts the substrates and yields the reaction product in good yield. The disadvantage of this method is high temperature (about 180 °C), which can cause charring of products or cyclization of intermediates of thiosemicarbazide. The process is carried out by heating for several hours (about 20 h), prolonging the synthesis time. In addition, this method causes the substance to be baked and the difficulty in its isolation, especially with poor solubility of the product. In turn, the preparation of thiosemicarbazide derivatives in the solution requires the selection of a suitable solvent. Using *N*,*N*-dimethylacetamide or acetonitrile the reaction can be carried out at room temperature or by heating the starting materials. The desired product is usually isolated by distilling off the solvent or adding water (precipitation). Unfortunately, the separation of the product is often difficult, which limits the use of this method. Too high heating temperature can direct ring closure. The cyclization problem can be solved by using diethyl ether (lower boiling solvent) as the solvent. The disadvantage of this method is the poor solubility of substrates, which causes the reaction to occur at low yield, and the product obtained requires multiple crystallization in order to get rid of unreacted substrates (mainly hydrazide). In this research work it was decided to use methanol as solvate and to heat substrates at boiling point. Proposed method of obtaining thiosemicarbazide derivatives is a modification of the procedures described in the literature. Its advantage is the short heating time (0.5–1 h) and the ease of isolation of the product from the mixture by filtration or partial evaporation of the solvent (methanol). The solubility difference between hydrazide and thiosemicarbazide in methanol is not insignificant. The applied method is characterized by satisfactory efficiency, and the obtained product often does not require crystallization. In addition, the reaction environment excludes the possibility of cyclization which guarantees obtaining homogeneous compounds. As a result of the syntheses carried out, 25 derivatives of thiosemicarbazide contained a pyridine ring were obtained. The reaction and the purity of the compounds obtained were determined by thin layer chromatography (TLC) using ready-made Merck chromatographic plates (aluminum oxide 60 F-254).As mobile phases a mixture of chloroform and ethanol in a ratio of 10:1 or 8:2 was used. Chromatograms were visualized in UV light with a wavelength of 254 nm. The structure of the obtained compounds was confirmed by spectral analysis, mass spectrometry and crystallography analysis. Experimental data of all compounds are described in Supplementary Material.

### 3.2. X-Ray Analysis

The X-ray analyses for **4**, **7**, **11**, **13** and **14** as the model compounds were performed in order to confirm the synthesis pathway, assumed molecular structures, and identification of the tautomeric form, and to find their conformational preferences observed in crystalline state.

**Figure 1:**
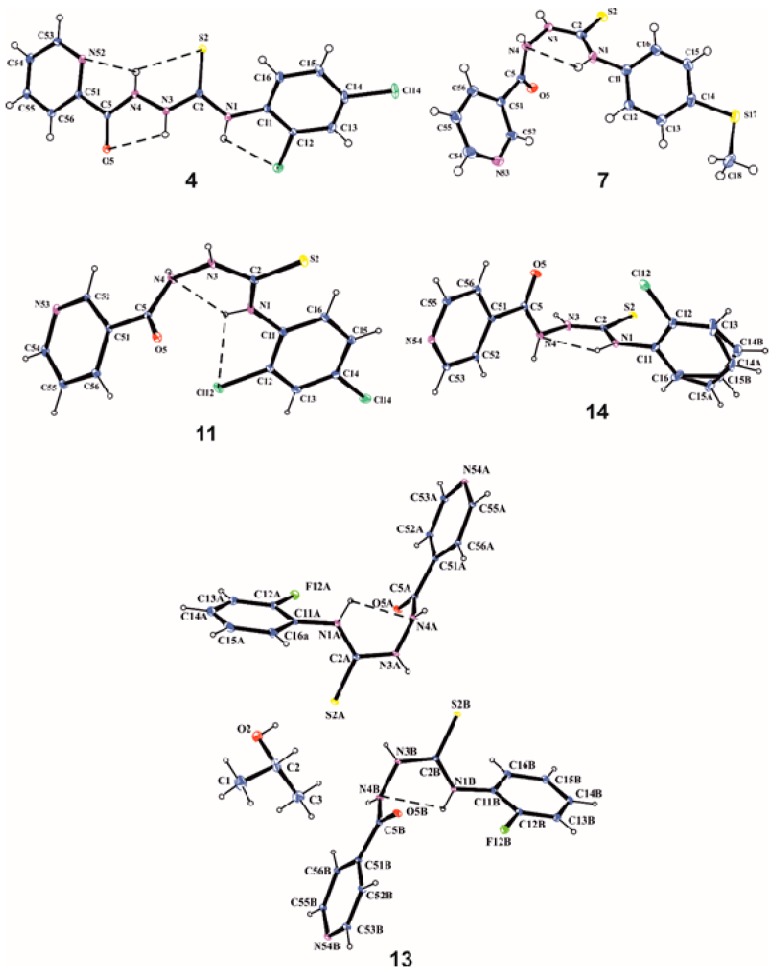
The molecular structures of compounds **4**, **7**, **11**, **13** and **14** with atom labelling and displacement ellipsoids drawing at the 30% probability level.

The compound **13** crystallizes with one 2-propanol solvent molecule were attributable to two molecules of the base. The structures and conformations of the molecules **4**, **7**, **11**, **13** and **14** in the crystal are shown in Figure 1. In the crystalline state, all investigated molecules exist in N1-amino/N3-amino/N4-amino/S2-thione/O5-keto tautomeric form, which is confirmed by the C2–S2 and C5–O5 bond length of 1.6730(1) and 1.238(2) Å in **4**, 1.695(4) and 1.226(4) Å in **7**, 1.6733(17) and 1.217(2) Å in **11**, 1.695(2) and 1.226(3) Å in molecule A and 1.690(2) and 1.226(3) Å in molecule B of **13** and 1.728(4) and 1.220(3) Å in **14**, respectively, typical for the carbonyl and thione group in amides (1.234(12) Å) and thioureas (1.681(20) Å), respectively [30]. Moreover, the positions of the amino-H atoms are localized at the difference electron-density maps in the vicinity of N1, N3 and N4 atoms for all investigated crystals.

The conformation of carbonylthiosemicarbazide spacer depends on the nature of substituents at the ends of this chain. The torsion angles C11–N1–C2–N3, N1–C2–N3–N4, C2–N3–N4–C5 and N3–N4–C5–C51 with values presented in Table 1 show that the carbonylthiosemicarbazide chain adopts *trans*-*cis*-*gauche*-*trans* conformation in **7**, **11**, **13** and **14** with 3- and 4-pyridine substituent and *all trans* conformation in **4** with 2-pyridine group. This difference in conformation causes that the thione C2–S2 group has *trans* and *cis* conformation with respect to N3–N4 (torsion angle C11–N1–C2–S2) and N1–C11 (torsion angle N4–N3–C2–S2) bonds in **7**, **11**, **13** and **14**, while the *cis* and *cis* conformation is observed in **4** (Table 1). Consequently, the torsion angles N52–C51–C5–O5 in **4** and O5–C5–C51–C52(C56) in **13** and **14** show that the carbonyl group is practically coplanar with the pyridine ring, while the torsion angles O5–C5–C51–C52(C56) indicate that this group is slightly distorted from coplanarity. The analysis of the differences in conformation of investigated molecules shows two possible conformations *cis* and *trans* on C2–N3 bond and conformation *trans* and *gauche* on N3–N4 bond. The energy effect of the free-rotation at C2–N3 and N3–N4 bonds for **4** was calculated using AM1 semiempirical method. The energies of conformations were minimized and all geometrical parameters optimized for each rotation with a 10° increment from −180° to 180° of *φ*_1_ = N1–C2–N3–N4 and *φ*_2_ = C2–N3–N4–C5 torsion angles as shown in Figure S1 and Figure S2, respectively (Supplementary Material). One can see for *φ*_1_, that the minima of energy *cis* (*φ*_1_ = −20°) and *trans* (*φ*_1_ = 160°) are separated by the energy barriers estimated about 6.9 kcal/mol, and within range of *φ*_1_ from −20 to 180° the energy changes do not exceed 2.8 kcal/mol. In the case of *φ*_2_, two minima of energy (*gauche*; *φ*_2_ = 100° and *φ*_2_ = −90°) are separated by the energy barriers not exceeding 9.5 kcal/mol. It should be noted that calculated energy barriers do not prevent molecules against conformational changes in physiological conditions.

The tautomeric form and the conformations of the investigated molecules are stabilized by the intramolecular hydrogen bonds: N1–H1…N4 in **7**, **11**, **13** and **14**, N1–H1…Cl12 in **4** and **11** and N3–H3...O5, N4–H4...S2 and N4–H4...N52 in **4** (Figure 1, Table 2).

In the crystal structures of **4**, **7**, **11**, **13** and **14** the packing of the molecules is determined by a net of intermolecular hydrogen bonds N–H…X (X = O, S, N), as a consequence of the tautomeric form observed in crystals, where amino N–H groups are the proton donors and O-carbonyl, S-thione and N-pyridine atoms interact as proton acceptors (Table 2). Moreover, the overlapping of parallel benzene rings in **4** and pyridine rings in **13** and **14** is observed with the π…π distances characteristic for π-stacking. A detailed description of the crystal structures of **4**, **7**, **11**, **13** and **14** with the structural motifs formed by molecules interacting via intermolecular hydrogen bonds is presented in Supplementary Material. The analysis of the intermolecular interactions in the crystal may be helpful in prediction of the possible interactions of the molecules of investigated compounds with the binding site of potential molecular target related to biological activity.

### 3.3. Antimycobacterial Activity

All obtained (**1**–**25**) compounds were tested in vitro against four *Mycobacterium* strains: *M. H_37_Ra*, *M. phlei*, *M. smegmatis, M. timereck*. The tests were performed by disc diffusion and broth microdilution. The obtained results indicate the biological potential and varying activity depending on the type of substituent in the position of the thiosemicarbazide and the pyridine ring substitution site. Of the substances tested, the most promising seems to be compound **4** for which the growth inhibition zones were respectively 29.6 mm (*M. H_37_Ra*), 30.1 mm (*M. phlei*), 31.0 mm (*M. smegmatis*), and 29.8 mm *(M. timereck*). For the remaining compounds, the growth inhibition zone ranged from 0 to 30.5 mm (*M. H_37_Ra*), 0 to 30.1 mm (*M. phlei*, *M. smegmatis*) and 0 to 29.8 mm (*M. timereck*). Analysis of the activity within the strains showed that compound **3** effectively inhibited the growth of *M. H_37_Ra* (30.5 mm), compound **4** inhibited the growth of *M. phlei* and *M. smegmatis* (30.0 mm) and *M. timereck* (29.8 mm). Detailed data are given in Table 3.

**Figure 2:**
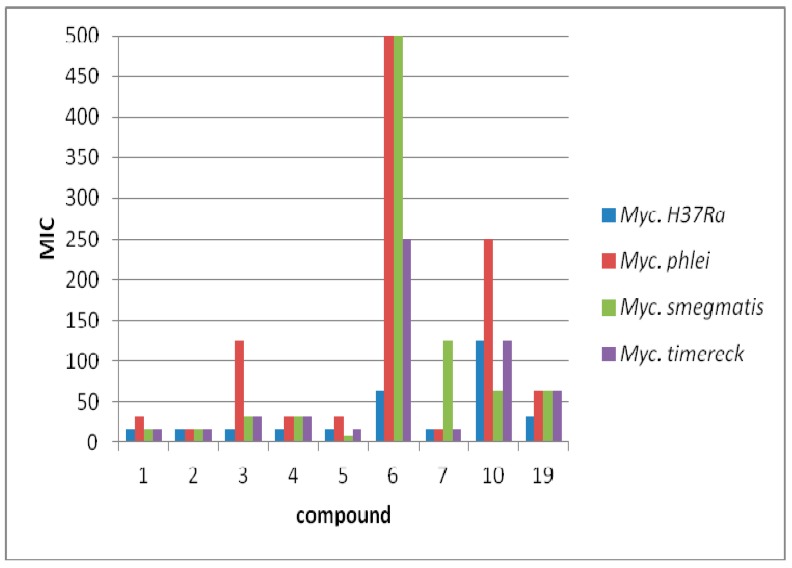
Antimycobacterial activity of active compounds.

**Figure 3:**
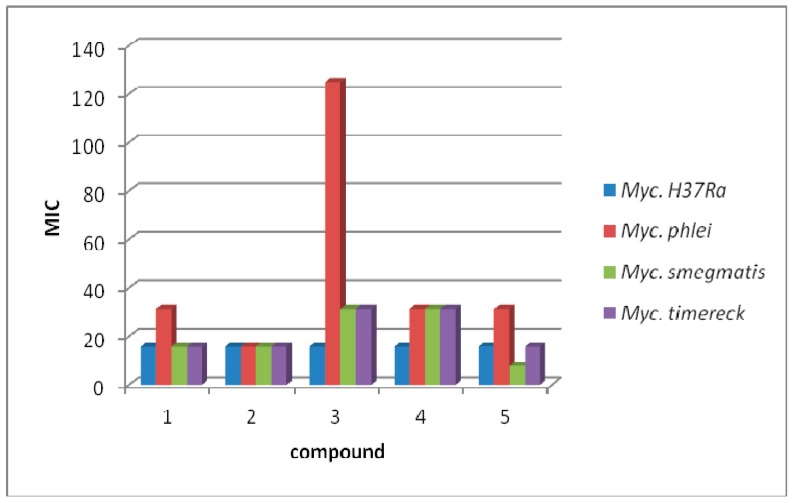
Antimycobacterial activity of the most active compounds.

The determined minimal inhibitory concentration with 2-pyridine ring exhibited lower MIC values (7.81–31.25 μg/mL) in comparison to the other isomers (Figure 2). The exception is compound **3**, which caused the inhibition of growth of *M. phlei* at a concentration of 125 μg/mL. The lowest MIC value (7.81 μg/mL) was obtained for compound **5** against *M. smegmatis*. For the remaining strains, these values ranged within 15.625 and 31.25 μg/mL. Compound 2 (having a 2-chlorophenyl substituent) had activity against all tested strains at a concentration of 15.625 μg/mL. Replacing chlorine with fluorine (**1**) results in an increase in the minimum inhibitory concentration twice against *M. pheli*. In turn, the additional chlorine atom (**4**) causes an increase in the inhibitory concentration against *M. phlei*, *M. smegmatis*, *M. timereck*. Less promising were the compounds associated with the pyridine ring in the para position, with MIC values above 250 μg/mL. Only compound **19** showed inhibition of growth of microorganisms at concentrations of 31.25–62.5 μg/mL (Figure 3). No antituberculosis activity was observed for compounds linked to the pyridine ring with a methylene linker (**20–25**). MIC values above 250 μg/mL were observed for these derivatives. Detailed data are shown in Table 4.

### 3.4. Molecular Docking

**Figure 4:**
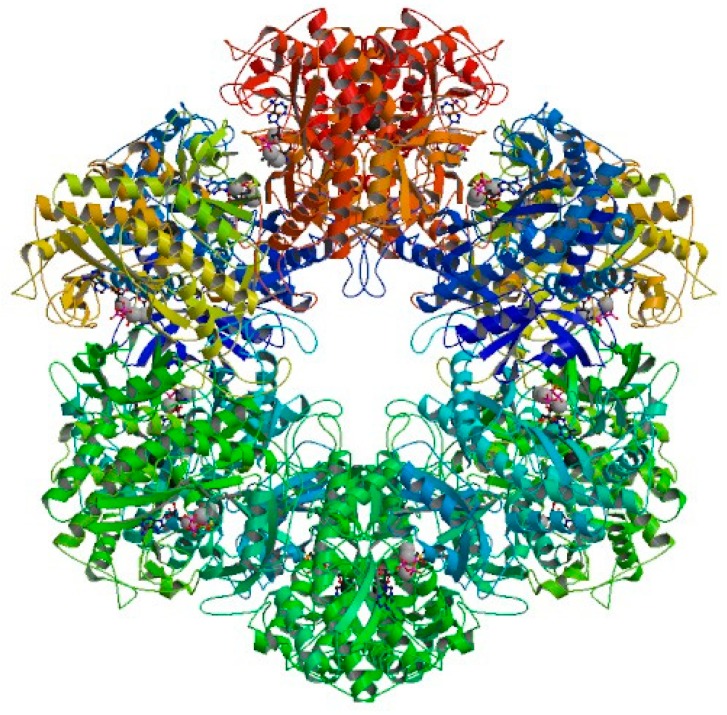
Overall MtGS structure. The active sites represented by a ball-and-stick model of MSO-P and ADP are located at subunit-subunit interfaces.

The thiosemicarbazide derivatives containing pyridine ring **1**–**25** were tested in virtual screening to the *Mycobacterium tuberculosis* glutamine synthetase MtGS. Glutamine synthetase as an enzyme is a central component of bacterial nitrogen metabolism [25]. It catalyzes the ligation of glutamate and ammonia forming L-glutamine, ADP, phosphate and a proton with the hydrolysis of ATP being a potential drug target [25,31,32,33]. An enzyme MtGS is of particular interest as a new molecular target in a novel antibiotic strategy, due to its relationship to pathogenicity of mycobacteria [33]. Therefore, we attempted to check in silico the affinity of the investigated thiosemicarbazide derivatives for the enzyme MtGS as its potential inhibitors. In crystalline state, MtGS occurs in a complex with magnesium, adenosine-5’-diphosphate ADP and L-methionine-s-sulfoximine phosphate MSO-P consisting of six subunits that constitute a hexameric ring. Overall MtGS structure with location of active sites is presented in Figure 4 [25].

**Figure 5:**
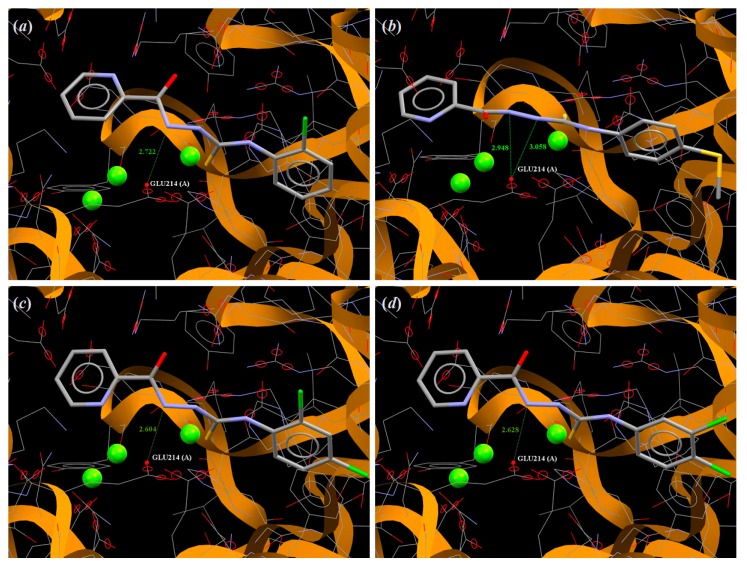
A view of the interaction between (***a***) **2**, (***b***) **3**, (***c***) **4** and (***d***) **5** and the binding site of MtGS e.

The molecular docking study performed for **1**–**25** showed that all investigated ligands bind to the active site of the MtGS receptor with the scoring function ChemPLP presented in Table 5. The values of scoring ChemPLP function vary in the narrow range from 56.13 for **11** to 70.97 for **25,** which shows that all ligands have similar affinity to MtGS. Moreover, the way of connection with the active site of MtGS are similar for all ligands. Most of the investigated compounds, with the exceptions of **7**, **8**, **11** and **23**, show interaction with enzyme via intermolecular hydrogen bonds with the amine group NH of the thiosemicarbazide part of the molecule as a donor of proton and carbonyl group of Glu214 as an acceptor of proton. Other active amino acids in the binding domain in MtGS interacting with analyzed ligands are: Arg364 via N–H…O5 for **1**, **20** and **21**; Arg352 via N–H…O5 for **8**; Lys215 via N–H…O5 for **10** and **23**; and Ser280 via O–H…N(pyridine) for **7**, **11**, **17**, **23** and O–H…O (nitro group) for **15**. The most active against *Mycobacterium tuberculosis* compounds **2**–**5** interacts with the binding domain of MtGS by Glu214 with N4…O(Glu214) distance of 2.722 Å for **2**, 2.948 Å for **3**, 2.604 Å for **4** and 2.628 Å for **5** and N3…O(Glu214) distance of 3.058 Å for **3** (Figure 5). It should be noted that the molecules of compounds **4**, **7**, **11**, **13** and **14** are linked with the active site of MtGs in conformations differing from those observed in the crystalline state (Figure S3; Supplementary Materials), which confirms high flexibility of the carbonylthiosemicarbazide spacer. The docking of the native ligands MSO-P and ADP of MtGS show that they much better fit to the active site of this enzyme by interacting with more amino acid residues in comparison to investigated thiosemicarbazide, as can be seen in Table 5.

## 4. Conclusions

In conclusion, we synthesized several new thiosemicarbazides bearing pyridine ring by the reaction of carboxylic acid hydrazide with appropriate isothiocyanates. The structures of obtained compounds were confirmed by spectroscopic methods and X-ray investigations. The X-ray analyses performed for **4, 7**, **11**, **13** and **14** show that all investigated compounds exist in N1-amino/N3-amino/N4-amino/S2-thione/O5-keto tautomeric form. The conformations of the molecules, stabilized by N–H...X (X = N, Cl, O, S) intramolecular hydrogen bonds, are connected with 2-, 3- and 4-pyridine substituents of carbonylthiosemicarbazide chain. The consequence of the tautomeric form observed in the crystals of **4**, **7**, **11**, **13** and **14** is the net of the intermolecular hydrogen bonds N–H…X (X = O, S, N), which play a key role in crystal packing. All newly obtained thiosemicarbazides were tested in vitro against four *Mycobacterium* strains: *M. H_37_Ra*, *M. phlei*, *M. smegmatis, M. timereck*. The antibacterial activity is strongly connected with the position of the pyridine substituent in relation to the thiosemicarbazide skeleton. The most active against *Mycobacterium tuberculosis* are compounds **2**–**5** with 2-pyridine ring. The molecular docking studies investigating thiosemicarbazides using the enzyme MtGS as their potential biological target indicated that the amino and carbonyl groups of carbonylthiosemicarbazide spacer play an important role in interactions with the active site of MtGS, with Glu214, Glu133, Arg364, Arg352, Lys215 and Ser280 as the most active amino acid residues.

## Figures and Tables

**Table 1:** Selected torsion angles (°) for **4**, **7**, **11**, **13** and **14**.

Torsion Angle	4	7	11	13A	13B	14
C11–N1–C2–N3	166.21(15)	179.4(3)	−176.89(19)	172.9(2)	−171.1(2)	−178.8(3)
N1–C2–N3–N4	162.99(14)	−11.0(5)	3.5(2)	−10.7(3)	9.6(3)	5.1(4)
C2–N3–N4–C5	−154.75(15)	−69.1(4)	92.1(2)	−87.1(3)	86.2(3)	94.4(3)
N3–N4–C5–C51	176.16(13)	169.8(3)	−176.47(15)	−179.79(19)	179.15(19)	178.3(2)
C11–N1–C2–S2	−15.1(2)	1.0(5)	4.4(3)	−9.9(3)	6.0(3)	3.1(4)
N4–N3–C2–S2	−15.7(2)	167.5(3)	−177.69(12)	171.92(16)	−174.28(17)	−176.7(2)
N52–C51–C5–O5	−177.25(15)	-	-	-	-	-
C52–C51–C5–O5	-	35.0(5)	150.14(17)	8.7(3)	−7.0(3)	177.2(3)
C56–C51–C5–O5	5.1(2)	−148.9(4)	−29.1(2)	−175.5(2)	177.9(2)	−7.3(4)

**Table 2:** Hydrogen-bond geometry (Å,°).

D–H…A	D–H	H…A	D…A	D–H…A
**4**				
N1–H1…Cl12	0.79(2)	2.69(2)	2.9681(16)	103.2(18)
N3–H3…O5	0.85(2)	2.34(2)	2.6787(19)	104.3(17)
N4–H4…S2	0.83(2)	2.56(2)	2.9497(16)	109.9(17)
N4–H4…N52	0.83(2)	2.31(2)	2.644(2)	104.8(17)
N1–H1…O5 *^i^*	0.79(2)	2.12(2)	2.849(2)	155(2)
N3–H3…O5 *^i^*	0.85(2)	2.02(2)	2.806(2)	153.2(19)
*i* = −*x*, 2-*y*, −*z*				
**7**				
N1–H1…N4	0.86(2)	2.27(5)	2.658(4)	107(4)
N1–H1…O5 *^i^*	0.86(5)	2.11(5)	2.904(4)	154(4)
N3–H3…N53 *^ii^*	0.80(6)	2.07(5)	2.842(5)	162(5)
N4–H4…S2 *^i^*	0.94(5)	2.44(5)	3.337(3)	160(4)
*i* = *x*, −*y*, ½ + *z*; *ii* = 1 + *x*, *y*, *z*				
**11**				
N1–H1…N4	0.82(2)	2.21(2)	2.637(2)	113(2)
N1–H1…Cl12	0.82(2)	2.49(2)	2.9154(17)	114(2)
N1–H1…O5 *^i^*	0.82(2)	2.49(2)	3.159(2)	140(2)
N3–H3…N53 *^ii^*	0.89(2)	1.98(2)	2.860(2)	172(2)
N4–H4…O5 *^i^*	0.85(3)	2.04(3)	2.792(2)	147(2)
*i* = −1 + *x*, *y*, *z*; *ii* = 1 + *x*, ½ − *y*, ½ + *z*				
**13**				
N1A–H1A…N4A	0.89(4)	2.31(3)	2.697(3)	106(3)
N1B–H1B…N4B	0.81(4)	2.38(3)	2.701(3)	105(3)
N1A–H1A…N54B	0.89(4)	2.05(4)	2.883(3)	155(3)
N1B–H1B…N54A	0.81(4)	2.12(4)	2.896(3)	160(3)
O2–H1…S2A *^i^*	0.82	2.55	3.364(2)	171
N3A–H3A…S2B *^ii^*	0.84(3)	2.48(3)	3.297(2)	166(3)
N3B–H3B…S2A *^iii^*	0.82(3)	2.49(3)	3.291(2)	166(3)
N4A–H4A…O5A *^i^*	0.78(3)	2.17(3)	2.867(2)	149(3)
N4B–H4B…O5B *^iv^*	0.84(3)	2.09(3)	2.849(3)	151(3)
*i* = 3/2−*x*, ½ + −*y*, ½ − *z*; *ii* = 1 + *x*, *y*, *z*; *iii* = −1 + *x*, *y*, *z*; *iv* = ½ − *x*, −1/2 + *y*; ½ − *z*				
**14**				
N1–H1…N4	0.89(4)	2.37(4)	2.711(4)	103(3)
N1–H1…N54 *^i^*	0.89(4)	2.07(4)	2.930(3)	163(4)
N3–H3…S2 *^ii^*	1.03(4)	2.31(4)	3.327(3)	170(3)
N4–H4…O5 *^iii^*	0.86(5)	2.06(5)	2.851(4)	151(4)
*i* = ½-*x*, 3/2-*y*, 1-*z*; *ii* = ½-*x*, 3/2-*y*, -*z*; *ii* = ½-*x*, ½+*y*, ½-*z*				

**Table 3:** Inhibition zone for tested compounds (250 μg of compound was used per well).

No	Inhibition Zone (mm) 250 μg of Compound Per Well			
	*M. H_37_Ra*	*M. phlei*	*M. smegmatis*	*M. timereck*
**1**	28.5	28.1	28.3	29.2
**2**	26.4	23.6	24.1	25.4
**3**	30.5	22.3	21.4	27.6
**4**	29.6	30.1	31.0	29.8
**5**	8.3	14.8	10.7	16.5
**6**	20.7	10.6	17.4	18.8
**7**	20.0	21.0	10.5	20.0
**8**	0.0	0.0	0.0	0.0
**9**	0.0	0.0	0.0	0.0
**10**	26.7	20.7	21.3	19.6
**11**	0.0	0.0	0.0	0.0
**12**	25.9	23.4	22.4	18.4
**13**	0.0	0.0	0.0	0.0
**14**	20.5	18.4	19.6	21.0
**15**	25.5	21.5	18.4	8.3
**16**	0.0	0.0	0.0	0.0
**17**	9.5	9.0	9.8	18.6
**18**	24.0	21.2	22.3	22.2
**19**	13.5	23.8	18.5	16.3
**20**	0.0	8.3	0.0	0.0
**21**	23.3	0.0	0.0	0.0
**22**	20.1	11.1	21.0	22.9
**23**	0.0	9.9	10.2	23.3
**24**	0.0	0.0	0.0	0.0
**25**	8.9	10.8	8.8	0.0
**Rifampicin**	35.4	36.1	33.2	28.0

**Table 4:** Minimal inhibitory concentration for tested compounds.

No	*M. H_37_Ra*		*M. phlei*		*M. smegmatis*		*M. timereck*	
	MIC		MIC		MIC		MIC	
	(µg/mL)	(µM/L)	(µg/mL)	(µM/L)	(µg/mL)	(µM/L)	(µg/mL)	(µM/L)
**1**	15.625	53.82	31.25	107.64	15.625	53.82	15.625	53.82
**2**	15.625	50.93	15.625	50.93	15.625	50.93	15.625	50.93
**3**	15.625	49.07	125	392.56	31.25	98.14	31.25	98.14
**4**	15.625	45.79	31.25	91.58	31.25	91.58	31.25	91.58
**5**	15.625	45.79	31.25	91.58	7.81	22.89	15.625	45.78
**6**	62.5	203.73	500	1629.88	500	1629.88	250	814.94
**7**	15.625	49.07	15.625	49.07	125	392.56	15.625	49.07
**10**	125	465.91	250	931.82	62.5	232.95	125	465.91
**12**	62.5	183.17	31.25	91.58	62.5	183.17	62.5	183.17
**19**	31.25	91.58	62.5	183.17	62.5	183.17	62.5	183.17
**Rifampicin**	0.976	1.18	15.625	18.99	15.625	18.99	3.9	4.74

* Compounds **8**, **9**, **13**–**18** and **20**–**25** MIC > 250 μg/mL.

**Table 5:** The results of the molecular docking of ligands 1–25 to the MtGS.

Compound	ChemPLP Value	Amino Acid Involved in Hydrogen Bonds
**1**	57.60	Glu214, Arg364
**2**	56.89	Glu214
**3**	59.84	Glu214*2
**4**	63.25	Glu214
**5**	64.43	Glu214
**6**	58.49	Glu214
**7**	58.71	Ser280
**8**	57.73	Glu133, Arg352
**9**	64.38	Glu214
**10**	66.12	Glu214, Lys215
**11**	56.13	Ser280
**12**	67.24	Glu214
**13**	56.94	Glu214
**14**	56.74	Glu214
**15**	64.50	Glu214, Ser280
**16**	63.33	Glu214
**17**	65.92	Glu214, Ser280
**18**	64.68	Glu214
**19**	65.80	Glu214
**20**	62.09	Glu214, Arg364
**21**	67.00	Glu214, Arg364
**22**	65.40	Glu214*2
**23**	67.89	Lys215*2, Ser280
**24**	65.72	Glu214
**25**	70.97	Glu214*2
**MSO-P**	127.47	Lys215, Glu219*2, Glu227*2, Glu133*2, Arg352*2
**ADP**	137.10	Lys215, Glu133*2, Arg364, Arg347*2, Glu219.Glu227*2

## Supplementary Materials

The Supplementary Materials are available online.

## References

[1] World Health Organization (2016). Global Tuberculosis Report 2016.

[2] Long R., Ellis E. (2007). Introducing the sixth edition of the Canadian Tuberculosis Standards. Can. J. Infect. Dis. Med. Microbiol..

[3] WHO/Europe | Tuberculosis. http://www.euro.who.int/tb.

[4] European Lung Foundation ELF. http://www.europeanlung.org.

[5] Muller B., Borrell S., Rose G., Gagneux S. (2013). The heterogeneous evolution of multidrug-resistant Mycobacterium tuberculosis. Trends Genet..

[6] Zumla A.I., Gillespie S.H., Hoelscher M., Philips P.P., Cole S.T., Abubakar I., McHugh T.D., Schito M., Maeurer M., Nunn A.J. (2014). New antituberculosis drugs, regimens, and adjunct therapies: Needs, advances, and future prospects. Lancet Infect. Dis..

[7] Hu Y.Q., Zhang S., Zhao F., Gao C., Feng L.S., Lv Z.S., Xu Z., Wu X. (2017). Isoniazid derivatives and their anti-tubercular activity. Eur. J. Med. Chem..

[8] Rane R.A., Naphade S.S., Bangalore P.K., Palkar M.B., Shaikh M.S., Karpoormath R. (2014). Synthesis of novel 4-nitropyrrole-based semicarbazide and thiosemicarbazide hybrids with antimicrobial and anti-tubercular activity. Bioorg. Med. Chem. Lett..

[9] Patel S.R., Gangwal R., Sangamwar A.T., Jain R. (2014). Synthesis, biological evaluation and 3D-QSAR study of hydrazide, semicarbazide and thiosemicarbazide derivatives of 4-(adamantan-1-yl)quinoline as anti-tuberculosis agents. Eur. J. Med. Chem..

[10] Pitucha M., Wos M., Miazga-Karska M., Klimek K., Miroslaw B., Pachuta-Stec A., Gladysz A., Ginalska G. (2016). Synthesis, antibacterial and antiproliferative potential of some new 1-pyridinecarbonyl-4-substituted thiosemicarbazide derivatives. Med. Chem. Res..

[11] Wos M., Miazga-Karska M., Kaczor A.A., Klimek K., Karczmarzyk Z., Kowalczuk D., Wysocki W., Ginalska G., Urbanczyk-Lipkowska Z., Morawiak M. (2017). Novel thiosemicarbazide derivatives with 4-nitrophenyl group as multi-target drugs: Alpha-glucosidase inhibitors with antibacterial and antiproliferative activity. Biomed. Pharmacother..

[12] Pitucha M., Wujec M., Dobosz M. (2013). Synthesis of 3-(pyridin-4-ylmethyl)-4-substituted- 1,2,4-triazoline-5-thione. J. Chin. Chem. Soc..

[13] Moir D.T., Di M., Wong E., Moore R.A., Schweizer H.P., Woods D.E., Bowlin T.L. (2011). Development and application of a cellular, gain-of-signal, bioluminescent reporter screen for inhibitors of type II secretion in *Pseudomonas aeruginosa* and *Burkholderia pseudomallei*. J. Biomol. Screen..

[14] Hu G.Q., Li S., Huang W.L., Wang H. (2002). Synthesis and bioactivity of arecoline derivatives containing 1,2,4-triazone. Youji Huaxue.

[15] Baxendale I.R., Steven V., Ley S.V., Martinelli M. (2005). The rapid preparation of 2-aminosulfonamide-1,3,4-oxadiazoles using polymer-supported reagents and microwave heating. Tetrahedron.

[16] Bhaskar C.S., Vidhale N.N., Berad B.N. (2002). Synthesis of some γ-picolinyl-1,2,4,5-dithiadiazine and their antimicrobial activity. Asian J. Chem..

[17] Riccieri F.M., Porcelli G.A., Castellani P.M. (1967). Thiourea derivatives and their antitubercular activity. Farmaco.

[18] (2015). CrysAlisPRO Software System, Version 1.171.39.16b.

[19] Sheldrick G. (2008). A short history of SHELX. Acta Crystallogr. Sect. A Found. Crystallogr..

[20] Flack H. (1983). On enantiomorph-polarity estimation. Acta Crystallogr. Sect. A Found. Crystallogr..

[21] Farrugia L. (2012). WinGX and ORTEP for Windows: An update. J. Appl. Cryst..

[22] Krajewski W.W., Jones T.A., Mowbray S.L. (2005). Structure of Mycobacterium tuberculosis glutamine synthetase in complex with a transition-state mimic provides functional insights. Proc. Natl. Acad. Sci. USA.

[23] Jones G., Willett P., Glen R.C., Leach A.R., Taylor R. (1997). Development and validation of a genetic algorithm for flexible docking. J. Mol. Biol..

[24] Becke A.D. (1993). Density-Functional Thermochemistry. III. The Role of Exact Exchange. J. Chem. Phys..

[25] Lee C., Yang W., Parr R.G. (1988). Development of the Colle-Salvetti correlation-energy formula into a functional of the electron density. Phys. Rev. B.

[26] Frisch M.J., Trucks G.W., Schlegel H.B., Scuseria G.E., Robb M.A., Cheeseman J.R., Scalmani G., Barone V., Mennucci B., Petersson G.A. (2013). Gaussian 09, Revision, D.01.

[27] Maliszewska-Guz A., Wujec M., Pitucha M., Dobosz M., Chodkowska A., Jagiełło-Wójtowicz E., Mazur L., Kozioł A.E. (2005). Cyclization of 1-{[4-methyl-4*H*-1,2,4-triazol-3-yl) sulfanyl]acetyl}thiosemicarbazides to 1,2,4-triazole and 1,3,4-thiadiazole derivatives and their pharmacological properties. Collect. Czech. Chem. Commun..

[28] Bulut N., Kocyigit U.M., Gecibesler I.H., Dastan T., Karci H., Taslimi P., Durna D.S., Gulcin I., Cetin A. (2018). Synthesis of some novel pyridine compounds containing bis-1,2,4-triazole/thiosemicarbazide moiety and investigation of their antioxidant properties, carbonic anhydrase, and acetylcholinesterase enzymes inhibition profiles. J. Biochem. Mol. Toxicol..

[29] Pitucha M., Polak B., Swatko-Ossor M., Popiołek Ł., Ginalska G. (2010). Determination of the lipophilicity of some new derivatives of thiosemicarbazide and 1,2,4-triazoline-5-thione with potential antituberculosis activity. Croat. Chem. Acta.

[30] Allen F.H., Kennard O., Watson D.G., Brammer L., Orpen A.G., Taylor R. (1987). Tables of bond lengths determined by X-ray and neutron diffraction. Part 1. Bond lengths in organic compounds. J. Chem. Soc. Perkin Trans..

[31] Mowbray L.S., Kathiravan K.M., Pandey A.A., Odell R.L. (2014). Inhibition of Glutamine Synthetase: A Potential Drug Target in Mycobacterium tuberculosis. Molecules.

[32] Harth G., Horwitz M.A. (2003). Inhibition of *Mycobacterium tuberculosis* Glutamine Synthetase as a novel antibiotic strategy against tuberculosis: Demonstration of efficacy in vivo. Infect. Immun..

[33] Harth G., Horwitz M.A. (1999). An inhibitor of exported Mycobacterium tuberculosis glutamine synthetase selectively blocks the growth of pathogenic mycobacteria in axenic culture and in human monocytes: Extracellular proteins as potential novel drug targets. J. Exp. Med..

