# Supplementary material for: Synthesis, In Vitro Screening and Docking Studies of New Thiosemicarbazide Derivatives as Antitubercular Agents
Source: Molecules. 2019 Jan 11;24(2):251. doi: 10.3390/molecules24020251 (PMC6359303; doi:10.3390/molecules24020251)
Supplement: Supplementary file 1 [file molecules-24-00251-s001.pdf]

Supplementary material for publication on line

Synthesis, in vitro screening and docking studies of new thiosemicarbazide derivatives as antitubercular agents

Monika Pitucha[1*], Zbigniew Karczmarzyk[2], Marta Swatko-Ossor[3], Waldemar Wysocki[2], Maciej Wos[1], Kamil Chudzik[1], Grazyna Ginalska[3], Andrzej Fruzinski[4]

[1]Independent Radiopharmacy Unit, Faculty of Pharmacy with Division of Medical Analytics, Medical University of Lublin, Poland; monika.pitucha@umlub.pl (M.P), [2]Department of Chemistry, Siedlce University of Natural Sciences and Humanities, Siedlce, Poland, [3]Department of Biochemistry and Biotechnology, Faculty of Pharmacy with Division of Medical Analytics, Medical University of Lublin, Poland, [4]Institute of General and Ecological Chemistry, Technical University, Łódź, Poland

Experimental data for 1-(pyridine-2-,3-,4-yl)carbonyl-4-substituted thiosemicarbazide (1-19)

4-(2-Fluorophenyl)-1-(pyridin-2 yl)carbonylthiosemicarbazide (**1**)
Yield 87% (white powder); m.p. 182–184°C. ^{1}H NMR (DMSO-d$_6$) δ (ppm): 7.10–7.70 (m, 4H, CH$_{phenyl}$), 7.97–8.72 (m, 3H, CH$_{pyridine}$), 9.48 (s, 1H, NH), 9.81 (s, 1H, NH), 10.78 (s, 1H, NH) [S1].

4-(2-Chlorophenyl)-1-(pyridin-2-yl)carbonylthiosemicarbazide **(2)**
Yield 83% (yellow powder); m.p. 172–174°C. ^{1}H NMR (DMSO-d$_6$) δ (ppm): 7.25–7.65 (m, 4H, CH$_{phenyl}$), 8.03–8.71 (m, 4H, CH$_{pyridine}$), 9.58 (s, 1H, NH); 9.90 (s, 1H, NH); 10.84 (s, 1H, NH) [S1].

4-(4-Methylothiophenyl)-1-(pyridin-2-yl)carbonylthiosemicarbazide **(3)**
Yield 88% (yellow powder); m.p. 184–186°C. ^{1}H NMR (DMSO-d$_6$) δ (ppm): 2.28 (s, 3H, CH$_3$), 7.01–7.25 (m, 4H, CH$_{phenyl}$), 7.51–8.25 (m, 4H, CH$_{pyridine}$), 8.66 (1s, 1H, NH), 10.53 (s, 1H, NH), 12.01 (s, 1H, NH) [S1].

4-(2,4-Dichlorophenyl)-1-(pyridin-2-yl)carbonylthiosemicarbazide (**4**)
Yield 91% (white needles); m.p. 158-160°C. ^{1}H NMR (DMSO-d$_6$) δ (ppm): 7.02–7.53 (m, 3H, CH$_{phenyl}$), 7.93–8.49 (m,4H, CH$_{pyridine}$), 9.12 (s, 1H, NH), 10.56 (s, 1H, NH), 11.89 (s, 1H, NH) [S1].

4-(3,4-Dichlorophenyl)-1-(pyridin-2-yl)carbonylthiosemicarbazide (**5**)
Yield 78% (white powder); m.p. 160-161°C. ^{1}H NMR (DMSO-d$_6$) δ (ppm): 7.56–7.84 (m, 3H, CH$_{phenyl}$), 8.06–8.70 (m,4H, CH$_{pyridine}$), 9.85 (s, 1H, NH), 10.00 (s, 1H, NH), 10.81 (s, 1H, NH). ^{13}C NMR (DMSO-d$_6$) δ (ppm): 123, 125, 127, 129, 130, 138, 139, 148, 149, 164, 181. IR γ_{max} (cm^{-1}): 3318, 3205, 3143, 1685, 1654, 1400 [S2].

4-(2-Chlorophenyl)-1-(pyridin-3-yl)carbonylthiosemicarbazide **(6)**
Yield 83% (yellow powder); m.p. 185-186°C. ^{1}H NMR (DMSO-d$_6$) δ (ppm): 7.28–7.57 (m, 4H, CH$_{phenyl}$), 8.29–9.12 (m,4H, CH$_{pyridine}$), 9.76 (s, 1H, NH), 9.95 (s, 1H, NH), 10.85 (s, 1H, NH). ^{13}C NMR (DMSO-d$_6$) δ (ppm): 123, 127, 128, 129, 131, 132, 136, 137, 149, 152, 165, 182. IR γ_{max} (cm^{-1}): 3250, 3064, 2969, 1739, 1682, 1592 [S3].

4-(4-Methylthiophenyl)-1-(pyridin-3-yl)carbonylthiosemicarbazide (**7**)

Yield 89 % (transparent needles); m.p. 176–177 °C. ^{1}H NMR (DMSO-d_6) δ (ppm): 2.47 (s, 3H, CH_3), 7.23–7.57 (m, 4H, CH_{phenyl}), 8.27–8.76 (m, 4H, $CH_{pyridine}$), 9.11 (s, 1H, NH), 9.81 (s, 1H, NH), 10.76 (s, 1H, NH) [S1].

4-(4-Nitrophenyl)-1-(pyridin-3-yl)carbonylthiosemicarbazide (**8**)

Yield: 72% (orange powder); m.p. 220–222°C. ^{1}H NMR (DMSO-d_6) δ ppm: 7.55-7.91 (m, 4H), 8.21-9.11 (m, 4H), 10.16 (s, 1H), 10.24 (m, 1H), 10.89 (s, 1H) [S4].

***4-[4-(2-Morpholinoethyl)]-1-(pyridin-3-yl)carbonylthiosemicarbazide* (9)**

Yield: 71% (white powder); m.p. 128–129°C. ^{1}H NMR (DMSO-d_6) δ: 2.10-2.48 (m, 4H, 2x$CH_{2morpholina}$); 3.25-3.50 (4H, 2x$CH_{2morpholina}$); 3.56-4.17 (m, 4H, NH-CH_2-CH_2-); 7.48–8.97 (m, 4H, $CH_{pirydyna}$); 9.55 (s, 1H, NH); 10.00 (s, 1H, NH); 10.55 (s, 1H, NH). ^{13}C NMR (DMSO-d_6) δ: 53, 55, 57, 66, 123, 124, 128, 129, 135, 136, 148, 149, 150, 152, 164, 165, 167, 181. IR γ_{max} (cm^{-1}): 3153, 2962, 1662, 1589, 1407. LC/MS (m/z): Calcd. for $C_{13}H_{19}N_5O_2S$, Monoisotopic Mass 309.1260 Da, $[M+H]^+$ 310.1332 Da, Measured Mass 309.1264.

***4-[Methoxycarbonylmethyl)]-1-(pyridin-3-yl)carbonylthiosemicarbazide* (10)**

Yield: 74% (white powder); 178-179°C. ^{1}H NMR (DMSO-d_6) δ ppm: ^{1}H NMR (DMSO-d_6) δ ppm: 3.63 (s, 3H, CH_3); 4.20 (d, 2H, CH_2); 7.82–7.84 (m, 2H, CH_{aromat}); 8.54 (s, 1H, NH); 8.76-8.78 (m, 2H, CH_{aromat}); 9.76 (s, 1H, NH); 10.83 (s, 1H, NH). ^{13}C NMR (DMSO-d_6) δ: 45, 52, 122, 139, 150, 164, 170, 183. IR γ_{max} (cm^{-1}): 3101, 2929, 1662, 1586, 1421. LC/MS (m/z): Calcd. for $C_{10}H_{12}N_4O_3S$, Monoisotopic Mass 268.0630 Da, $[M+H]^+$ 269.070287 Da, Measured Mass 268.0633.

4-(2,4-Dichlorophenyl)-1-(pyridin-3-yl)carbonyl thiosemicarbazide (**11**)

Yield 92 % (transparent needles); m.p. 196–197°C. ^{1}H NMR (DMSO-d_6) δ ppm: 7.37–7.68 (m, 3H, CH_{phenyl}), 8.14–9.11 (m, 4H, $CH_{pyridine}$), 9.95 (s, 1H, NH), 10.08 (s, 1H, NH), 10.83 (s, 1H, NH). ^{13}C NMR (DMSO-d_6) δ ppm: 123, 126, 127, 128, 129, 130, 135, 136, 139, 148, 149, 152, 165, 181[S1].

***4-(3,4-Dichlorophenyl)-1-(pyridin-3-yl)carbonylthiosemicarbazide* (12)**

Yield 75% ((yellow needles); m.p. 146-147°C. ^{1}H NMR (DMSO-d_6) δ ppm: 7.53–7.81 (m, 3H, CH_{phenyl}), 8.14–9.11 (m, 4H, $CH_{pyridine}$), 9.94 (s, 1H, NH), 10.08 (s, 1H, NH), 10.86 (s, 1H, NH). ^{13}C NMR (DMSO-d_6) δ: 123, 127, 128, 129, 132, 133, 136, 149, 152, 165, 182. IR γ_{max} (cm^{-1}): 3356, 3077, 1654, 1590, 1420 [S5].

4-(2-Fluorophenyl)-1-(pyridin-4-yl)carbonylthiosemicarbazide (**13**)

Yield 78% (yellow powder); m.p. 202–204°C. ^{1}H NMR (DMSO-d_6) δ ppm: 7.18–7.31 (m, 4H, CH_{phenyl}), 7.86–8.78 (m, 4H, $CH_{pyridine}$), 9.70 (s, 1H, NH), 9.99 (s, 1H, NH), 10.94 (s, 1H, NH) [S1].

4-(2-Chlorophenyl)-1-(pyridin-4-yl)carbonylthiosemicarbazide (**14**)

Yield 74 % (yellow powder); m.p. 224–226°C. ^{1}H NMR (DMSO-d_6) δ ppm: 7.28–7.87 (m, 4H, CH_{phenyl}), 8.77–8.78 (m, 4H, $CH_{pyridine}$), 9.75 (s, 1H, NH), 9.96 (s, 1H, NH), 10.93 (s, 1H, NH). ^{13}C NMR (DMSO-d_6) δ: 122, 127, 128, 129, 131, 132, 137, 140, 150, 165, 182. IR γ_{max} (cm^{-1}): 3257, 3109, 1739, 1677, 1406 [S6].

4-(2-Nitrophenyl)-1-(pyridin-4-yl)carbonylthiosemicarbazide (**15**)

Yield: 62% (orange powder); m.p. 134–136°C. ^{1}H NMR (DMSO-d_6) δ ppm: 7.44-8.04 (m, 4H); 8.19-8.39 (m, 4H); 10.18 (s, 1H); 10.20 (s, 1H); 11.07 (s, 1H) [S4].

***4-(4-Methylthiophenyl)-1-(pyridin-4-yl)carbonylthiosemicarbazide* (16)**

Yield 86% (yellow powder); m.p. 197–198°C. ^{1}H NMR (DMSO-d_6) δ ppm: 2.47 (s, 3H, CH3), 7.23–7.86 (m, 4H, CH_{phenyl}), 8.77–8.78 (m, 4H, $CH_{pyridine}$), 9.83 (s, 2H, NH), 10.86 (s, 1H, NH) [S1].

***4-[4-(2-Morpholinoethyl)]-1-(pyridin-4-yl)carbonylthiosemicarbazide* (17)**

Yield: 70% (yellow powder); m.p. 176–178°C. ^{1}H NMR (DMSO-d_6) δ: 2.38-2.44 (m, 4H, $2xCH_{2morpholina}$); 3.35-3.36 (m, 4H, $2xCH_{2morpholina}$); 3.51-3.57 (m, 4H, NH-CH_2-CH_2-); 7.81–8.77 (m, 4H, $CH_{pirydyna}$); 8.01 (s, 1H, NH); 9.46 (s, 1H, NH); 10.71 (s, 1H, NH). ^{13}C NMR (DMSO-d_6) δ: 41, 53, 57, 66, 122, 139, 150, 164. IR γ_{max} (cm^{-1}): 3306, 3147, 1735, 1677, 1408. LC/MS (m/z): Calcd. for $C_{13}H_{19}N_5O_2S$, Monoisotopic Mass 309.1260 Da, $[M+H]^+$ 310.1332 Da, Measured Mass 309.1262.

***4-(2,4-Dichlorophenyl)-1-(pyridin-4-yl)carbonylthiosemicarbazide* (18)**

Yield 84 % (white powder); m.p. 164–166 °C. ^{1}H NMR (DMSO-d_6) δ ppm: 7.37–7.45 (m, 3H, CH_{phenyl}), 7.68–8.78 (m, 4H, $CH_{pyridine}$), 9.76 (s, 1H, NH), 10.05 (s, 1H, NH), 10.95 (s, 1H, NH) [S1].

***4-(3,4-Dichlorophenyl)-1-(pyridin-4-yl)carbonylthiosemicarbazide* (19)**

Yield 85 % (yellow powder); m.p. 182–183°C. ^{1}H NMR (DMSO-d_6) δ ppm: 7.42–7.86 (m, 3H, CH_{phenyl}), 8.69–8.78 (m, 4H, $CH_{pyridine}$), 9.76 (s, 1H, NH), 10.04 (s, 1H, NH), 10.95 (s, 1H, NH). ^{13}C NMR (DMSO-d_6) δ: 122, 129, 131, 132, 133, 134, 148, 150, 169. IR γ_{max} (cm^{-1}): 3202, 3027, 1735, 1592, 1414 [S7].

Experimental data for 1-(pyridin-4-ylacetyl)-4-substituted thiosemicabazide (20-25)

4-Phenyl-1-(pyridin-4-ylacetyl)thiosemicarbazide (20)

Yield 78% (yellow powder); m.p. 116–118 °C. ^{1}H NMR (DMSO-d_6) δ ppm: 3.58 (s, 2H, CH_2), 7.19-7.45 (m, 5H, CH_{phenyl}), 8.46-8.51 (m, 4H, $CH_{pyridine}$), 9.73, 10.01, 10.25 (3s, 3H, 3NH) [S8].

4-(2-Fluorophenyl)-1-(pyridin-4-ylacetyl)thiosemicarbazide (21)

Yield 68% (yellow powder); m.p. 148–149 °C. ^{1}H NMR (DMSO-d_6) δ ppm: 3.58 (s, 2H, CH_2), 7.18-7.39 (m, 4H, CH_{phenyl}), 8.50-8.52 (m, 4H, $CH_{pyridine}$), 9.55, 9.80, 10.33 (3s, 3H, 3NH) [S8].

4-(4-Bromophenyl)-1-(pyridin-4-ylacetyl)thiosemicarbazide (22)

Yield 77% (yellow powder); mp. 220-222 °C. ^{1}H NMR (DMSO-d_6) δ ppm: 3.93 (s, 2H, CH_2), 7.03–7.74 (m,4H, CH_{phenyl}), 8.33-8.58 (m, 4H, $CH_{pyridine}$), 9.75; 10.27; 13.90 (3s, 3H, 3NH). ^{13}C NMR (DMSO-d_6) δ ppm: 31; 122; 123; 124; 125; 130; 130; 132; 133; 137 ; 139; 143; 148; 151; 168. IR γ_{max} (cm^{-1}): 3166, 1688, 1400. LC/MS (m/z): Calcd. for $C_{14}H_{13}BrN_4OS$, Monoisotopic Mass 363.9993 Da, $[M+H]^+$ 365.0066 Da, Measured Mass 363.9997.

4-Methoxycarbonylmethyl-1-(pyridin-4-ylacetyl)thiosemicarbazide (23)

Yield 73% (white powder); m.p. 178–180 °C. ^{1}H NMR (DMSO-d_6) δ ppm: 3.36 (s, 2H, CH_2), 3.63 (s, 3H, CH_3), 4.20 (s, 2H, CH_2), 7.82-8.76 (m, 4H, $CH_{pyridine}$), 8.54; 9.76; 10.82 (3s, 3H, 3NH). ^{13}C NMR (DMSO-d_6) δ ppm: 45, 52, 122, 139, 150, 164, 170, 182. LC/MS (m/z): Calcd. for $C_{11}H_{14}N_4O_3S$, Monoisotopic Mass 282.0787 Da, $[M+H]^+$ 283.0860 Da.

4-(2,4-Dichlorophenyl)-1-(pyridin-4-ylacetyl)thiosemicarbazide (24)

Yield 72% (yellow powder); m.p. 150–151 °C. ^{1}H NMR (DMSO-d_6) δ ppm: 3.64 (s. 2H, CH_2), 7.33–7.50 (m, 3H, CH_{phenyl}), 7.73–8.56 (m, 4H, $CH_{pyridine}$), 9.64 (s, 1H, NH), 9.92 (s, 1H, NH), 10.39 (s, 1H, NH). ^{13}C NMR (DMSO-d_6) δ: 56, 122, 124, 125, 127, 128, 129, 130, 132, 133, 144, 149, 168, 179, 182. IR γ_{max} (cm^{-1}): 3231, 2929, 1682, 1609, 1421. LC/MS (m/z): Calcd. for $C_{14}H_{12}Cl_2N_4OS$, Monoisotopic Mass 354.0109 Da, $[M+H]^+$ 355.0182 Da, Measured Mass 354.1012.

4-(3,4-Dichlorophenyl)-1-(pyridin-4-ylacetyl)thiosemicarbazide (25)

Yield 75% (white powder); m.p. 161–163 °C. ^{1}H NMR (DMSO-d_6) δ ppm: 3.59 (s, 2H, CH_2), 7.35–7.74 (m, 3H, CH_{phenyl}), 7.83–8.51 (m, 4H, $CH_{pyridine}$), 9.88 (s, 1H, NH), 10.16 (s, 1H, NH), 10.21 (s, 1H, NH). ^{13}C NMR (DMSO-d_6) δ: 35, 123, 126, 127, 130, 131, 137, 139, 148, 150, 170, 181. IR γ_{max} (cm^{-1}): 3333, 3074, 1668, 1590, 1427. LC/MS (m/z): Calcd. for $C_{14}H_{12}Cl_2N_4OS$, Monoisotopic Mass 354.0109 Da, $[M+H]^+$ 355.0182 Da, Measured Mass 354.0113.

X-ray analysis: crystal structures of 4, 7, 11, 13 and 14

In the crystal structure of **4** the inversion related molecules form molecular dimers through the pair bifurcated intermolecular hydrogen bonds N1–H1…O5 and N3–H3…O5. Moreover, the benzene rings belonging to the inversion related molecules partially overlap each other with the π…π distance of 3.4567(7) Å characteristic for the overlapping π-aromatic ring systems.

In the crystal of **7** the molecules related by c glide planes are linked into molecular chains parallel to Z crystallographic axis *via* the pair of intermolecular hydrogen bonds N1–H1…O5 and N4–H4…S2. Additionally, the molecules related by translation *a* form molecular chains by N3–H3…N53 hydrogen bond. The combination of these two types of chains gives molecular planes parallel to (010) crystallographic plane.

In the crystal structure of **11** the net of intermolecular hydrogen bonds gives the molecular planes parallel to the (010) crystallographic plane as a combination of two molecular chains. The first one is formed by molecules related by translation *a* and connected via N1–H1…O5 and N4–H4…O5 bifurcated hydrogen bonds, while the second one is created by molecules related by *c* glide planes through N3 – H3…N53 hydrogen bond.

In the crystal of **13** the molecules A and B from asymmetric part of the unit cell form the molecular dimer using N1A–H1A…N54B and N1B–H1B…N54A hydrogen bonds. The π…π interaction between pyridine rings within this dimer is observed; the centroid-to-centroid separation and the angle between the overlapping planes of these rings are 3.4559(13) Å and 0.57(11)°, respectively. Moreover, the intermolecular hydrogen bonds N4A–H4A….O5A and N4B–H4B…O5B linking molecules A and B related by 2_1 axis (independently of each other) into molecular chains along b direction. Similar chains are formed by the A and 2-propanol molecules *via* O2–H1…S2A hydrogen bond.

In the crystal of **14** the molecules related by centers of inversion along c translation are linked into molecular chains *via* two pairs of intermolecular hydrogen bonds N1–H1…N54 and N3–H3…S2. The similar molecular chains are created also by molecules related by 2-fold axis in *b* direction resulting in molecular layers parallel to (100) crystallographic plane. Moreover, the pyridine rings belonging to the inversion related molecules partially overlap each other with centroid-to-centroid separation of 3.4259(16) Å, π…π distance of 3.1808(11) Å and slippage of 1.273 Å.

Theoretical calculations

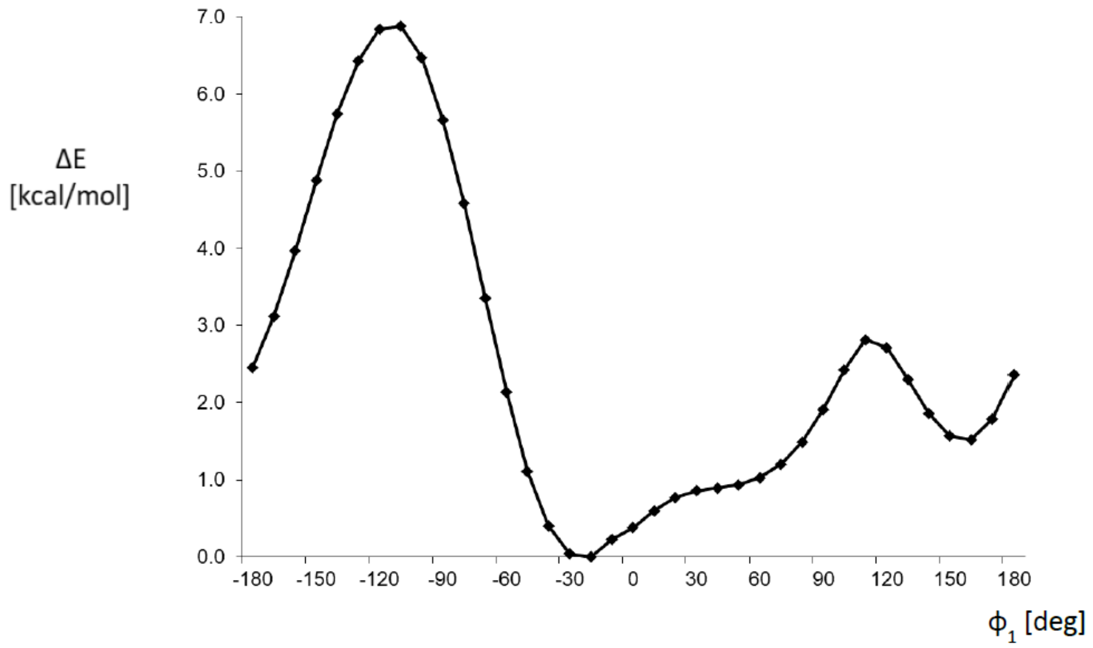

Fig. 1S. The energy effect upon C2–N3 (φ_1 = N1–C2–N3–N4) rotation calculated for **4** using AM1 method.

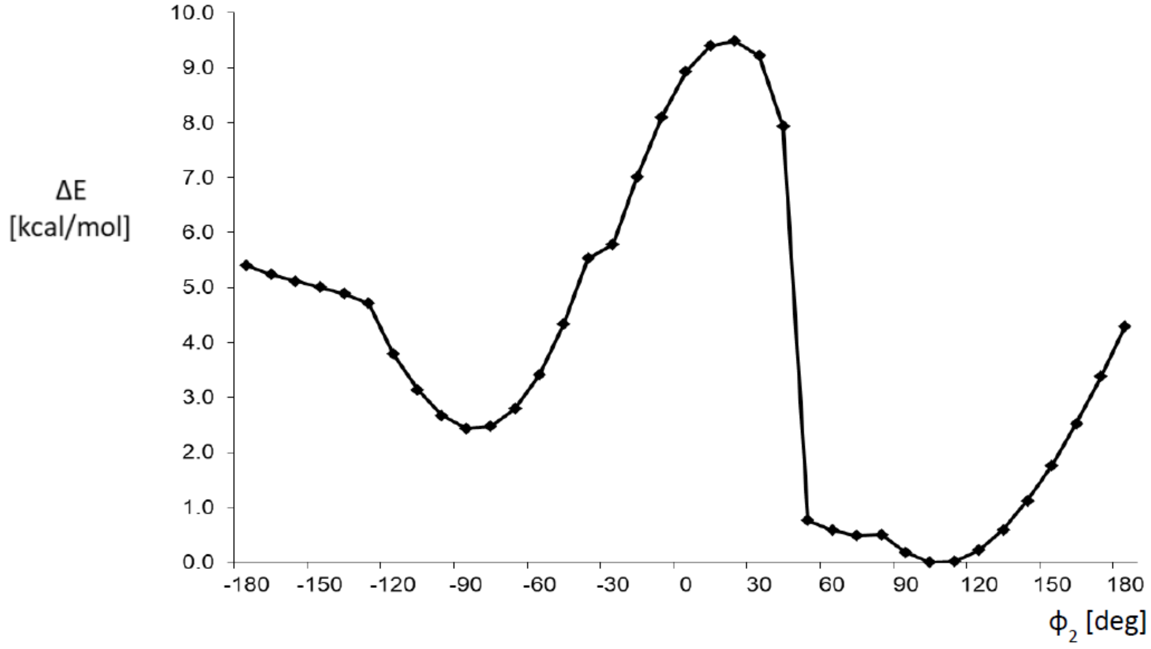

Fig. 2S. The energy effect upon N3–N4 (φ_2 = C2–N3–N4–C5) rotation calculated for **4** using AM1 method.

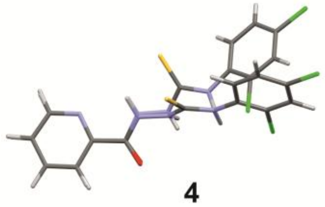

4

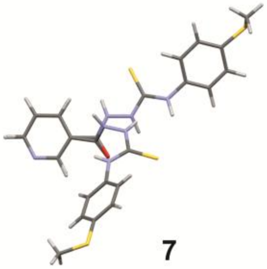

7

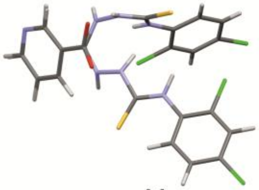

11

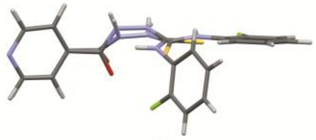

13

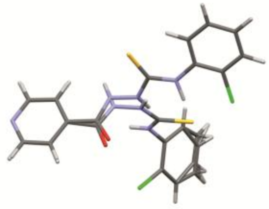

14

Fig. 3S. Overlay of molecules **4**, **7**, **11**, **13** and **14** in the conformations observed in the crystalline state and in the active site of MtGS by least-squares fitting of the atoms of pyridine systems (RMS = 0.0066 Å for **4**, 0.0109 Å for **7**, 0.0198 Å for **11**, 0.0159 Å for **13** and 0.0164 Å for **14**).

References

[S1] Pitucha, M.; Wos, M.; Miazga-Karska, M.; Klimek, K.; Miroslaw, B.; Pachuta-Stec, A.; Gladysz, A.; Ginalska, G., Synthesis, antibacterial and antiproliferative potential of some new 1-pyridinecarbonyl-4-substituted thiosemicarbazide derivatives. *Med Chem Res.* **2016**, 25, pp. 1666-1677, DOI: 10.1007/s00044-016-1599-6.

[S2] Moir, D.T.; Di, M.; Wong, E.; Moore, R.A.; Schweizer, H.P.; Woods, D.E.; Bowlin, T.L., Development and application of a cellular, gain-of-signal, bioluminescent reporter screen for inhibitors of type II secretion in Pseudomonas aeruginosa and Burkholderia pseudomallei. *J. Biomolec. Screening* 2011, 16(7), pp. 694-705, DOI: 10.1177/1087057111408605.

[S3] Guo-qiang;, H.; Wen-long, H.; Hui-bin, Z.; Hai, W., Synthesis and bioactivity of arecoline derivatives containing 1,2,4-triazone. *Youji Huaxue* **2002**, 22(9), pp. 667-671.

[S4] Wos, M.; Miazga-Karska, M.; Kaczor, A. A.; Klimek, K.; Karczmarzyk, Z.; Kowalczuk, D.; Wysocki, W.; Ginalska, G.; Urbanczyk-Lipkowska, Z.; Morawiak, M.; Pitucha, M., Novel thiosemicarbazide derivatives with 4-nitrophenyl group as multi-target drugs: alpha-glucosidase inhibitors with antibacterial and antiproliferative activity. *Biomed Pharmacother.* **2017**, 93, pp. 1269-1276, DOI: 10.1016/j.biopha.2017.07.049.

[S5] Baxendale, I.R.; Steven V.Ley, S.V.; Martinelli, M., The rapid preparation of 2-aminosulfonamide-1,3,4-oxadiazoles using polymer-supported reagents and microwave heating. *Tetrahedron* **2005**, 61(22), pp. 5323-5349.

[S6] Bhaskar, C.S.; Vidhale, N.N.; Berad, B.N., Synthesis of some γ-picolinyl-1,2,4,5-dithiadiazine and their antimicrobial activity. *Asian J. Chem.* **2002**, 14(1), pp. 162-168.

[S7] Riccieri, F.M.; Porcelli, G.A.; Castellani P.M., Thiourea derivatives and their antitubercular activity. *Farmaco* **1967**, 22(2), p.p. 114-20.

[S8] Pitucha, M.; Wujec, M.; Dobosz, M., Synthesis of 3-(pyridin-4-ylmethyl)-4-substituted- 1,2,4-triazoline-5-thione. *J. Chin. Chem. Soc.* **2013**, 54 (1), pp. 69-73.

X-ray structure determination

CIF for 4

```
data_shelx

_audit_creation_method            'SHELXL-2014/7'
_shelx_SHELXL_version_number      '2014/7'
_chemical_name_systematic         ?
_chemical_name_common             ?
_chemical_melting_point           ?
_chemical_formula_moiety          ?
_chemical_formula_sum             'C13 H10 Cl2 N4 O S'
_chemical_formula_weight          341.21

loop_
 _atom_type_symbol
 _atom_type_description
 _atom_type_scat_dispersion_real
 _atom_type_scat_dispersion_imag
 _atom_type_scat_source
 'C'  'C'   0.0181   0.0091
 'International Tables Vol C Tables 4.2.6.8 and 6.1.1.4'
 'H'  'H'   0.0000   0.0000
 'International Tables Vol C Tables 4.2.6.8 and 6.1.1.4'
 'Cl'  'Cl'   0.3639   0.7018
 'International Tables Vol C Tables 4.2.6.8 and 6.1.1.4'
 'N'  'N'   0.0311   0.0180
 'International Tables Vol C Tables 4.2.6.8 and 6.1.1.4'
 'O'  'O'   0.0492   0.0322
 'International Tables Vol C Tables 4.2.6.8 and 6.1.1.4'
 'S'  'S'   0.3331   0.5567
 'International Tables Vol C Tables 4.2.6.8 and 6.1.1.4'

_space_group_crystal_system       triclinic
_space_group_IT_number            2
_space_group_name_H-M_alt         'P -1'
_space_group_name_Hall            '-P 1'

_shelx_space_group_comment
;
The symmetry employed for this shelxl refinement is uniquely defined
by the following loop, which should always be used as a source of
symmetry information in preference to the above space-group names.
They are only intended as comments.
;

loop_
 _space_group_symop_operation_xyz
 'x, y, z'
 '-x, -y, -z'

_cell_length_a                    7.4539(2)
_cell_length_b                    7.8084(2)
_cell_length_c                    14.3223(2)
_cell_angle_alpha                 104.442(2)
_cell_angle_beta                  92.188(2)
_cell_angle_gamma                 112.898(2)
_cell_volume                      735.05(3)
_cell_formula_units_Z             2
_cell_measurement_temperature     293(2)
_cell_measurement_reflns_used     214
_cell_measurement_theta_min       7.9
_cell_measurement_theta_max       77.4

_exptl_crystal_description        prism
_exptl_crystal_colour             colourless
_exptl_crystal_density_meas       ?
_exptl_crystal_density_method     ?
```

```
_exptl_crystal_density_diffrn     1.542
_exptl_crystal_F_000             348
_exptl_transmission_factor_min    ?
_exptl_transmission_factor_max    ?
_exptl_crystal_size_max          0.50
_exptl_crystal_size_mid          0.16
_exptl_crystal_size_min          0.03
_exptl_absorpt_coefficient_mu     5.339
_shelx_estimated_absorpt_T_min    ?
_shelx_estimated_absorpt_T_max    ?
_exptl_absorpt_correction_type    multi-scan
_exptl_absorpt_correction_T_min   0.3443
_exptl_absorpt_correction_T_max   1.0000
_exptl_absorpt_process_details
;
CrysAlisPro 1.171.39.16b (Rigaku Oxford Diffraction, 2015)
Empirical absorption correction using spherical harmonics,
 implemented in SCALE3 ABSPACK scaling algorithm.
;
_exptl_absorpt_special_details    ?
_diffrn_ambient_temperature       293(2)
_diffrn_radiation_wavelength      1.54178
_diffrn_radiation_type            CuK\a
_diffrn_source                   'micro-focus sealed X-ray tube'
_diffrn_measurement_device        'four-circle diffractometer'
_diffrn_measurement_device_type   'XtaLAB Synergy, Dualflex, Pilatus 300K'
_diffrn_measurement_method        '\w scans'
_diffrn_detector_area_resol_mean  ?
_diffrn_reflns_number            14710
_diffrn_reflns_av_unetI/netI      0.0231
_diffrn_reflns_av_R_equivalents   0.0416
_diffrn_reflns_limit_h_min        -9
_diffrn_reflns_limit_h_max        8
_diffrn_reflns_limit_k_min        -9
_diffrn_reflns_limit_k_max        9
_diffrn_reflns_limit_l_min        -18
_diffrn_reflns_limit_l_max        17
_diffrn_reflns_theta_min         6.307
_diffrn_reflns_theta_max         78.646
_diffrn_reflns_theta_full        67.679
_diffrn_measured_fraction_theta_max   0.959
_diffrn_measured_fraction_theta_full  0.998
_diffrn_reflns_Laue_measured_fraction_max    0.959
_diffrn_reflns_Laue_measured_fraction_full   0.998
_diffrn_reflns_point_group_measured_fraction_max   0.959
_diffrn_reflns_point_group_measured_fraction_full  0.998
_reflns_number_total             3041
_reflns_number_gt                2964
_reflns_threshold_expression      'I > 2\s(I)'
_reflns_Friedel_coverage          0.000
_reflns_Friedel_fraction_max      .
_reflns_Friedel_fraction_full     .

_reflns_special_details
;
 Reflections were merged by SHELXL according to the crystal
 class for the calculation of statistics and refinement.

 _reflns_Friedel_fraction is defined as the number of unique
 Friedel pairs measured divided by the number that would be
 possible theoretically, ignoring centric projections and
 systematic absences.
;

_computing_data_collection        'CrysAlisPro 1.171.39.16b (Rigaku OD, 2015)'
_computing_cell_refinement        'CrysAlisPro 1.171.39.16b (Rigaku OD, 2015)'
_computing_data_reduction         'CrysAlisPro 1.171.39.16b (Rigaku OD, 2015)'
_computing_structure_solution     'SHELXS-2013/1 (Sheldrick, 2014)'
_computing_structure_refinement   'SHELXL-2014/7 (Sheldrick, 2014)'
_computing_molecular_graphics     'ORTEP3 for Windows'
```

```
_computing_publication_material   'SHELXL-2014/7 and WINGX'
_refine_special_details           ?
_refine_ls_structure_factor_coef  Fsqd
_refine_ls_matrix_type            full
_refine_ls_weighting_scheme       calc
_refine_ls_weighting_details
'w=1/[\s^2^(Fo^2^)+(0.0526P)^2^+0.4585P] where P=(Fo^2^+2Fc^2^)/3'
_atom_sites_solution_primary      difmap
_atom_sites_solution_secondary    difmap
_atom_sites_solution_hydrogens    difmap
_refine_ls_hydrogen_treatment     mixed
_refine_ls_extinction_method      'SHELXL-2014/7 (Sheldrick 2014'
_refine_ls_extinction_coef        0.0061(7)
_refine_ls_extinction_expression
 'Fc^*^=kFc[1+0.001xFc^2^\l^3^/sin(2\q)]^-1/4^'
_refine_ls_number_reflns          3041
_refine_ls_number_parameters      200
_refine_ls_number_restraints      0
_refine_ls_R_factor_all           0.0344
_refine_ls_R_factor_gt            0.0336
_refine_ls_wR_factor_ref          0.0924
_refine_ls_wR_factor_gt           0.0919
_refine_ls_goodness_of_fit_ref    1.068
_refine_ls_restrained_S_all       1.068
_refine_ls_shift/su_max           0.001
_refine_ls_shift/su_mean          0.000

loop_
 _atom_site_label
 _atom_site_type_symbol
 _atom_site_fract_x
 _atom_site_fract_y
 _atom_site_fract_z
 _atom_site_U_iso_or_equiv
 _atom_site_adp_type
 _atom_site_occupancy
 _atom_site_site_symmetry_order
 _atom_site_calc_flag
 _atom_site_refinement_flags_posn
 _atom_site_refinement_flags_adp
 _atom_site_refinement_flags_occupancy
 _atom_site_disorder_assembly
 _atom_site_disorder_group
S2 S -0.04981(6) 0.93024(6) 0.79299(3) 0.01851(13) Uani 1 1 d . . . . .
Cl12 Cl 0.44256(6) 0.63514(6) 0.65676(3) 0.02420(14) Uani 1 1 d . . . . .
Cl14 Cl -0.21596(8) 0.05515(6) 0.43692(3) 0.03278(15) Uani 1 1 d . . . . .
O5 O 0.50820(17) 1.21192(17) 1.07306(9) 0.0205(3) Uani 1 1 d . . . . .
N1 N 0.1722(2) 0.7249(2) 0.78786(10) 0.0170(3) Uani 1 1 d . . . . .
H1 H 0.269(3) 0.724(3) 0.8115(16) 0.020 Uiso 1 1 d . U . . .
N3 N 0.2197(2) 0.95531(19) 0.92822(10) 0.0162(3) Uani 1 1 d . . . . .
H3 H 0.318(3) 0.935(3) 0.9452(15) 0.019 Uiso 1 1 d . U . . .
N4 N 0.2228(2) 1.13209(19) 0.97884(10) 0.0154(3) Uani 1 1 d . . . . .
H4 H 0.135(3) 1.162(3) 0.9603(15) 0.018 Uiso 1 1 d . U . . .
N52 N 0.2059(2) 1.4666(2) 1.06302(10) 0.0188(3) Uani 1 1 d . . . . .
C2 C 0.1176(2) 0.8650(2) 0.83649(11) 0.0155(3) Uani 1 1 d . . . . .
C5 C 0.3727(2) 1.2538(2) 1.04886(11) 0.0146(3) Uani 1 1 d . . . . .
C11 C 0.0750(2) 0.5769(2) 0.70041(11) 0.0159(3) Uani 1 1 d . . . . .
C12 C 0.1866(2) 0.5137(2) 0.63525(12) 0.0171(3) Uani 1 1 d . . . . .
C13 C 0.0989(3) 0.3545(2) 0.55345(12) 0.0209(4) Uani 1 1 d . . . . .
H13 H 0.1749 0.3128 0.5112 0.025 Uiso 1 1 calc R U . . .
C14 C -0.1039(3) 0.2599(2) 0.53654(12) 0.0215(4) Uani 1 1 d . . . . .
C15 C -0.2195(3) 0.3233(3) 0.59699(13) 0.0221(4) Uani 1 1 d . . . . .
H15 H -0.3562 0.2609 0.5828 0.027 Uiso 1 1 calc R U . . .
C16 C -0.1295(3) 0.4808(2) 0.67890(12) 0.0193(3) Uani 1 1 d . . . . .
H16 H -0.2067 0.5230 0.7201 0.023 Uiso 1 1 calc R U . . .
C51 C 0.3664(2) 1.4455(2) 1.09387(11) 0.0141(3) Uani 1 1 d . . . . .
C53 C 0.5146(2) 1.7693(2) 1.19904(12) 0.0184(3) Uani 1 1 d . . . . .
H53 H 0.6173 1.8702 1.2443 0.022 Uiso 1 1 calc R U . . .
C54 C 0.3504(3) 1.7936(2) 1.16894(12) 0.0210(4) Uani 1 1 d . . . . .
H54 H 0.3403 1.9110 1.1936 0.025 Uiso 1 1 calc R U . . .
```

C55 C 0.1998(3) 1.6388(3) 1.10085(13) 0.0227(4) Uani 1 1 d . . . . .
H55 H 0.0896 1.6562 1.0807 0.027 Uiso 1 1 calc R U . . .
C56 C 0.5244(2) 1.5914(2) 1.16058(11) 0.0161(3) Uani 1 1 d . . . . .
H56 H 0.6336 1.5710 1.1791 0.019 Uiso 1 1 calc R U . . .

loop_
 _atom_site_aniso_label
 _atom_site_aniso_U_11
 _atom_site_aniso_U_22
 _atom_site_aniso_U_33
 _atom_site_aniso_U_23
 _atom_site_aniso_U_13
 _atom_site_aniso_U_12
S2 0.0172(2) 0.0197(2) 0.0176(2) -0.00037(15) -0.00255(15) 0.01044(16)
Cl12 0.0181(2) 0.0274(2) 0.0252(2) 0.00222(17) 0.00428(16) 0.01057(17)
Cl14 0.0443(3) 0.0199(2) 0.0190(2) -0.00743(16) -0.00413(18) 0.0062(2)
O5 0.0208(6) 0.0187(6) 0.0202(6) -0.0021(5) -0.0036(5) 0.0116(5)
N1 0.0149(6) 0.0174(7) 0.0159(7) -0.0030(5) -0.0032(5) 0.0088(5)
N3 0.0187(7) 0.0130(6) 0.0156(6) -0.0020(5) -0.0016(5) 0.0092(5)
N4 0.0167(7) 0.0123(6) 0.0157(6) -0.0021(5) -0.0015(5) 0.0083(5)
N52 0.0203(7) 0.0157(7) 0.0182(7) -0.0005(5) -0.0030(5) 0.0090(6)
C2 0.0133(7) 0.0135(7) 0.0158(7) 0.0011(6) 0.0011(6) 0.0036(6)
C5 0.0149(7) 0.0145(7) 0.0130(7) 0.0013(6) 0.0022(6) 0.0063(6)
C11 0.0197(8) 0.0135(7) 0.0127(7) -0.0001(6) -0.0009(6) 0.0076(6)
C12 0.0174(8) 0.0164(8) 0.0170(7) 0.0025(6) 0.0009(6) 0.0080(6)
C13 0.0304(9) 0.0181(8) 0.0161(8) 0.0020(6) 0.0046(7) 0.0136(7)
C14 0.0308(9) 0.0128(8) 0.0140(7) -0.0012(6) -0.0024(6) 0.0054(7)
C15 0.0203(8) 0.0178(8) 0.0201(8) 0.0015(6) -0.0019(6) 0.0022(7)
C16 0.0201(8) 0.0165(8) 0.0169(8) -0.0001(6) 0.0013(6) 0.0060(6)
C51 0.0163(7) 0.0128(7) 0.0126(7) 0.0019(6) 0.0021(6) 0.0064(6)
C53 0.0209(8) 0.0130(7) 0.0153(7) 0.0003(6) 0.0006(6) 0.0034(6)
C54 0.0277(9) 0.0134(8) 0.0208(8) -0.0002(6) 0.0016(7) 0.0106(7)
C55 0.0249(9) 0.0178(8) 0.0247(8) 0.0007(7) -0.0040(7) 0.0121(7)
C56 0.0152(7) 0.0158(7) 0.0147(7) 0.0013(6) 0.0005(6) 0.0056(6)

_geom_special_details
;
 All esds (except the esd in the dihedral angle between two l.s. planes)
 are estimated using the full covariance matrix. The cell esds are taken
 into account individually in the estimation of esds in distances, angles
 and torsion angles; correlations between esds in cell parameters are only
 used when they are defined by crystal symmetry. An approximate (isotropic)
 treatment of cell esds is used for estimating esds involving l.s. planes.
;

loop_
 _geom_bond_atom_site_label_1
 _geom_bond_atom_site_label_2
 _geom_bond_distance
 _geom_bond_site_symmetry_2
 _geom_bond_publ_flag
S2 C2 1.6730(16) . ?
Cl12 C12 1.7422(17) . ?
Cl14 C14 1.7383(17) . ?
O5 C5 1.238(2) . ?
N1 C2 1.355(2) . ?
N1 C11 1.409(2) . ?
N1 H1 0.79(2) . ?
N3 C2 1.359(2) . ?
N3 N4 1.3805(18) . ?
N3 H3 0.85(2) . ?
N4 C5 1.330(2) . ?
N4 H4 0.83(2) . ?
N52 C55 1.337(2) . ?
N52 C51 1.343(2) . ?
C5 C51 1.495(2) . ?
C11 C16 1.393(2) . ?
C11 C12 1.399(2) . ?
C12 C13 1.388(2) . ?
C13 C14 1.380(3) . ?

C13 H13 0.9300 . ?
C14 C15 1.383(3) . ?
C15 C16 1.385(2) . ?
C15 H15 0.9300 . ?
C16 H16 0.9300 . ?
C51 C56 1.387(2) . ?
C53 C54 1.379(2) . ?
C53 C56 1.393(2) . ?
C53 H53 0.9300 . ?
C54 C55 1.394(2) . ?
C54 H54 0.9300 . ?
C55 H55 0.9300 . ?
C56 H56 0.9300 . ?

loop_
 _geom_angle_atom_site_label_1
 _geom_angle_atom_site_label_2
 _geom_angle_atom_site_label_3
 _geom_angle
 _geom_angle_site_symmetry_1
 _geom_angle_site_symmetry_3
 _geom_angle_publ_flag
C2 N1 C11 127.93(14) . . ?
C2 N1 H1 116.7(16) . . ?
C11 N1 H1 115.3(16) . . ?
C2 N3 N4 119.61(14) . . ?
C2 N3 H3 120.0(14) . . ?
N4 N3 H3 115.0(15) . . ?
C5 N4 N3 119.61(14) . . ?
C5 N4 H4 122.5(15) . . ?
N3 N4 H4 117.6(15) . . ?
C55 N52 C51 116.99(14) . . ?
N1 C2 N3 111.82(14) . . ?
N1 C2 S2 126.49(12) . . ?
N3 C2 S2 121.68(12) . . ?
O5 C5 N4 122.29(15) . . ?
O5 C5 C51 123.01(14) . . ?
N4 C5 C51 114.69(14) . . ?
C16 C11 C12 118.08(14) . . ?
C16 C11 N1 122.55(14) . . ?
C12 C11 N1 119.21(15) . . ?
C13 C12 C11 121.70(15) . . ?
C13 C12 Cl12 118.58(13) . . ?
C11 C12 Cl12 119.72(12) . . ?
C14 C13 C12 118.24(15) . . ?
C14 C13 H13 120.9 . . ?
C12 C13 H13 120.9 . . ?
C13 C14 C15 121.69(15) . . ?
C13 C14 Cl14 118.90(13) . . ?
C15 C14 Cl14 119.40(14) . . ?
C14 C15 C16 119.25(16) . . ?
C14 C15 H15 120.4 . . ?
C16 C15 H15 120.4 . . ?
C15 C16 C11 120.94(16) . . ?
C15 C16 H16 119.5 . . ?
C11 C16 H16 119.5 . . ?
N52 C51 C56 123.88(15) . . ?
N52 C51 C5 116.16(14) . . ?
C56 C51 C5 119.92(14) . . ?
C54 C53 C56 118.96(15) . . ?
C54 C53 H53 120.5 . . ?
C56 C53 H53 120.5 . . ?
C53 C54 C55 118.74(15) . . ?
C53 C54 H54 120.6 . . ?
C55 C54 H54 120.6 . . ?
N52 C55 C54 123.33(16) . . ?
N52 C55 H55 118.3 . . ?
C54 C55 H55 118.3 . . ?
C51 C56 C53 118.09(15) . . ?
C51 C56 H56 121.0 . . ?

C53 C56 H56 121.0 . . ?

```
loop_
 _geom_torsion_atom_site_label_1
 _geom_torsion_atom_site_label_2
 _geom_torsion_atom_site_label_3
 _geom_torsion_atom_site_label_4
 _geom_torsion
 _geom_torsion_site_symmetry_1
 _geom_torsion_site_symmetry_2
 _geom_torsion_site_symmetry_3
 _geom_torsion_site_symmetry_4
 _geom_torsion_publ_flag
C2 N3 N4 C5 -154.75(15) . . . . ?
C11 N1 C2 N3 166.21(15) . . . . ?
C11 N1 C2 S2 -15.1(2) . . . . ?
N4 N3 C2 N1 162.99(14) . . . . ?
N4 N3 C2 S2 -15.7(2) . . . . ?
N3 N4 C5 O5 -2.5(2) . . . . ?
N3 N4 C5 C51 176.16(13) . . . . ?
C2 N1 C11 C16 -38.0(3) . . . . ?
C2 N1 C11 C12 146.60(17) . . . . ?
C16 C11 C12 C13 -2.9(2) . . . . ?
N1 C11 C12 C13 172.78(15) . . . . ?
C16 C11 C12 Cl12 177.64(12) . . . . ?
N1 C11 C12 Cl12 -6.7(2) . . . . ?
C11 C12 C13 C14 0.9(2) . . . . ?
Cl12 C12 C13 C14 -179.55(12) . . . . ?
C12 C13 C14 C15 1.9(3) . . . . ?
C12 C13 C14 Cl14 -177.73(13) . . . . ?
C13 C14 C15 C16 -2.8(3) . . . . ?
Cl14 C14 C15 C16 176.90(13) . . . . ?
C14 C15 C16 C11 0.7(3) . . . . ?
C12 C11 C16 C15 2.0(2) . . . . ?
N1 C11 C16 C15 -173.47(15) . . . . ?
C55 N52 C51 C56 -1.0(2) . . . . ?
C55 N52 C51 C5 -178.49(14) . . . . ?
O5 C5 C51 N52 -177.25(15) . . . . ?
N4 C5 C51 N52 4.1(2) . . . . ?
O5 C5 C51 C56 5.1(2) . . . . ?
N4 C5 C51 C56 -173.48(14) . . . . ?
C56 C53 C54 C55 -0.1(3) . . . . ?
C51 N52 C55 C54 0.4(3) . . . . ?
C53 C54 C55 N52 0.2(3) . . . . ?
N52 C51 C56 C53 1.1(2) . . . . ?
C5 C51 C56 C53 178.49(14) . . . . ?
C54 C53 C56 C51 -0.5(2) . . . . ?

_refine_diff_density_max    0.391
_refine_diff_density_min   -0.415
_refine_diff_density_rms    0.064

_shelx_res_file
;

    shelx.res created by SHELXL-2014/7


TITL mp-mm7 in P-1
CELL  1.54178   7.4539   7.8084  14.3223  104.442   92.188  112.898
ZERR     2.00   0.0002   0.0002   0.0002    0.002    0.002    0.002
LATT   1
SFAC  C    H    CL   N    O    S
UNIT  26   20   4    8    2    2
MERG   2
FMAP   2
ACTA
BOND   $H
CONF
PLAN  -2
```

```
L.S.  40
WGHT    0.052600    0.458500
EXTI    0.006090
FVAR       1.09635
S2    6   -0.049812    0.930236    0.792986    11.00000    0.01719    0.01969 =
        0.01758   -0.00037   -0.00255    0.01044
CL12  3    0.442555    0.635135    0.656755    11.00000    0.01806    0.02740 =
        0.02525    0.00222    0.00428    0.01057
CL14  3   -0.215958    0.055152    0.436919    11.00000    0.04427    0.01986 =
        0.01897   -0.00743   -0.00413    0.00621
O5    5    0.508199    1.211925    1.073057    11.00000    0.02079    0.01875 =
        0.02017   -0.00205   -0.00363    0.01162
N1    4    0.172175    0.724909    0.787858    11.00000    0.01488    0.01736 =
        0.01588   -0.00300   -0.00324    0.00883
H1    2    0.269194    0.723690    0.811538    11.00000   -1.20000
N3    4    0.219693    0.955310    0.928218    11.00000    0.01865    0.01299 =
        0.01564   -0.00201   -0.00159    0.00916
H3    2    0.318225    0.934988    0.945166    11.00000   -1.20000
N4    4    0.222843    1.132086    0.978836    11.00000    0.01667    0.01226 =
        0.01566   -0.00206   -0.00153    0.00835
H4    2    0.135079    1.162054    0.960281    11.00000   -1.20000
N52   4    0.205883    1.466632    1.063023    11.00000    0.02030    0.01568 =
        0.01823   -0.00055   -0.00304    0.00903
C2    1    0.117585    0.864970    0.836494    11.00000    0.01327    0.01351 =
        0.01584    0.00109    0.00111    0.00356
C5    1    0.372713    1.253786    1.048857    11.00000    0.01488    0.01447 =
        0.01303    0.00129    0.00218    0.00634
C11   1    0.074955    0.576946    0.700415    11.00000    0.01970    0.01353 =
        0.01267   -0.00009   -0.00095    0.00758
C12   1    0.186609    0.513691    0.635250    11.00000    0.01737    0.01643 =
        0.01705    0.00253    0.00091    0.00804
C13   1    0.098920    0.354511    0.553451    11.00000    0.03041    0.01813 =
        0.01606    0.00203    0.00464    0.01361
AFIX  43
H13   2    0.174883    0.312753    0.511176    11.00000   -1.20000
AFIX   0
C14   1   -0.103909    0.259914    0.536539    11.00000    0.03080    0.01276 =
        0.01400   -0.00124   -0.00243    0.00544
C15   1   -0.219534    0.323321    0.596990    11.00000    0.02028    0.01780 =
        0.02009    0.00152   -0.00190    0.00223
AFIX  43
H15   2   -0.356188    0.260877    0.582780    11.00000   -1.20000
AFIX   0
C16   1   -0.129540    0.480809    0.678899    11.00000    0.02007    0.01649 =
        0.01694   -0.00012    0.00130    0.00604
AFIX  43
H16   2   -0.206724    0.523012    0.720142    11.00000   -1.20000
AFIX   0
C51   1    0.366436    1.445515    1.093875    11.00000    0.01628    0.01278 =
        0.01258    0.00188    0.00208    0.00640
C53   1    0.514621    1.769333    1.199044    11.00000    0.02088    0.01304 =
        0.01534    0.00030    0.00063    0.00345
AFIX  43
H53   2    0.617269    1.870229    1.244329    11.00000   -1.20000
AFIX   0
C54   1    0.350399    1.793597    1.168940    11.00000    0.02766    0.01335 =
        0.02079   -0.00025    0.00161    0.01064
AFIX  43
H54   2    0.340252    1.910971    1.193580    11.00000   -1.20000
AFIX   0
C55   1    0.199816    1.638807    1.100849    11.00000    0.02494    0.01777 =
        0.02471    0.00070   -0.00399    0.01208
AFIX  43
H55   2    0.089575    1.656175    1.080717    11.00000   -1.20000
AFIX   0
C56   1    0.524376    1.591397    1.160584    11.00000    0.01522    0.01580 =
        0.01471    0.00131    0.00051    0.00564
AFIX  43
H56   2    0.633646    1.570957    1.179092    11.00000   -1.20000
AFIX   0
```

```
HKLF    4

REM  mp-mm7 in P-1
REM R1 =  0.0336 for    2964 Fo > 4sig(Fo)  and  0.0344 for all    3041 data
REM    200 parameters refined using      0 restraints

END

WGHT      0.0515      0.4827

REM Highest difference peak  0.391,  deepest hole -0.415,  1-sigma level  0.064
Q1    1   0.3003  0.5649  0.6430  11.00000  0.05    0.39
Q2    1   0.1562  0.5542  0.6771  11.00000  0.05    0.36
;
```

CIF for 7

```
data_shelx

_audit_creation_method            'SHELXL-2014/7'
_shelx_SHELXL_version_number      '2014/7'
_chemical_name_systematic         ?
_chemical_name_common             ?
_chemical_melting_point           ?
_chemical_formula_moiety          ?
_chemical_formula_sum             'C14 H14 N4 O S2'
_chemical_formula_weight          318.41

loop_
 _atom_type_symbol
 _atom_type_description
 _atom_type_scat_dispersion_real
 _atom_type_scat_dispersion_imag
 _atom_type_scat_source
 'C'  'C'   0.0181   0.0091
 'International Tables Vol C Tables 4.2.6.8 and 6.1.1.4'
 'H'  'H'   0.0000   0.0000
 'International Tables Vol C Tables 4.2.6.8 and 6.1.1.4'
 'N'  'N'   0.0311   0.0180
 'International Tables Vol C Tables 4.2.6.8 and 6.1.1.4'
 'O'  'O'   0.0492   0.0322
 'International Tables Vol C Tables 4.2.6.8 and 6.1.1.4'
 'S'  'S'   0.3331   0.5567
 'International Tables Vol C Tables 4.2.6.8 and 6.1.1.4'

_space_group_crystal_system       monoclinic
_space_group_IT_number            9
_space_group_name_H-M_alt         'C c'
_space_group_name_Hall            'C -2yc'

_shelx_space_group_comment
;
The symmetry employed for this shelxl refinement is uniquely defined
by the following loop, which should always be used as a source of
symmetry information in preference to the above space-group names.
They are only intended as comments.
;

loop_
 _space_group_symop_operation_xyz
 'x, y, z'
 'x, -y, z+1/2'
 'x+1/2, y+1/2, z'
 'x+1/2, -y+1/2, z+1/2'

_cell_length_a                    7.9236(4)
_cell_length_b                    25.2269(2)
_cell_length_c                    7.9636(2)
```

_cell_angle_alpha 90
_cell_angle_beta 113.018(2)
_cell_angle_gamma 90
_cell_volume 1465.09(9)
_cell_formula_units_Z 4
_cell_measurement_temperature 293(2)
_cell_measurement_reflns_used 194
_cell_measurement_theta_min 9.4
_cell_measurement_theta_max 77.4
_exptl_crystal_description needle
_exptl_crystal_colour colourless
_exptl_crystal_density_meas ?
_exptl_crystal_density_method ?
_exptl_crystal_density_diffrn 1.444
_exptl_crystal_F_000 664
_exptl_transmission_factor_min ?
_exptl_transmission_factor_max ?
_exptl_crystal_size_max 0.59
_exptl_crystal_size_mid 0.07
_exptl_crystal_size_min 0.07
_exptl_absorpt_coefficient_mu 3.332
_shelx_estimated_absorpt_T_min ?
_shelx_estimated_absorpt_T_max ?
_exptl_absorpt_correction_type multi-scan
_exptl_absorpt_correction_T_min 0.4190
_exptl_absorpt_correction_T_max 1.0000
_exptl_absorpt_process_details
;
CrysAlisPro 1.171.39.16b (Rigaku Oxford Diffraction, 2015)
Empirical absorption correction using spherical harmonics,
implemented in SCALE3 ABSPACK scaling algorithm.
;
_exptl_absorpt_special_details ?
_diffrn_ambient_temperature 293(2)
_diffrn_radiation_wavelength 1.54178
_diffrn_radiation_type CuK\a
_diffrn_source 'micro-focus sealed X-ray tube'
_diffrn_measurement_device 'four-circle diffractometer'
_diffrn_measurement_device_type 'XtaLAB Synergy, Dualflex, Pilatus 300K'
_diffrn_measurement_method '\w scans'
_diffrn_detector_area_resol_mean ?
_diffrn_reflns_number 7385
_diffrn_reflns_av_unetI/netI 0.0342
_diffrn_reflns_av_R_equivalents 0.0429
_diffrn_reflns_limit_h_min -9
_diffrn_reflns_limit_h_max 9
_diffrn_reflns_limit_k_min -31
_diffrn_reflns_limit_k_max 24
_diffrn_reflns_limit_l_min -9
_diffrn_reflns_limit_l_max 9
_diffrn_reflns_theta_min 6.317
_diffrn_reflns_theta_max 78.939
_diffrn_reflns_theta_full 67.679
_diffrn_measured_fraction_theta_max 0.948
_diffrn_measured_fraction_theta_full 0.999
_diffrn_reflns_Laue_measured_fraction_max 0.948
_diffrn_reflns_Laue_measured_fraction_full 0.999
_diffrn_reflns_point_group_measured_fraction_max 0.830
_diffrn_reflns_point_group_measured_fraction_full 0.904
_reflns_number_total 2622
_reflns_number_gt 2597
_reflns_threshold_expression 'I > 2\s(I)'
_reflns_Friedel_coverage 0.741
_reflns_Friedel_fraction_max 0.710
_reflns_Friedel_fraction_full 0.809

_reflns_special_details
;
Reflections were merged by SHELXL according to the crystal
class for the calculation of statistics and refinement.

```
 _reflns_Friedel_fraction is defined as the number of unique
 Friedel pairs measured divided by the number that would be
 possible theoretically, ignoring centric projections and
 systematic absences.
;

_computing_data_collection        'CrysAlisPro 1.171.39.16b (Rigaku OD, 2015)'
_computing_cell_refinement        'CrysAlisPro 1.171.39.16b (Rigaku OD, 2015)'
_computing_data_reduction         'CrysAlisPro 1.171.39.16b (Rigaku OD, 2015)'
_computing_structure_solution     'SHELXS-2013/1 (Sheldrick, 2014)'
_computing_structure_refinement   'SHELXL-2014/7 (Sheldrick, 2014)'
_computing_molecular_graphics     'ORTEP3 for Windows'
_computing_publication_material   'SHELXL-2014/7 and WINGX'
_refine_special_details           ?
_refine_ls_structure_factor_coef  Fsqd
_refine_ls_matrix_type            full
_refine_ls_weighting_scheme       calc
_refine_ls_weighting_details
'w=1/[\s^2^(Fo^2^)+(0.0829P)^2^+0.4088P] where P=(Fo^2^+2Fc^2^)/3'
_atom_sites_solution_primary      difmap
_atom_sites_solution_secondary    difmap
_atom_sites_solution_hydrogens    difmap
_refine_ls_hydrogen_treatment     mixed
_refine_ls_extinction_method      'SHELXL-2014/7 (Sheldrick 2014'
_refine_ls_extinction_coef        0.0047(6)
_refine_ls_extinction_expression
 'Fc^*^=kFc[1+0.001xFc^2^\l^3^/sin(2\q)]^-1/4^'
_refine_ls_abs_structure_details
;
 Classical Flack method preferred over Parsons because s.u. lower.
;
_refine_ls_abs_structure_Flack    0.00(2)
_chemical_absolute_configuration  ?
_refine_ls_number_reflns          2622
_refine_ls_number_parameters      201
_refine_ls_number_restraints      2
_refine_ls_R_factor_all           0.0395
_refine_ls_R_factor_gt            0.0393
_refine_ls_wR_factor_ref          0.1073
_refine_ls_wR_factor_gt           0.1071
_refine_ls_goodness_of_fit_ref    1.076
_refine_ls_restrained_S_all       1.076
_refine_ls_shift/su_max           0.001
_refine_ls_shift/su_mean          0.000

loop_
 _atom_site_label
 _atom_site_type_symbol
 _atom_site_fract_x
 _atom_site_fract_y
 _atom_site_fract_z
 _atom_site_U_iso_or_equiv
 _atom_site_adp_type
 _atom_site_occupancy
 _atom_site_site_symmetry_order
 _atom_site_calc_flag
 _atom_site_refinement_flags_posn
 _atom_site_refinement_flags_adp
 _atom_site_refinement_flags_occupancy
 _atom_site_disorder_assembly
 _atom_site_disorder_group
S2 S 0.67222(10) 0.90743(3) 0.56405(11) 0.0185(3) Uani 1 1 d . . . . .
S17 S 0.18750(12) 0.72277(3) 0.89163(12) 0.0257(3) Uani 1 1 d . . . . .
O5 O 0.2214(4) 1.01240(9) 0.3897(3) 0.0174(5) Uani 1 1 d . . . . .
N1 N 0.4273(4) 0.93221(12) 0.7115(4) 0.0172(6) Uani 1 1 d . . . . .
H1 H 0.363(6) 0.956(2) 0.737(6) 0.021 Uiso 1 1 d . U . . .
N3 N 0.5672(4) 1.00093(12) 0.6312(4) 0.0168(6) Uani 1 1 d . . . . .
H3 H 0.642(7) 1.0145(19) 0.603(6) 0.020 Uiso 1 1 d . U . . .
N4 N 0.4452(4) 1.03579(12) 0.6595(4) 0.0153(6) Uani 1 1 d . . . . .
```

```
H4 H 0.480(6) 1.0507(18) 0.776(7) 0.018 Uiso 1 1 d . U . . .
N53 N -0.1641(4) 1.06732(13) 0.5854(4) 0.0213(7) Uani 1 1 d . . . . .
C2 C 0.5479(4) 0.94775(14) 0.6423(4) 0.0146(7) Uani 1 1 d . . . . .
C5 C 0.2698(5) 1.03753(13) 0.5326(4) 0.0142(7) Uani 1 1 d . . . . .
C11 C 0.3734(5) 0.88033(14) 0.7403(5) 0.0164(7) Uani 1 1 d . . . . .
C12 C 0.1896(5) 0.87337(14) 0.7088(5) 0.0181(7) Uani 1 1 d . . . . .
H12 H 0.1071 0.9009 0.6588 0.022 Uiso 1 1 calc R U . . .
C13 C 0.1280(5) 0.82571(15) 0.7513(5) 0.0196(7) Uani 1 1 d . . . . .
H13 H 0.0045 0.8214 0.7289 0.024 Uiso 1 1 calc R U . . .
C14 C 0.2501(5) 0.78443(14) 0.8270(5) 0.0194(7) Uani 1 1 d . . . . .
C15 C 0.4360(5) 0.79177(15) 0.8584(5) 0.0227(8) Uani 1 1 d . . . . .
H15 H 0.5189 0.7644 0.9093 0.027 Uiso 1 1 calc R U . . .
C16 C 0.4972(5) 0.83892(14) 0.8150(5) 0.0188(7) Uani 1 1 d . . . . .
H16 H 0.6203 0.8431 0.8354 0.023 Uiso 1 1 calc R U . . .
C18 C -0.0332(6) 0.73571(17) 0.8971(6) 0.0298(9) Uani 1 1 d . . . . .
H81 H -0.1245 0.7365 0.7747 0.036 Uiso 1 1 calc R U . . .
H82 H -0.0308 0.7693 0.9546 0.036 Uiso 1 1 calc R U . . .
H83 H -0.0624 0.7083 0.9649 0.036 Uiso 1 1 calc R U . . .
C51 C 0.1382(5) 1.06838(14) 0.5855(5) 0.0147(7) Uani 1 1 d . . . . .
C52 C -0.0361(5) 1.04683(15) 0.5346(5) 0.0169(7) Uani 1 1 d . . . . .
H52 H -0.0649 1.0167 0.4617 0.020 Uiso 1 1 calc R U . . .
C54 C -0.1220(5) 1.11163(16) 0.6861(5) 0.0232(8) Uani 1 1 d . . . . .
H54 H -0.2097 1.1263 0.7229 0.028 Uiso 1 1 calc R U . . .
C55 C 0.0471(5) 1.13661(16) 0.7378(5) 0.0238(8) Uani 1 1 d . . . . .
H55 H 0.0702 1.1679 0.8046 0.039(14) Uiso 1 1 calc R . . . .
C56 C 0.1803(5) 1.11446(14) 0.6889(5) 0.0196(7) Uani 1 1 d . . . . .
H56 H 0.2955 1.1300 0.7243 0.023 Uiso 1 1 calc R U . . .

loop_
 _atom_site_aniso_label
 _atom_site_aniso_U_11
 _atom_site_aniso_U_22
 _atom_site_aniso_U_33
 _atom_site_aniso_U_23
 _atom_site_aniso_U_13
 _atom_site_aniso_U_12
S2 0.0170(4) 0.0196(4) 0.0212(4) -0.0002(3) 0.0099(3) 0.0052(3)
S17 0.0310(5) 0.0148(4) 0.0293(5) 0.0033(4) 0.0098(4) -0.0028(3)
O5 0.0176(12) 0.0186(11) 0.0166(11) -0.0035(10) 0.0073(9) -0.0018(9)
N1 0.0195(14) 0.0130(14) 0.0230(15) -0.0011(12) 0.0123(12) 0.0018(12)
N3 0.0131(13) 0.0165(15) 0.0252(16) -0.0009(12) 0.0123(12) 0.0007(11)
N4 0.0125(14) 0.0168(14) 0.0182(14) -0.0011(11) 0.0076(11) 0.0008(11)
N53 0.0162(14) 0.0228(16) 0.0263(17) -0.0007(13) 0.0095(12) 0.0004(11)
C2 0.0111(15) 0.0191(17) 0.0119(16) -0.0007(12) 0.0026(12) 0.0008(12)
C5 0.0142(15) 0.0127(15) 0.0166(17) 0.0030(12) 0.0071(13) -0.0016(12)
C11 0.0204(17) 0.0140(16) 0.0151(16) -0.0016(12) 0.0073(13) -0.0007(13)
C12 0.0183(17) 0.0162(17) 0.0185(17) 0.0014(13) 0.0058(13) 0.0013(13)
C13 0.0183(17) 0.0181(17) 0.0221(18) -0.0012(14) 0.0076(14) -0.0031(14)
C14 0.0272(19) 0.0132(16) 0.0160(17) -0.0007(13) 0.0064(14) -0.0038(13)
C15 0.0245(19) 0.0180(17) 0.0233(19) 0.0011(14) 0.0068(15) 0.0048(14)
C16 0.0163(17) 0.0183(17) 0.0209(18) -0.0001(14) 0.0063(14) 0.0025(13)
C18 0.032(2) 0.026(2) 0.032(2) 0.0031(17) 0.0128(18) -0.0082(17)
C51 0.0147(16) 0.0148(15) 0.0163(16) 0.0017(13) 0.0081(12) 0.0006(12)
C52 0.0155(17) 0.0188(17) 0.0159(17) 0.0019(12) 0.0057(13) 0.0009(13)
C54 0.0217(19) 0.0260(19) 0.028(2) 0.0007(16) 0.0161(16) 0.0054(14)
C55 0.0234(18) 0.0203(18) 0.027(2) -0.0043(14) 0.0087(16) 0.0040(14)
C56 0.0175(17) 0.0182(17) 0.0227(18) -0.0028(14) 0.0075(13) -0.0012(13)

_geom_special_details
;
 All esds (except the esd in the dihedral angle between two l.s. planes)
 are estimated using the full covariance matrix.  The cell esds are taken
 into account individually in the estimation of esds in distances, angles
 and torsion angles; correlations between esds in cell parameters are only
 used when they are defined by crystal symmetry.  An approximate (isotropic)
 treatment of cell esds is used for estimating esds involving l.s. planes.
;

loop_
 _geom_bond_atom_site_label_1
```

_geom_bond_atom_site_label_2
_geom_bond_distance
_geom_bond_site_symmetry_2
_geom_bond_publ_flag
S2 C2 1.695(4) . ?
S17 C14 1.769(4) . ?
S17 C18 1.796(5) . ?
O5 C5 1.226(4) . ?
N1 C2 1.335(5) . ?
N1 C11 1.423(5) . ?
N1 H1 0.87(5) . ?
N3 C2 1.357(5) . ?
N3 N4 1.389(4) . ?
N3 H3 0.79(5) . ?
N4 C5 1.362(4) . ?
N4 H4 0.94(5) . ?
N53 C52 1.335(5) . ?
N53 C54 1.340(5) . ?
C5 C51 1.488(5) . ?
C11 C12 1.388(5) . ?
C11 C16 1.397(5) . ?
C12 C13 1.388(5) . ?
C12 H12 0.9300 . ?
C13 C14 1.388(5) . ?
C13 H13 0.9300 . ?
C14 C15 1.406(6) . ?
C15 C16 1.379(5) . ?
C15 H15 0.9300 . ?
C16 H16 0.9300 . ?
C18 H81 0.9600 . ?
C18 H82 0.9600 . ?
C18 H83 0.9600 . ?
C51 C56 1.388(5) . ?
C51 C52 1.389(5) . ?
C52 H52 0.9300 . ?
C54 C55 1.390(6) . ?
C54 H54 0.9300 . ?
C55 C56 1.377(6) . ?
C55 H55 0.9300 . ?
C56 H56 0.9300 . ?

loop_
_geom_angle_atom_site_label_1
_geom_angle_atom_site_label_2
_geom_angle_atom_site_label_3
_geom_angle
_geom_angle_site_symmetry_1
_geom_angle_site_symmetry_3
_geom_angle_publ_flag
C14 S17 C18 103.51(19) . . ?
C2 N1 C11 130.2(3) . . ?
C2 N1 H1 118(3) . . ?
C11 N1 H1 111(3) . . ?
C2 N3 N4 120.8(3) . . ?
C2 N3 H3 124(3) . . ?
N4 N3 H3 115(3) . . ?
C5 N4 N3 118.2(3) . . ?
C5 N4 H4 123(3) . . ?
N3 N4 H4 117(3) . . ?
C52 N53 C54 117.6(3) . . ?
N1 C2 N3 115.8(3) . . ?
N1 C2 S2 126.0(3) . . ?
N3 C2 S2 118.2(3) . . ?
O5 C5 N4 122.0(3) . . ?
O5 C5 C51 122.2(3) . . ?
N4 C5 C51 115.6(3) . . ?
C12 C11 C16 119.7(3) . . ?
C12 C11 N1 116.5(3) . . ?
C16 C11 N1 123.5(3) . . ?
C13 C12 C11 120.6(3) . . ?

C13 C12 H12 119.7 . . ?
C11 C12 H12 119.7 . . ?
C12 C13 C14 120.3(3) . . ?
C12 C13 H13 119.9 . . ?
C14 C13 H13 119.9 . . ?
C13 C14 C15 118.8(3) . . ?
C13 C14 S17 124.0(3) . . ?
C15 C14 S17 117.2(3) . . ?
C16 C15 C14 121.1(3) . . ?
C16 C15 H15 119.5 . . ?
C14 C15 H15 119.5 . . ?
C15 C16 C11 119.6(3) . . ?
C15 C16 H16 120.2 . . ?
C11 C16 H16 120.2 . . ?
S17 C18 H81 109.5 . . ?
S17 C18 H82 109.5 . . ?
H81 C18 H82 109.5 . . ?
S17 C18 H83 109.5 . . ?
H81 C18 H83 109.5 . . ?
H82 C18 H83 109.5 . . ?
C56 C51 C52 118.8(3) . . ?
C56 C51 C5 125.0(3) . . ?
C52 C51 C5 116.1(3) . . ?
N53 C52 C51 123.2(3) . . ?
N53 C52 H52 118.4 . . ?
C51 C52 H52 118.4 . . ?
N53 C54 C55 122.8(3) . . ?
N53 C54 H54 118.6 . . ?
C55 C54 H54 118.6 . . ?
C56 C55 C54 119.3(4) . . ?
C56 C55 H55 120.4 . . ?
C54 C55 H55 120.4 . . ?
C55 C56 C51 118.3(3) . . ?
C55 C56 H56 120.8 . . ?
C51 C56 H56 120.8 . . ?

loop_
_geom_torsion_atom_site_label_1
_geom_torsion_atom_site_label_2
_geom_torsion_atom_site_label_3
_geom_torsion_atom_site_label_4
_geom_torsion
_geom_torsion_site_symmetry_1
_geom_torsion_site_symmetry_2
_geom_torsion_site_symmetry_3
_geom_torsion_site_symmetry_4
_geom_torsion_publ_flag
C2 N3 N4 C5 -69.1(4) . . . . ?
C11 N1 C2 N3 179.4(3) . . . . ?
C11 N1 C2 S2 1.0(5) . . . . ?
N4 N3 C2 N1 -11.0(5) . . . . ?
N4 N3 C2 S2 167.5(3) . . . . ?
N3 N4 C5 O5 -5.5(5) . . . . ?
N3 N4 C5 C51 169.8(3) . . . . ?
C2 N1 C11 C12 -142.7(4) . . . . ?
C2 N1 C11 C16 44.1(5) . . . . ?
C16 C11 C12 C13 0.2(6) . . . . ?
N1 C11 C12 C13 -173.3(3) . . . . ?
C11 C12 C13 C14 0.4(6) . . . . ?
C12 C13 C14 C15 -0.4(5) . . . . ?
C12 C13 C14 S17 178.3(3) . . . . ?
C18 S17 C14 C13 -15.8(4) . . . . ?
C18 S17 C14 C15 163.0(3) . . . . ?
C13 C14 C15 C16 -0.1(6) . . . . ?
S17 C14 C15 C16 -178.9(3) . . . . ?
C14 C15 C16 C11 0.6(6) . . . . ?
C12 C11 C16 C15 -0.7(5) . . . . ?
N1 C11 C16 C15 172.3(4) . . . . ?
O5 C5 C51 C56 -148.9(4) . . . . ?
N4 C5 C51 C56 35.8(5) . . . . ?

O5 C5 C51 C52 35.0(5) . . . . ?
N4 C5 C51 C52 -140.3(3) . . . . ?
C54 N53 C52 C51 1.9(5) . . . . ?
C56 C51 C52 N53 -2.2(5) . . . . ?
C5 C51 C52 N53 174.1(3) . . . . ?
C52 N53 C54 C55 0.3(6) . . . . ?
N53 C54 C55 C56 -2.0(6) . . . . ?
C54 C55 C56 C51 1.5(6) . . . . ?
C52 C51 C56 C55 0.5(5) . . . . ?
C5 C51 C56 C55 -175.5(3) . . . . ?

_refine_diff_density_max   0.277
_refine_diff_density_min   -0.330
_refine_diff_density_rms   0.066

_shelx_res_file
;

```
    shelx.res created by SHELXL-2014/7


TITL mm20 in Cc
CELL  1.54178   7.9236  25.2269   7.9636   90.000  113.018   90.000
ZERR     4.00   0.0004   0.0002   0.0002    0.000    0.002    0.000
LATT  -7
SYMM    X, - Y, 1/2 + Z
SFAC  C    H    N    O    S
UNIT  56   56   16   4    8
MERG   2
FMAP   2
PLAN  -2
ACTA
BOND   $H
CONF
L.S.  40
WGHT    0.082900    0.408800
EXTI    0.004704
FVAR       0.65116
S2    5    0.672223    0.907429    0.564053    11.00000    0.01697    0.01962 =
         0.02121   -0.00022    0.00985    0.00524
S17   5    0.187504    0.722768    0.891634    11.00000    0.03103    0.01482 =
         0.02927    0.00325    0.00976   -0.00276
O5    4    0.221374    1.012404    0.389651    11.00000    0.01758    0.01861 =
         0.01655   -0.00345    0.00728   -0.00184
N1    3    0.427275    0.932214    0.711469    11.00000    0.01948    0.01296 =
         0.02298   -0.00108    0.01228    0.00184
H1    2    0.362811    0.956330    0.736978    11.00000   -1.20000
N3    3    0.567240    1.000926    0.631168    11.00000    0.01312    0.01650 =
         0.02522   -0.00086    0.01234    0.00072
H3    2    0.642449    1.014494    0.602574    11.00000   -1.20000
N4    3    0.445150    1.035786    0.659492    11.00000    0.01250    0.01681 =
         0.01815   -0.00111    0.00756    0.00078
H4    2    0.480349    1.050665    0.776377    11.00000   -1.20000
N53   3   -0.164147    1.067318    0.585358    11.00000    0.01616    0.02277 =
         0.02627   -0.00068    0.00955    0.00039
C2    1    0.547894    0.947746    0.642334    11.00000    0.01109    0.01906 =
         0.01194   -0.00074    0.00255    0.00077
C5    1    0.269832    1.037534    0.532616    11.00000    0.01424    0.01268 =
         0.01664    0.00303    0.00710   -0.00158
C11   1    0.373362    0.880327    0.740316    11.00000    0.02044    0.01405 =
         0.01509   -0.00164    0.00734   -0.00066
C12   1    0.189627    0.873373    0.708785    11.00000    0.01830    0.01619 =
         0.01851    0.00141    0.00583    0.00126
AFIX  43
H12   2    0.107055    0.900893    0.658754    11.00000   -1.20000
AFIX   0
C13   1    0.128037    0.825710    0.751266    11.00000    0.01833    0.01814 =
         0.02213   -0.00124    0.00763   -0.00311
AFIX  43
H13   2    0.004527    0.821416    0.728903    11.00000   -1.20000
```

```
AFIX   0
C14   1    0.250108    0.784432    0.826999    11.00000    0.02718    0.01321 =
        0.01603   -0.00072    0.00639   -0.00377
C15   1    0.435952    0.791771    0.858410    11.00000    0.02452    0.01800 =
        0.02333    0.00113    0.00683    0.00481
AFIX  43
H15   2    0.518870    0.764383    0.909257    11.00000   -1.20000
AFIX   0
C16   1    0.497207    0.838922    0.815026    11.00000    0.01628    0.01829 =
        0.02091   -0.00010    0.00626    0.00252
AFIX  43
H16   2    0.620278    0.843137    0.835385    11.00000   -1.20000
AFIX   0
C18   1   -0.033242    0.735708    0.897079    11.00000    0.03181    0.02632 =
        0.03170    0.00310    0.01277   -0.00824
AFIX 133
H81   2   -0.124517    0.736459    0.774736    11.00000   -1.20000
H82   2   -0.030755    0.769314    0.954558    11.00000   -1.20000
H83   2   -0.062420    0.708265    0.964851    11.00000   -1.20000
AFIX   0
C51   1    0.138216    1.068384    0.585471    11.00000    0.01470    0.01480 =
        0.01632    0.00173    0.00808    0.00060
C52   1   -0.036061    1.046826    0.534615    11.00000    0.01551    0.01877 =
        0.01594    0.00190    0.00572    0.00093
AFIX  43
H52   2   -0.064887    1.016658    0.461688    11.00000   -1.20000
AFIX   0
C54   1   -0.122039    1.111627    0.686055    11.00000    0.02170    0.02601 =
        0.02793    0.00072    0.01611    0.00542
AFIX  43
H54   2   -0.209698    1.126290    0.722917    11.00000   -1.20000
AFIX   0
C55   1    0.047149    1.136612    0.737773    11.00000    0.02336    0.02031 =
        0.02684   -0.00427    0.00875    0.00402
AFIX  43
H55   2    0.070203    1.167931    0.804619    11.00000    0.03901
AFIX   0
C56   1    0.180262    1.114464    0.688907    11.00000    0.01749    0.01820 =
        0.02273   -0.00279    0.00751   -0.00117
AFIX  43
H56   2    0.295453    1.130001    0.724341    11.00000   -1.20000
AFIX   0
HKLF  4

REM  mm20 in Cc
REM R1 =  0.0393 for    2597 Fo > 4sig(Fo)  and  0.0395 for all    2622 data
REM    201 parameters refined using      2 restraints

END

WGHT      0.0844      0.1856

REM Highest difference peak  0.277,  deepest hole -0.330,  1-sigma level  0.066
Q1    1   0.2039  0.7241  1.1592  11.00000  0.05    0.28
Q2    1   0.0972  0.7258  0.9250  11.00000  0.05    0.27
;
```

CIF for 11

```
data_shelx

_audit_creation_method            'SHELXL-2014/7'
_shelx_SHELXL_version_number      '2014/7'
_chemical_name_systematic         ?
_chemical_name_common             ?
_chemical_melting_point           ?
_chemical_formula_moiety          ?
_chemical_formula_sum
 'C13 H10 Cl2 N4 O S'
_chemical_formula_weight          341.21
```

```
loop_
 _atom_type_symbol
 _atom_type_description
 _atom_type_scat_dispersion_real
 _atom_type_scat_dispersion_imag
 _atom_type_scat_source
 'C'  'C'   0.0181   0.0091
 'International Tables Vol C Tables 4.2.6.8 and 6.1.1.4'
 'H'  'H'   0.0000   0.0000
 'International Tables Vol C Tables 4.2.6.8 and 6.1.1.4'
 'Cl'  'Cl'   0.3639   0.7018
 'International Tables Vol C Tables 4.2.6.8 and 6.1.1.4'
 'N'  'N'   0.0311   0.0180
 'International Tables Vol C Tables 4.2.6.8 and 6.1.1.4'
 'O'  'O'   0.0492   0.0322
 'International Tables Vol C Tables 4.2.6.8 and 6.1.1.4'
 'S'  'S'   0.3331   0.5567
 'International Tables Vol C Tables 4.2.6.8 and 6.1.1.4'

_space_group_crystal_system       monoclinic
_space_group_IT_number           14
_space_group_name_H-M_alt         'P 21/c'
_space_group_name_Hall           '-P 2ybc'

_shelx_space_group_comment
;
The symmetry employed for this shelxl refinement is uniquely defined
by the following loop, which should always be used as a source of
symmetry information in preference to the above space-group names.
They are only intended as comments.
;

loop_
 _space_group_symop_operation_xyz
 'x, y, z'
 '-x, y+1/2, -z+1/2'
 '-x, -y, -z'
 'x, -y-1/2, z-1/2'

_cell_length_a                    4.51760(10)
_cell_length_b                    27.8468(4)
_cell_length_c                    11.84240(10)
_cell_angle_alpha                  90
_cell_angle_beta                  98.5730(10)
_cell_angle_gamma                  90
_cell_volume                      1473.14(4)
_cell_formula_units_Z             4
_cell_measurement_temperature     293(2)
_cell_measurement_reflns_used     296
_cell_measurement_theta_min       10.1
_cell_measurement_theta_max       77.4

_exptl_crystal_description        prism
_exptl_crystal_colour            colourless
_exptl_crystal_density_meas       ?
_exptl_crystal_density_method     ?
_exptl_crystal_density_diffrn     1.538
_exptl_crystal_F_000             696
_exptl_transmission_factor_min    ?
_exptl_transmission_factor_max    ?
_exptl_crystal_size_max           0.59
_exptl_crystal_size_mid           0.23
_exptl_crystal_size_min           0.14
_exptl_absorpt_coefficient_mu     5.328
_shelx_estimated_absorpt_T_min    ?
_shelx_estimated_absorpt_T_max    ?
_exptl_absorpt_correction_type    multi-scan
_exptl_absorpt_correction_T_min   0.2818
_exptl_absorpt_correction_T_max   1.0000
```

```
_exptl_absorpt_process_details
;
CrysAlisPro 1.171.39.16b (Rigaku Oxford Diffraction, 2015)
Empirical absorption correction using spherical harmonics,
 implemented in SCALE3 ABSPACK scaling algorithm.
;
_exptl_absorpt_special_details    ?
_diffrn_ambient_temperature       293(2)
_diffrn_radiation_wavelength      1.54178
_diffrn_radiation_type            CuK\a
_diffrn_source                    'micro-focus sealed X-ray tube'
_diffrn_measurement_device        'four-circle diffractometer'
_diffrn_measurement_device_type   'XtaLAB Synergy, Dualflex, Pilatus 300K'
_diffrn_measurement_method        '\w scans'
_diffrn_detector_area_resol_mean  ?
_diffrn_reflns_number             15369
_diffrn_reflns_av_unetI/netI      0.0306
_diffrn_reflns_av_R_equivalents   0.0580
_diffrn_reflns_limit_h_min        -5
_diffrn_reflns_limit_h_max        4
_diffrn_reflns_limit_k_min        -34
_diffrn_reflns_limit_k_max        34
_diffrn_reflns_limit_l_min        -14
_diffrn_reflns_limit_l_max        13
_diffrn_reflns_theta_min          4.095
_diffrn_reflns_theta_max          78.763
_diffrn_reflns_theta_full         67.679
_diffrn_measured_fraction_theta_max   0.953
_diffrn_measured_fraction_theta_full  0.998
_diffrn_reflns_Laue_measured_fraction_max    0.953
_diffrn_reflns_Laue_measured_fraction_full   0.998
_diffrn_reflns_point_group_measured_fraction_max   0.953
_diffrn_reflns_point_group_measured_fraction_full  0.998
_reflns_number_total              3028
_reflns_number_gt                 2761
_reflns_threshold_expression      'I > 2\s(I)'
_reflns_Friedel_coverage          0.000
_reflns_Friedel_fraction_max      .
_reflns_Friedel_fraction_full     .

_reflns_special_details
;
 Reflections were merged by SHELXL according to the crystal
 class for the calculation of statistics and refinement.

 _reflns_Friedel_fraction is defined as the number of unique
 Friedel pairs measured divided by the number that would be
 possible theoretically, ignoring centric projections and
 systematic absences.
;

_computing_data_collection        'CrysAlisPro 1.171.39.16b (Rigaku OD, 2015)'
_computing_cell_refinement        'CrysAlisPro 1.171.39.16b (Rigaku OD, 2015)'
_computing_data_reduction         'CrysAlisPro 1.171.39.16b (Rigaku OD, 2015)'
_computing_structure_solution     'SHELXS-2013/1 (Sheldrick, 2014)'
_computing_structure_refinement   'SHELXL-2014/7 (Sheldrick, 2014)'
_computing_molecular_graphics     'ORTEP3 for Windows'
_computing_publication_material   'SHELXL-2014/7 and WINGX'
_refine_special_details           ?
_refine_ls_structure_factor_coef  Fsqd
_refine_ls_matrix_type            full
_refine_ls_weighting_scheme       calc
_refine_ls_weighting_details
'w=1/[\s^2^(Fo^2^)+(0.0618P)^2^+0.7642P] where P=(Fo^2^+2Fc^2^)/3'
_atom_sites_solution_primary      difmap
_atom_sites_solution_secondary    difmap
_atom_sites_solution_hydrogens    difmap
_refine_ls_hydrogen_treatment     mixed
_refine_ls_extinction_method      'SHELXL-2014/7 (Sheldrick 2014'
_refine_ls_extinction_coef        0.0036(4)
```

_refine_ls_extinction_expression
 'Fc^*^=kFc[1+0.001xFc^2^\l^3^/sin(2\q)]^-1/4^'
_refine_ls_number_reflns 3028
_refine_ls_number_parameters 200
_refine_ls_number_restraints 0
_refine_ls_R_factor_all 0.0429
_refine_ls_R_factor_gt 0.0402
_refine_ls_wR_factor_ref 0.1127
_refine_ls_wR_factor_gt 0.1107
_refine_ls_goodness_of_fit_ref 1.044
_refine_ls_restrained_S_all 1.044
_refine_ls_shift/su_max 0.000
_refine_ls_shift/su_mean 0.000

loop_
 _atom_site_label
 _atom_site_type_symbol
 _atom_site_fract_x
 _atom_site_fract_y
 _atom_site_fract_z
 _atom_site_U_iso_or_equiv
 _atom_site_adp_type
 _atom_site_occupancy
 _atom_site_site_symmetry_order
 _atom_site_calc_flag
 _atom_site_refinement_flags_posn
 _atom_site_refinement_flags_adp
 _atom_site_refinement_flags_occupancy
 _atom_site_disorder_assembly
 _atom_site_disorder_group
S2 S 0.34164(12) 0.33702(2) 0.39834(4) 0.03492(18) Uani 1 1 d . . . . .
Cl12 Cl -0.14348(18) 0.43635(2) 0.01969(4) 0.0549(2) Uani 1 1 d . . . . .
Cl14 Cl -0.4779(2) 0.55803(2) 0.33033(6) 0.0636(2) Uani 1 1 d . . . . .
O5 O 0.4945(3) 0.32404(5) 0.02058(10) 0.0260(3) Uani 1 1 d . . . . .
N1 N -0.0152(4) 0.36895(6) 0.20905(13) 0.0306(4) Uani 1 1 d . . . . .
H1 H -0.076(5) 0.3632(9) 0.142(2) 0.037 Uiso 1 1 d . U . . .
N3 N 0.2130(3) 0.29664(6) 0.19837(12) 0.0243(3) Uani 1 1 d . . . . .
H3 H 0.356(5) 0.2752(8) 0.2195(19) 0.029 Uiso 1 1 d . U . . .
N4 N 0.0855(3) 0.29399(6) 0.08413(12) 0.0230(3) Uani 1 1 d . . . . .
H4 H -0.103(6) 0.2909(8) 0.0689(19) 0.028 Uiso 1 1 d . U . . .
N53 N -0.3109(3) 0.27156(6) -0.25529(13) 0.0276(4) Uani 1 1 d . . . . .
C2 C 0.1704(4) 0.33549(7) 0.26298(15) 0.0238(4) Uani 1 1 d . . . . .
C5 C 0.2383(4) 0.30926(6) 0.00092(14) 0.0184(3) Uani 1 1 d . . . . .
C11 C -0.1028(4) 0.41410(7) 0.24497(16) 0.0286(4) Uani 1 1 d . . . . .
C12 C -0.1829(5) 0.44897(8) 0.16106(17) 0.0359(5) Uani 1 1 d . . . . .
C13 C -0.2942(6) 0.49310(8) 0.18620(19) 0.0453(6) Uani 1 1 d . . . . .
H13 H -0.3470 0.5158 0.1291 0.054 Uiso 1 1 calc R U . . .
C14 C -0.3257(6) 0.50293(8) 0.2982(2) 0.0400(5) Uani 1 1 d . . . . .
C15 C -0.2447(5) 0.46995(8) 0.38367(18) 0.0377(5) Uani 1 1 d . . . . .
H15 H -0.2637 0.4774 0.4588 0.045 Uiso 1 1 calc R U . . .
C16 C -0.1348(5) 0.42562(8) 0.35706(17) 0.0352(5) Uani 1 1 d . . . . .
H16 H -0.0816 0.4032 0.4148 0.042 Uiso 1 1 calc R U . . .
C51 C 0.0679(4) 0.30708(6) -0.11721(14) 0.0196(3) Uani 1 1 d . . . . .
C52 C -0.1529(4) 0.27286(6) -0.15030(14) 0.0216(4) Uani 1 1 d . . . . .
H52 H -0.1922 0.2500 -0.0973 0.026 Uiso 1 1 calc R U . . .
C54 C -0.2472(4) 0.30438(8) -0.33077(15) 0.0316(4) Uani 1 1 d . . . . .
H54 H -0.3578 0.3040 -0.4036 0.038 Uiso 1 1 calc R U . . .
C55 C -0.0268(5) 0.33871(7) -0.30657(16) 0.0324(5) Uani 1 1 d . . . . .
H55 H 0.0126 0.3604 -0.3622 0.039 Uiso 1 1 calc R U . . .
C56 C 0.1349(4) 0.34018(7) -0.19737(16) 0.0266(4) Uani 1 1 d . . . . .
H56 H 0.2851 0.3629 -0.1783 0.032 Uiso 1 1 calc R U . . .

loop_
 _atom_site_aniso_label
 _atom_site_aniso_U_11
 _atom_site_aniso_U_22
 _atom_site_aniso_U_33
 _atom_site_aniso_U_23
 _atom_site_aniso_U_13
 _atom_site_aniso_U_12

S2 0.0398(3) 0.0417(3) 0.0184(3) 0.00103(18) -0.0114(2) -0.0016(2)
Cl12 0.1029(6) 0.0403(3) 0.0240(3) 0.0087(2) 0.0175(3) 0.0155(3)
Cl14 0.1097(6) 0.0272(3) 0.0644(4) -0.0001(3) 0.0478(4) 0.0003(3)
O5 0.0165(6) 0.0378(7) 0.0227(6) -0.0013(5) -0.0007(5) -0.0032(5)
N1 0.0350(9) 0.0395(9) 0.0148(7) 0.0003(6) -0.0043(6) 0.0063(7)
N3 0.0208(7) 0.0351(9) 0.0146(7) 0.0042(6) -0.0052(6) 0.0007(6)
N4 0.0155(7) 0.0358(8) 0.0155(7) 0.0037(6) -0.0045(5) -0.0033(6)
N53 0.0274(8) 0.0318(8) 0.0206(7) -0.0073(6) -0.0063(6) 0.0068(6)
C2 0.0208(9) 0.0311(9) 0.0185(8) 0.0051(7) -0.0001(7) -0.0064(7)
C5 0.0174(8) 0.0180(8) 0.0189(8) -0.0003(6) -0.0001(6) 0.0040(6)
C11 0.0287(10) 0.0329(10) 0.0234(9) 0.0011(7) 0.0012(7) -0.0030(7)
C12 0.0505(13) 0.0336(11) 0.0248(10) 0.0018(8) 0.0099(9) -0.0022(9)
C13 0.0714(17) 0.0302(11) 0.0373(12) 0.0081(9) 0.0179(11) -0.0001(10)
C14 0.0536(13) 0.0263(10) 0.0445(12) -0.0025(9) 0.0220(10) -0.0077(9)
C15 0.0463(12) 0.0391(11) 0.0300(10) -0.0059(9) 0.0132(9) -0.0084(9)
C16 0.0405(11) 0.0416(11) 0.0232(10) 0.0022(8) 0.0040(8) -0.0010(9)
C51 0.0173(8) 0.0237(8) 0.0170(8) -0.0004(6) 0.0003(6) 0.0062(6)
C52 0.0203(8) 0.0242(8) 0.0189(8) -0.0026(6) -0.0020(6) 0.0053(6)
C54 0.0349(10) 0.0407(11) 0.0161(8) -0.0024(7) -0.0067(7) 0.0144(8)
C55 0.0416(11) 0.0367(11) 0.0188(9) 0.0074(7) 0.0040(8) 0.0102(8)
C56 0.0275(9) 0.0293(9) 0.0229(9) 0.0027(7) 0.0032(7) 0.0023(7)

_geom_special_details
;
 All esds (except the esd in the dihedral angle between two l.s. planes)
 are estimated using the full covariance matrix. The cell esds are taken
 into account individually in the estimation of esds in distances, angles
 and torsion angles; correlations between esds in cell parameters are only
 used when they are defined by crystal symmetry. An approximate (isotropic)
 treatment of cell esds is used for estimating esds involving l.s. planes.
;

loop_
 _geom_bond_atom_site_label_1
 _geom_bond_atom_site_label_2
 _geom_bond_distance
 _geom_bond_site_symmetry_2
 _geom_bond_publ_flag
S2 C2 1.6733(17) . ?
Cl12 C12 1.745(2) . ?
Cl14 C14 1.747(2) . ?
O5 C5 1.217(2) . ?
N1 C2 1.349(2) . ?
N1 C11 1.403(3) . ?
N3 C2 1.355(2) . ?
N3 N4 1.3911(19) . ?
N4 C5 1.353(2) . ?
N53 C54 1.339(3) . ?
N53 C52 1.339(2) . ?
C5 C51 1.494(2) . ?
C11 C16 1.394(3) . ?
C11 C12 1.398(3) . ?
C12 C13 1.377(3) . ?
C13 C14 1.382(3) . ?
C14 C15 1.376(3) . ?
C15 C16 1.384(3) . ?
C51 C56 1.388(3) . ?
C51 C52 1.393(2) . ?
C54 C55 1.379(3) . ?
C55 C56 1.388(3) . ?

loop_
 _geom_angle_atom_site_label_1
 _geom_angle_atom_site_label_2
 _geom_angle_atom_site_label_3
 _geom_angle
 _geom_angle_site_symmetry_1
 _geom_angle_site_symmetry_3
 _geom_angle_publ_flag
C2 N1 C11 131.22(16) . . ?

C2 N3 N4 121.22(15) . . ?
C5 N4 N3 120.62(14) . . ?
C54 N53 C52 117.63(16) . . ?
N1 C2 N3 114.50(15) . . ?
N1 C2 S2 127.01(15) . . ?
N3 C2 S2 118.48(13) . . ?
O5 C5 N4 122.71(15) . . ?
O5 C5 C51 122.36(15) . . ?
N4 C5 C51 114.92(14) . . ?
C16 C11 C12 117.68(19) . . ?
C16 C11 N1 124.67(18) . . ?
C12 C11 N1 117.50(17) . . ?
C13 C12 C11 122.03(19) . . ?
C13 C12 Cl12 118.67(17) . . ?
C11 C12 Cl12 119.30(17) . . ?
C12 C13 C14 118.5(2) . . ?
C15 C14 C13 121.3(2) . . ?
C15 C14 Cl14 119.74(17) . . ?
C13 C14 Cl14 118.92(18) . . ?
C14 C15 C16 119.53(19) . . ?
C15 C16 C11 120.9(2) . . ?
C56 C51 C52 118.80(15) . . ?
C56 C51 C5 118.60(16) . . ?
C52 C51 C5 122.59(15) . . ?
N53 C52 C51 122.69(17) . . ?
N53 C54 C55 123.67(16) . . ?
C54 C55 C56 118.53(18) . . ?
C55 C56 C51 118.64(18) . . ?

loop_
 _geom_torsion_atom_site_label_1
 _geom_torsion_atom_site_label_2
 _geom_torsion_atom_site_label_3
 _geom_torsion_atom_site_label_4
 _geom_torsion
 _geom_torsion_site_symmetry_1
 _geom_torsion_site_symmetry_2
 _geom_torsion_site_symmetry_3
 _geom_torsion_site_symmetry_4
 _geom_torsion_publ_flag
C2 N3 N4 C5 92.1(2) . . . . ?
C11 N1 C2 N3 -176.89(19) . . . . ?
C11 N1 C2 S2 4.4(3) . . . . ?
N4 N3 C2 N1 3.5(2) . . . . ?
N4 N3 C2 S2 -177.69(12) . . . . ?
N3 N4 C5 O5 3.0(3) . . . . ?
N3 N4 C5 C51 -176.47(15) . . . . ?
C2 N1 C11 C16 -32.6(3) . . . . ?
C2 N1 C11 C12 151.9(2) . . . . ?
C16 C11 C12 C13 -1.0(3) . . . . ?
N1 C11 C12 C13 174.8(2) . . . . ?
C16 C11 C12 Cl12 179.45(16) . . . . ?
N1 C11 C12 Cl12 -4.8(3) . . . . ?
C11 C12 C13 C14 0.3(4) . . . . ?
Cl12 C12 C13 C14 179.86(19) . . . . ?
C12 C13 C14 C15 0.9(4) . . . . ?
C12 C13 C14 Cl14 -177.97(19) . . . . ?
C13 C14 C15 C16 -1.3(4) . . . . ?
Cl14 C14 C15 C16 177.56(18) . . . . ?
C14 C15 C16 C11 0.5(3) . . . . ?
C12 C11 C16 C15 0.6(3) . . . . ?
N1 C11 C16 C15 -174.9(2) . . . . ?
O5 C5 C51 C56 -29.1(2) . . . . ?
N4 C5 C51 C56 150.30(16) . . . . ?
O5 C5 C51 C52 150.14(17) . . . . ?
N4 C5 C51 C52 -30.4(2) . . . . ?
C54 N53 C52 C51 1.0(3) . . . . ?
C56 C51 C52 N53 -2.4(3) . . . . ?
C5 C51 C52 N53 178.36(15) . . . . ?
C52 N53 C54 C55 0.9(3) . . . . ?

N53 C54 C55 C56 -1.4(3) . . . . ?
C54 C55 C56 C51 0.0(3) . . . . ?
C52 C51 C56 C55 1.8(3) . . . . ?
C5 C51 C56 C55 -178.92(16) . . . . ?

_refine_diff_density_max   0.400
_refine_diff_density_min   -0.407
_refine_diff_density_rms   0.061

_shelx_res_file
;

    shelx.res created by SHELXL-2014/7


```
TITL mm28 in P21/c
CELL  1.54178   4.5176  27.8468  11.8424   90.000   98.573   90.000
ZERR     4.00  0.0001   0.0004   0.0001    0.000    0.001    0.000
LATT   1
SYMM  - X, 1/2 + Y, 1/2 - Z
SFAC  C    H    CL   N    O    S
UNIT  52   40   8    16   4    4
MERG   2
BOND  &H
CONF
FMAP   2
PLAN  -2
ACTA
L.S.  40
WGHT    0.061800    0.764200
EXTI    0.003596
FVAR       0.60458
S2    6    0.341639    0.337020    0.398338    11.00000    0.03984    0.04174 =
        0.01836    0.00103   -0.01143   -0.00164
CL12  3   -0.143476    0.436353    0.019690    11.00000    0.10288    0.04028 =
        0.02399    0.00869    0.01752    0.01551
CL14  3   -0.477875    0.558034    0.330334    11.00000    0.10966    0.02725 =
        0.06442   -0.00005    0.04780    0.00026
O5    5    0.494454    0.324042    0.020582    11.00000    0.01647    0.03780 =
        0.02274   -0.00132   -0.00069   -0.00321
N1    4   -0.015243    0.368954    0.209048    11.00000    0.03499    0.03952 =
        0.01476    0.00033   -0.00427    0.00630
H1    2   -0.075819    0.363186    0.141838    11.00000   -1.20000
N3    4    0.213004    0.296638    0.198371    11.00000    0.02084    0.03505 =
        0.01465    0.00416   -0.00525    0.00071
H3    2    0.355800    0.275182    0.219480    11.00000   -1.20000
N4    4    0.085536    0.293989    0.084129    11.00000    0.01550    0.03581 =
        0.01549    0.00368   -0.00453   -0.00327
H4    2   -0.102579    0.290894    0.068919    11.00000   -1.20000
N53   4   -0.310866    0.271561   -0.255285    11.00000    0.02738    0.03175 =
        0.02060   -0.00727   -0.00626    0.00676
C2    1    0.170444    0.335491    0.262978    11.00000    0.02083    0.03111 =
        0.01845    0.00511   -0.00008   -0.00643
C5    1    0.238334    0.309258    0.000922    11.00000    0.01744    0.01798 =
        0.01893   -0.00031   -0.00015    0.00404
C11   1   -0.102783    0.414103    0.244969    11.00000    0.02870    0.03286 =
        0.02340    0.00107    0.00120   -0.00298
C12   1   -0.182867    0.448970    0.161059    11.00000    0.05053    0.03361 =
        0.02482    0.00178    0.00994   -0.00225
C13   1   -0.294208    0.493098    0.186203    11.00000    0.07137    0.03021 =
        0.03735    0.00812    0.01788   -0.00008
AFIX  43
H13   2   -0.347015    0.515778    0.129106    11.00000   -1.20000
AFIX   0
C14   1   -0.325717    0.502927    0.298161    11.00000    0.05355    0.02635 =
        0.04454   -0.00253    0.02202   -0.00767
C15   1   -0.244736    0.469948    0.383667    11.00000    0.04632    0.03907 =
        0.02998   -0.00586    0.01323   -0.00842
AFIX  43
H15   2   -0.263692    0.477379    0.458817    11.00000   -1.20000
```

```
AFIX   0
C16   1   -0.134825    0.425619    0.357062    11.00000    0.04049    0.04159 =
        0.02319    0.00216    0.00397   -0.00098
AFIX  43
H16   2   -0.081634    0.403214    0.414759    11.00000   -1.20000
AFIX   0
C51   1    0.067886    0.307085   -0.117206    11.00000    0.01729    0.02372 =
        0.01699   -0.00040    0.00035    0.00619
C52   1   -0.152935    0.272862   -0.150300    11.00000    0.02034    0.02420 =
        0.01890   -0.00259   -0.00199    0.00528
AFIX  43
H52   2   -0.192239    0.249982   -0.097283    11.00000   -1.20000
AFIX   0
C54   1   -0.247208    0.304383   -0.330767    11.00000    0.03492    0.04065 =
        0.01610   -0.00236   -0.00671    0.01435
AFIX  43
H54   2   -0.357833    0.304008   -0.403579    11.00000   -1.20000
AFIX   0
C55   1   -0.026802    0.338706   -0.306570    11.00000    0.04160    0.03666 =
        0.01881    0.00736    0.00402    0.01016
AFIX  43
H55   2    0.012588    0.360383   -0.362195    11.00000   -1.20000
AFIX   0
C56   1    0.134914    0.340180   -0.197366    11.00000    0.02750    0.02927 =
        0.02295    0.00270    0.00322    0.00234
AFIX  43
H56   2    0.285095    0.362870   -0.178272    11.00000   -1.20000
AFIX   0
HKLF    4

REM  mm28 in P21/c
REM R1 =  0.0402 for    2761 Fo > 4sig(Fo)  and  0.0429 for all    3028 data
REM    200 parameters refined using      0 restraints

END

WGHT      0.0610      0.7552

REM Highest difference peak  0.400,  deepest hole -0.407,  1-sigma level  0.061
Q1    1  -0.2687  0.5580  0.3405  11.00000  0.05    0.40
Q2    1   0.1096  0.3341  0.4070  11.00000  0.05    0.32
;
```

CIF for 13

```
data_shelx

_audit_creation_method            'SHELXL-2014/7'
_shelx_SHELXL_version_number      '2014/7'
_chemical_name_systematic         ?
_chemical_name_common             ?
_chemical_melting_point           ?
_chemical_formula_moiety          '2(C13 H11 F N4 O S), C3 H8 O'
_chemical_formula_sum             'C29 H30 F2 N8 O3 S2'
_chemical_formula_weight          640.72

loop_
 _atom_type_symbol
 _atom_type_description
 _atom_type_scat_dispersion_real
 _atom_type_scat_dispersion_imag
 _atom_type_scat_source
 'C'  'C'   0.0181   0.0091
 'International Tables Vol C Tables 4.2.6.8 and 6.1.1.4'
 'H'  'H'   0.0000   0.0000
 'International Tables Vol C Tables 4.2.6.8 and 6.1.1.4'
 'F'  'F'   0.0727   0.0534
 'International Tables Vol C Tables 4.2.6.8 and 6.1.1.4'
 'N'  'N'   0.0311   0.0180
 'International Tables Vol C Tables 4.2.6.8 and 6.1.1.4'
 'O'  'O'   0.0492   0.0322
```

'International Tables Vol C Tables 4.2.6.8 and 6.1.1.4'
'S' 'S' 0.3331 0.5567
'International Tables Vol C Tables 4.2.6.8 and 6.1.1.4'

_space_group_crystal_system monoclinic
_space_group_IT_number 14
_space_group_name_H-M_alt 'P 21/n'
_space_group_name_Hall '-P 2yn'

_shelx_space_group_comment
;
The symmetry employed for this shelxl refinement is uniquely defined
by the following loop, which should always be used as a source of
symmetry information in preference to the above space-group names.
They are only intended as comments.
;

loop_
_space_group_symop_operation_xyz
'x, y, z'
'-x+1/2, y+1/2, -z+1/2'
'-x, -y, -z'
'x-1/2, -y-1/2, z-1/2'

_cell_length_a 13.4658(2)
_cell_length_b 9.80280(10)
_cell_length_c 23.9666(3)
_cell_angle_alpha 90
_cell_angle_beta 104.4020(10)
_cell_angle_gamma 90
_cell_volume 3064.23(7)
_cell_formula_units_Z 4
_cell_measurement_temperature 293(2)
_cell_measurement_reflns_used 472
_cell_measurement_theta_min 3.8
_cell_measurement_theta_max 77.4

_exptl_crystal_description prism
_exptl_crystal_colour colourless
_exptl_crystal_density_meas ?
_exptl_crystal_density_method ?
_exptl_crystal_density_diffrn 1.389
_exptl_crystal_F_000 1336
_exptl_transmission_factor_min ?
_exptl_transmission_factor_max ?
_exptl_crystal_size_max 0.58
_exptl_crystal_size_mid 0.27
_exptl_crystal_size_min 0.06
_exptl_absorpt_coefficient_mu 2.070
_shelx_estimated_absorpt_T_min ?
_shelx_estimated_absorpt_T_max ?
_exptl_absorpt_correction_type multi-scan
_exptl_absorpt_correction_T_min 0.349
_exptl_absorpt_correction_T_max 1.000
_exptl_absorpt_process_details
;
CrysAlisPro 1.171.39.16b (Rigaku Oxford Diffraction, 2015)
Empirical absorption correction using spherical harmonics,
implemented in SCALE3 ABSPACK scaling algorithm.
;
_exptl_absorpt_special_details ?
_diffrn_ambient_temperature 293(2)
_diffrn_radiation_wavelength 1.54184
_diffrn_radiation_type CuK\a
_diffrn_source 'micro-focus sealed X-ray tube'
_diffrn_measurement_device 'four-circle diffractometer'
_diffrn_measurement_device_type 'XtaLAB Synergy, Dualflex, Pilatus 300K'
_diffrn_measurement_method '\w scans'
_diffrn_detector_area_resol_mean ?
_diffrn_reflns_number 32030

```
_diffrn_reflns_av_unetI/netI      0.0344
_diffrn_reflns_av_R_equivalents   0.0511
_diffrn_reflns_limit_h_min        -17
_diffrn_reflns_limit_h_max        13
_diffrn_reflns_limit_k_min        -11
_diffrn_reflns_limit_k_max        12
_diffrn_reflns_limit_l_min        -28
_diffrn_reflns_limit_l_max        30
_diffrn_reflns_theta_min          3.808
_diffrn_reflns_theta_max          78.856
_diffrn_reflns_theta_full         67.684
_diffrn_measured_fraction_theta_max   0.955
_diffrn_measured_fraction_theta_full  1.000
_diffrn_reflns_Laue_measured_fraction_max    0.955
_diffrn_reflns_Laue_measured_fraction_full   1.000
_diffrn_reflns_point_group_measured_fraction_max   0.955
_diffrn_reflns_point_group_measured_fraction_full  1.000
_reflns_number_total              6328
_reflns_number_gt                 5725
_reflns_threshold_expression      'I > 2\s(I)'
_reflns_Friedel_coverage          0.000
_reflns_Friedel_fraction_max      .
_reflns_Friedel_fraction_full     .

_reflns_special_details
;
 Reflections were merged by SHELXL according to the crystal
 class for the calculation of statistics and refinement.

 _reflns_Friedel_fraction is defined as the number of unique
 Friedel pairs measured divided by the number that would be
 possible theoretically, ignoring centric projections and
 systematic absences.
;

_computing_data_collection        'CrysAlisPro 1.171.39.16b (Rigaku OD, 2015)'
_computing_cell_refinement        'CrysAlisPro 1.171.39.16b (Rigaku OD, 2015)'
_computing_data_reduction         'CrysAlisPro 1.171.39.16b (Rigaku OD, 2015)'
_computing_structure_solution     'SHELXS-2013/1 (Sheldrick, 2014)'
_computing_structure_refinement   'SHELXL-2014/7 (Sheldrick, 2014)'
_computing_molecular_graphics     'ORTEP3 for Windows'
_computing_publication_material   'SHELXL-2014/7 and WINGX'
_refine_special_details           ?
_refine_ls_structure_factor_coef  Fsqd
_refine_ls_matrix_type            full
_refine_ls_weighting_scheme       calc
_refine_ls_weighting_details
'w=1/[\s^2^(Fo^2^)+(0.0880P)^2^+3.7922P] where P=(Fo^2^+2Fc^2^)/3'
_atom_sites_solution_primary      difmap
_atom_sites_solution_secondary    difmap
_atom_sites_solution_hydrogens    mixed
_refine_ls_hydrogen_treatment     mixed
_refine_ls_extinction_method      none
_refine_ls_extinction_coef        .
_refine_ls_number_reflns          6328
_refine_ls_number_parameters      415
_refine_ls_number_restraints      0
_refine_ls_R_factor_all           0.0605
_refine_ls_R_factor_gt            0.0567
_refine_ls_wR_factor_ref          0.1700
_refine_ls_wR_factor_gt           0.1664
_refine_ls_goodness_of_fit_ref    1.062
_refine_ls_restrained_S_all       1.062
_refine_ls_shift/su_max           0.000
_refine_ls_shift/su_mean          0.000

loop_
 _atom_site_label
 _atom_site_type_symbol
 _atom_site_fract_x
```

_atom_site_fract_y
_atom_site_fract_z
_atom_site_U_iso_or_equiv
_atom_site_adp_type
_atom_site_occupancy
_atom_site_site_symmetry_order
_atom_site_calc_flag
_atom_site_refinement_flags_posn
_atom_site_refinement_flags_adp
_atom_site_refinement_flags_occupancy
_atom_site_disorder_assembly
_atom_site_disorder_group
S2A S 0.02792(4) 0.22697(6) 0.34391(2) 0.02327(16) Uani 1 1 d . . . . .
O5A O -0.25872(12) 0.09498(17) 0.21853(8) 0.0280(4) Uani 1 1 d . . . . .
N1A N -0.16376(14) 0.2550(2) 0.35567(8) 0.0259(4) Uani 1 1 d . . . . .
H1A H -0.230(3) 0.268(3) 0.3393(14) 0.039 Uiso 1 1 d . U . . .
N3A N -0.13694(13) 0.3035(2) 0.26689(9) 0.0226(4) Uani 1 1 d . . . . .
H3A H -0.102(2) 0.298(3) 0.2426(13) 0.034 Uiso 1 1 d . U . . .
N4A N -0.24200(13) 0.3172(2) 0.24376(8) 0.0208(4) Uani 1 1 d . . . . .
H4A H -0.263(2) 0.391(3) 0.2455(13) 0.031 Uiso 1 1 d . U . . .
N54A N -0.62407(14) 0.2539(2) 0.16129(8) 0.0247(4) Uani 1 1 d . . . . .
F12A F -0.21064(13) -0.00912(19) 0.37139(8) 0.0478(4) Uani 1 1 d . . . . .
C2A C -0.09919(16) 0.2615(2) 0.32169(10) 0.0211(4) Uani 1 1 d . . . . .
C5A C -0.29735(16) 0.2081(2) 0.21913(9) 0.0211(4) Uani 1 1 d . . . . .
C11A C -0.13729(17) 0.1987(3) 0.41203(10) 0.0313(6) Uani 1 1 d . . . . .
C12A C -0.16399(19) 0.0631(3) 0.41910(12) 0.0384(7) Uani 1 1 d . . . . .
C13A C -0.1451(2) -0.0010(4) 0.47197(13) 0.0507(8) Uani 1 1 d . . . . .
H13A H -0.1625 -0.0920 0.4751 0.076 Uiso 1 1 calc R U . . .
C14A C -0.1002(2) 0.0732(5) 0.51920(14) 0.0560(10) Uani 1 1 d . . . . .
H14A H -0.0879 0.0321 0.5553 0.084 Uiso 1 1 calc R U . . .
C15A C -0.0728(2) 0.2057(5) 0.51559(13) 0.0599(11) Uani 1 1 d . . . . .
H15A H -0.0418 0.2533 0.5489 0.090 Uiso 1 1 calc R U . . .
C16A C -0.0913(2) 0.2729(4) 0.46047(12) 0.0484(8) Uani 1 1 d . . . . .
H16A H -0.0728 0.3635 0.4577 0.073 Uiso 1 1 calc R U . . .
C51A C -0.41037(16) 0.2318(2) 0.19498(9) 0.0204(4) Uani 1 1 d . . . . .
C52A C -0.47203(17) 0.1174(2) 0.17911(10) 0.0252(5) Uani 1 1 d . . . . .
H52A H -0.4428 0.0314 0.1793 0.038 Uiso 1 1 calc R U . . .
C53A C -0.57728(17) 0.1330(3) 0.16313(10) 0.0257(5) Uani 1 1 d . . . . .
H53A H -0.6177 0.0555 0.1531 0.039 Uiso 1 1 calc R U . . .
C55A C -0.56422(17) 0.3643(3) 0.17394(10) 0.0255(5) Uani 1 1 d . . . . .
H55A H -0.5956 0.4494 0.1712 0.038 Uiso 1 1 calc R U . . .
C56A C -0.45774(16) 0.3589(2) 0.19089(10) 0.0228(4) Uani 1 1 d . . . . .
H56A H -0.4191 0.4383 0.1993 0.034 Uiso 1 1 calc R U . . .
S2B S -0.03538(4) 0.31222(6) 0.15516(2) 0.02333(16) Uani 1 1 d . . . . .
O5B O 0.25456(11) 0.45279(17) 0.27855(7) 0.0274(4) Uani 1 1 d . . . . .
N1B N 0.15703(14) 0.2906(2) 0.14422(8) 0.0238(4) Uani 1 1 d . . . . .
H1B H 0.218(3) 0.278(3) 0.1573(14) 0.036 Uiso 1 1 d . U . . .
N3B N 0.13089(14) 0.2435(2) 0.23314(8) 0.0223(4) Uani 1 1 d . . . . .
H3B H 0.097(2) 0.248(3) 0.2572(13) 0.034 Uiso 1 1 d . U . . .
N4B N 0.23574(13) 0.2292(2) 0.25640(8) 0.0211(4) Uani 1 1 d . . . . .
H4B H 0.259(2) 0.150(3) 0.2554(13) 0.032 Uiso 1 1 d . U . . .
N54B N 0.61830(14) 0.2909(2) 0.33900(8) 0.0244(4) Uani 1 1 d . . . . .
F12B F 0.21586(12) 0.54232(17) 0.11768(7) 0.0427(4) Uani 1 1 d . . . . .
C2B C 0.09199(16) 0.2831(2) 0.17793(10) 0.0206(4) Uani 1 1 d . . . . .
C5B C 0.29224(16) 0.3388(2) 0.27945(9) 0.0208(4) Uani 1 1 d . . . . .
C11B C 0.13044(16) 0.3358(3) 0.08611(10) 0.0257(5) Uani 1 1 d . . . . .
C12B C 0.16433(18) 0.4630(3) 0.07316(11) 0.0299(5) Uani 1 1 d . . . . .
C13B C 0.1480(2) 0.5108(3) 0.01760(12) 0.0380(6) Uani 1 1 d . . . . .
H13B H 0.1712 0.5966 0.0102 0.057 Uiso 1 1 calc R U . . .
C14B C 0.0965(2) 0.4284(3) -0.02660(12) 0.0394(7) Uani 1 1 d . . . . .
H14B H 0.0851 0.4588 -0.0644 0.059 Uiso 1 1 calc R U . . .
C15B C 0.0616(2) 0.3015(3) -0.01571(12) 0.0398(7) Uani 1 1 d . . . . .
H15B H 0.0270 0.2469 -0.0461 0.060 Uiso 1 1 calc R U . . .
C16B C 0.07808(19) 0.2546(3) 0.04102(11) 0.0321(5) Uani 1 1 d . . . . .
H16B H 0.0540 0.1693 0.0484 0.048 Uiso 1 1 calc R U . . .
C51B C 0.40463(16) 0.3138(2) 0.30439(9) 0.0201(4) Uani 1 1 d . . . . .
C52B C 0.46650(17) 0.4281(2) 0.32068(10) 0.0260(5) Uani 1 1 d . . . . .
H52B H 0.4373 0.5142 0.3204 0.039 Uiso 1 1 calc R U . . .
C53B C 0.57193(17) 0.4122(3) 0.33731(10) 0.0265(5) Uani 1 1 d . . . . .
H53B H 0.6124 0.4893 0.3478 0.040 Uiso 1 1 calc R U . . .

C55B C 0.55827(17) 0.1811(2) 0.32581(10) 0.0247(5) Uani 1 1 d . . . . .
H55B H 0.5895 0.0959 0.3284 0.037 Uiso 1 1 calc R U . . .
C56B C 0.45197(16) 0.1866(2) 0.30845(10) 0.0223(4) Uani 1 1 d . . . . .
H56B H 0.4133 0.1072 0.2998 0.033 Uiso 1 1 calc R U . . .
O2 O 0.16915(19) 0.2960(3) 0.47766(10) 0.0724(9) Uani 1 1 d . . . . .
H1 H 0.1292 0.2774 0.4468 0.109 Uiso 1 1 calc R U . . .
C1 C 0.2862(4) 0.4547(5) 0.53103(17) 0.0748(12) Uani 1 1 d . . . . .
H11 H 0.3230 0.5381 0.5299 0.112 Uiso 1 1 calc R U . . .
H12 H 0.2429 0.4646 0.5571 0.112 Uiso 1 1 calc R U . . .
H13 H 0.3340 0.3818 0.5439 0.112 Uiso 1 1 calc R U . . .
C2 C 0.2219(3) 0.4230(4) 0.47215(15) 0.0570(9) Uani 1 1 d . . . . .
H21 H 0.1718 0.4960 0.4592 0.085 Uiso 1 1 calc R U . . .
C3 C 0.2830(3) 0.4011(5) 0.42876(18) 0.0662(10) Uani 1 1 d . . . . .
H31 H 0.3187 0.4836 0.4243 0.099 Uiso 1 1 calc R U . . .
H32 H 0.3318 0.3292 0.4416 0.099 Uiso 1 1 calc R U . . .
H33 H 0.2379 0.3764 0.3925 0.099 Uiso 1 1 calc R U . . .

loop_
 _atom_site_aniso_label
 _atom_site_aniso_U_11
 _atom_site_aniso_U_22
 _atom_site_aniso_U_33
 _atom_site_aniso_U_23
 _atom_site_aniso_U_13
 _atom_site_aniso_U_12
S2A 0.0130(3) 0.0315(3) 0.0239(3) -0.0005(2) 0.0020(2) 0.0023(2)
O5A 0.0187(7) 0.0227(8) 0.0411(10) -0.0001(7) 0.0046(7) 0.0024(6)
N1A 0.0144(9) 0.0379(12) 0.0243(10) 0.0008(8) 0.0027(7) 0.0035(8)
N3A 0.0115(8) 0.0302(10) 0.0252(9) 0.0020(8) 0.0028(7) 0.0005(7)
N4A 0.0113(8) 0.0221(10) 0.0271(10) 0.0003(8) 0.0010(7) 0.0017(7)
N54A 0.0159(8) 0.0338(11) 0.0239(9) 0.0019(8) 0.0040(7) -0.0010(8)
F12A 0.0456(9) 0.0483(10) 0.0490(10) 0.0006(8) 0.0108(8) -0.0004(8)
C2A 0.0178(10) 0.0204(11) 0.0242(10) -0.0019(8) 0.0036(8) 0.0005(8)
C5A 0.0164(10) 0.0247(11) 0.0221(10) 0.0034(8) 0.0043(8) 0.0006(8)
C11A 0.0163(10) 0.0557(17) 0.0227(11) 0.0006(11) 0.0064(9) 0.0090(10)
C12A 0.0232(11) 0.0607(19) 0.0338(13) 0.0138(13) 0.0116(10) 0.0135(12)
C13A 0.0351(14) 0.078(2) 0.0434(16) 0.0210(16) 0.0173(13) 0.0164(15)
C14A 0.0336(15) 0.097(3) 0.0399(17) 0.0134(18) 0.0144(13) 0.0171(18)
C15A 0.0320(15) 0.115(4) 0.0302(15) -0.0205(18) 0.0032(12) 0.0128(18)
C16A 0.0305(14) 0.081(2) 0.0321(14) -0.0151(15) 0.0041(11) 0.0057(14)
C51A 0.0162(10) 0.0228(11) 0.0214(10) 0.0017(8) 0.0034(8) -0.0003(8)
C52A 0.0200(10) 0.0261(12) 0.0276(11) -0.0028(9) 0.0022(9) -0.0009(9)
C53A 0.0191(10) 0.0296(12) 0.0264(11) -0.0019(9) 0.0020(8) -0.0042(9)
C55A 0.0184(10) 0.0280(12) 0.0296(11) 0.0045(9) 0.0050(9) 0.0025(9)
C56A 0.0172(10) 0.0230(11) 0.0278(11) 0.0038(9) 0.0047(8) -0.0001(8)
S2B 0.0132(3) 0.0306(3) 0.0252(3) 0.0032(2) 0.0028(2) 0.00240(19)
O5B 0.0187(7) 0.0230(8) 0.0389(9) -0.0002(7) 0.0042(7) 0.0031(6)
N1B 0.0137(8) 0.0327(11) 0.0243(10) 0.0056(8) 0.0034(7) 0.0031(8)
N3B 0.0121(8) 0.0308(11) 0.0231(9) 0.0030(8) 0.0024(7) -0.0001(7)
N4B 0.0116(8) 0.0235(10) 0.0263(9) 0.0009(8) 0.0015(7) 0.0013(7)
N54B 0.0159(8) 0.0320(11) 0.0242(9) 0.0006(8) 0.0032(7) 0.0004(7)
F12B 0.0402(9) 0.0381(9) 0.0481(9) 0.0016(7) 0.0073(7) -0.0052(7)
C2B 0.0176(10) 0.0184(10) 0.0250(11) 0.0008(8) 0.0038(8) 0.0001(8)
C5B 0.0175(10) 0.0244(11) 0.0209(10) 0.0022(8) 0.0052(8) 0.0006(8)
C11B 0.0162(10) 0.0363(13) 0.0252(11) 0.0037(10) 0.0065(8) 0.0073(9)
C12B 0.0215(11) 0.0342(13) 0.0351(13) 0.0066(10) 0.0095(9) 0.0060(10)
C13B 0.0317(13) 0.0451(16) 0.0413(15) 0.0136(12) 0.0168(11) 0.0084(12)
C14B 0.0336(13) 0.0560(18) 0.0320(13) 0.0118(12) 0.0143(11) 0.0139(13)
C15B 0.0311(13) 0.0563(18) 0.0307(13) -0.0062(12) 0.0053(11) 0.0089(12)
C16B 0.0262(12) 0.0380(14) 0.0304(12) -0.0010(11) 0.0043(10) 0.0034(10)
C51B 0.0165(10) 0.0236(11) 0.0196(10) 0.0003(8) 0.0032(8) 0.0005(8)
C52B 0.0197(10) 0.0248(12) 0.0306(12) -0.0041(9) 0.0011(9) -0.0003(9)
C53B 0.0189(10) 0.0292(12) 0.0290(12) -0.0041(9) 0.0017(9) -0.0029(9)
C55B 0.0197(10) 0.0268(12) 0.0269(11) 0.0031(9) 0.0046(9) 0.0035(9)
C56B 0.0186(10) 0.0229(11) 0.0247(11) 0.0025(9) 0.0041(8) -0.0011(8)
O2 0.0563(14) 0.114(2) 0.0395(12) 0.0129(14) -0.0024(11) -0.0272(15)
C1 0.089(3) 0.069(3) 0.054(2) -0.004(2) -0.006(2) -0.006(2)
C2 0.064(2) 0.056(2) 0.0431(18) 0.0044(15) -0.0014(15) 0.0151(17)
C3 0.054(2) 0.072(3) 0.069(2) 0.006(2) 0.0076(18) -0.0081(19)

_geom_special_details
;
 All esds (except the esd in the dihedral angle between two l.s. planes)
 are estimated using the full covariance matrix. The cell esds are taken
 into account individually in the estimation of esds in distances, angles
 and torsion angles; correlations between esds in cell parameters are only
 used when they are defined by crystal symmetry. An approximate (isotropic)
 treatment of cell esds is used for estimating esds involving l.s. planes.
;

loop_
 _geom_bond_atom_site_label_1
 _geom_bond_atom_site_label_2
 _geom_bond_distance
 _geom_bond_site_symmetry_2
 _geom_bond_publ_flag
S2A C2A 1.695(2) . ?
O5A C5A 1.226(3) . ?
N1A C2A 1.333(3) . ?
N1A C11A 1.420(3) . ?
N1A H1A 0.89(3) . ?
N3A C2A 1.349(3) . ?
N3A N4A 1.392(2) . ?
N3A H3A 0.83(3) . ?
N4A C5A 1.353(3) . ?
N4A H4A 0.78(3) . ?
N54A C53A 1.338(3) . ?
N54A C55A 1.338(3) . ?
F12A C12A 1.358(4) . ?
C5A C51A 1.506(3) . ?
C11A C16A 1.379(4) . ?
C11A C12A 1.399(4) . ?
C12A C13A 1.380(4) . ?
C13A C14A 1.354(5) . ?
C13A H13A 0.9300 . ?
C14A C15A 1.359(6) . ?
C14A H14A 0.9300 . ?
C15A C16A 1.441(5) . ?
C15A H15A 0.9300 . ?
C16A H16A 0.9300 . ?
C51A C52A 1.391(3) . ?
C51A C56A 1.392(3) . ?
C52A C53A 1.382(3) . ?
C52A H52A 0.9300 . ?
C53A H53A 0.9300 . ?
C55A C56A 1.391(3) . ?
C55A H55A 0.9300 . ?
C56A H56A 0.9300 . ?
S2B C2B 1.690(2) . ?
O5B C5B 1.226(3) . ?
N1B C2B 1.333(3) . ?
N1B C11B 1.420(3) . ?
N1B H1B 0.81(3) . ?
N3B C2B 1.353(3) . ?
N3B N4B 1.390(2) . ?
N3B H3B 0.82(3) . ?
N4B C5B 1.352(3) . ?
N4B H4B 0.84(3) . ?
N54B C55B 1.336(3) . ?
N54B C53B 1.339(3) . ?
F12B C12B 1.363(3) . ?
C5B C51B 1.502(3) . ?
C11B C16B 1.385(4) . ?
C11B C12B 1.389(4) . ?
C12B C13B 1.377(4) . ?
C13B C14B 1.375(4) . ?
C13B H13B 0.9300 . ?
C14B C15B 1.378(5) . ?
C14B H14B 0.9300 . ?
C15B C16B 1.399(4) . ?

C15B H15B 0.9300 . ?
C16B H16B 0.9300 . ?
C51B C52B 1.393(3) . ?
C51B C56B 1.393(3) . ?
C52B C53B 1.385(3) . ?
C52B H52B 0.9300 . ?
C53B H53B 0.9300 . ?
C55B C56B 1.389(3) . ?
C55B H55B 0.9300 . ?
C56B H56B 0.9300 . ?
O2 C2 1.456(5) . ?
O2 H1 0.8200 . ?
C1 C2 1.493(5) . ?
C1 H11 0.9600 . ?
C1 H12 0.9600 . ?
C1 H13 0.9600 . ?
C2 C3 1.494(5) . ?
C2 H21 0.9800 . ?
C3 H31 0.9600 . ?
C3 H32 0.9600 . ?
C3 H33 0.9600 . ?

loop_
_geom_angle_atom_site_label_1
_geom_angle_atom_site_label_2
_geom_angle_atom_site_label_3
_geom_angle
_geom_angle_site_symmetry_1
_geom_angle_site_symmetry_3
_geom_angle_publ_flag
C2A N1A C11A 123.54(19) . . ?
C2A N1A H1A 117(2) . . ?
C11A N1A H1A 117(2) . . ?
C2A N3A N4A 121.12(19) . . ?
C2A N3A H3A 121(2) . . ?
N4A N3A H3A 115(2) . . ?
C5A N4A N3A 119.51(19) . . ?
C5A N4A H4A 126(2) . . ?
N3A N4A H4A 114(2) . . ?
C53A N54A C55A 117.19(19) . . ?
N1A C2A N3A 117.67(19) . . ?
N1A C2A S2A 124.03(17) . . ?
N3A C2A S2A 118.24(16) . . ?
O5A C5A N4A 122.1(2) . . ?
O5A C5A C51A 121.8(2) . . ?
N4A C5A C51A 116.11(19) . . ?
C16A C11A C12A 118.3(3) . . ?
C16A C11A N1A 123.4(3) . . ?
C12A C11A N1A 118.2(2) . . ?
F12A C12A C13A 118.5(3) . . ?
F12A C12A C11A 118.2(2) . . ?
C13A C12A C11A 123.3(3) . . ?
C14A C13A C12A 117.7(4) . . ?
C14A C13A H13A 121.1 . . ?
C12A C13A H13A 121.1 . . ?
C13A C14A C15A 122.1(3) . . ?
C13A C14A H14A 118.9 . . ?
C15A C14A H14A 118.9 . . ?
C14A C15A C16A 120.5(3) . . ?
C14A C15A H15A 119.8 . . ?
C16A C15A H15A 119.8 . . ?
C11A C16A C15A 118.0(4) . . ?
C11A C16A H16A 121.0 . . ?
C15A C16A H16A 121.0 . . ?
C52A C51A C56A 118.1(2) . . ?
C52A C51A C5A 117.3(2) . . ?
C56A C51A C5A 124.5(2) . . ?
C53A C52A C51A 119.2(2) . . ?
C53A C52A H52A 120.4 . . ?
C51A C52A H52A 120.4 . . ?

N54A C53A C52A 123.3(2) . . ?
N54A C53A H53A 118.3 . . ?
C52A C53A H53A 118.3 . . ?
N54A C55A C56A 123.7(2) . . ?
N54A C55A H55A 118.2 . . ?
C56A C55A H55A 118.2 . . ?
C55A C56A C51A 118.4(2) . . ?
C55A C56A H56A 120.8 . . ?
C51A C56A H56A 120.8 . . ?
C2B N1B C11B 124.62(19) . . ?
C2B N1B H1B 121(2) . . ?
C11B N1B H1B 114(2) . . ?
C2B N3B N4B 121.91(19) . . ?
C2B N3B H3B 122(2) . . ?
N4B N3B H3B 114(2) . . ?
C5B N4B N3B 119.71(19) . . ?
C5B N4B H4B 125(2) . . ?
N3B N4B H4B 115(2) . . ?
C55B N54B C53B 117.27(19) . . ?
N1B C2B N3B 117.10(19) . . ?
N1B C2B S2B 124.21(17) . . ?
N3B C2B S2B 118.62(16) . . ?
O5B C5B N4B 121.94(19) . . ?
O5B C5B C51B 121.8(2) . . ?
N4B C5B C51B 116.23(19) . . ?
C16B C11B C12B 118.2(2) . . ?
C16B C11B N1B 122.8(2) . . ?
C12B C11B N1B 118.9(2) . . ?
F12B C12B C13B 119.4(2) . . ?
F12B C12B C11B 118.0(2) . . ?
C13B C12B C11B 122.7(3) . . ?
C14B C13B C12B 118.3(3) . . ?
C14B C13B H13B 120.9 . . ?
C12B C13B H13B 120.9 . . ?
C13B C14B C15B 121.0(3) . . ?
C13B C14B H14B 119.5 . . ?
C15B C14B H14B 119.5 . . ?
C14B C15B C16B 120.1(3) . . ?
C14B C15B H15B 120.0 . . ?
C16B C15B H15B 120.0 . . ?
C11B C16B C15B 119.8(3) . . ?
C11B C16B H16B 120.1 . . ?
C15B C16B H16B 120.1 . . ?
C52B C51B C56B 117.99(19) . . ?
C52B C51B C5B 117.0(2) . . ?
C56B C51B C5B 124.9(2) . . ?
C53B C52B C51B 119.3(2) . . ?
C53B C52B H52B 120.4 . . ?
C51B C52B H52B 120.4 . . ?
N54B C53B C52B 123.1(2) . . ?
N54B C53B H53B 118.5 . . ?
C52B C53B H53B 118.5 . . ?
N54B C55B C56B 123.9(2) . . ?
N54B C55B H55B 118.1 . . ?
C56B C55B H55B 118.1 . . ?
C55B C56B C51B 118.4(2) . . ?
C55B C56B H56B 120.8 . . ?
C51B C56B H56B 120.8 . . ?
C2 O2 H1 109.5 . . ?
C2 C1 H11 109.5 . . ?
C2 C1 H12 109.5 . . ?
H11 C1 H12 109.5 . . ?
C2 C1 H13 109.5 . . ?
H11 C1 H13 109.5 . . ?
H12 C1 H13 109.5 . . ?
O2 C2 C1 105.9(3) . . ?
O2 C2 C3 107.8(3) . . ?
C1 C2 C3 113.4(4) . . ?
O2 C2 H21 109.9 . . ?
C1 C2 H21 109.9 . . ?

C3 C2 H21 109.9 . . ?
C2 C3 H31 109.5 . . ?
C2 C3 H32 109.5 . . ?
H31 C3 H32 109.5 . . ?
C2 C3 H33 109.5 . . ?
H31 C3 H33 109.5 . . ?
H32 C3 H33 109.5 . . ?

loop_
 _geom_torsion_atom_site_label_1
 _geom_torsion_atom_site_label_2
 _geom_torsion_atom_site_label_3
 _geom_torsion_atom_site_label_4
 _geom_torsion
 _geom_torsion_site_symmetry_1
 _geom_torsion_site_symmetry_2
 _geom_torsion_site_symmetry_3
 _geom_torsion_site_symmetry_4
 _geom_torsion_publ_flag
C2A N3A N4A C5A -87.1(3) . . . . ?
C11A N1A C2A N3A 172.9(2) . . . . ?
C11A N1A C2A S2A -9.9(3) . . . . ?
N4A N3A C2A N1A -10.7(3) . . . . ?
N4A N3A C2A S2A 171.92(16) . . . . ?
N3A N4A C5A O5A 3.1(3) . . . . ?
N3A N4A C5A C51A -179.79(18) . . . . ?
C2A N1A C11A C16A 86.8(3) . . . . ?
C2A N1A C11A C12A -96.6(3) . . . . ?
C16A C11A C12A F12A 179.1(2) . . . . ?
N1A C11A C12A F12A 2.3(3) . . . . ?
C16A C11A C12A C13A -1.1(4) . . . . ?
N1A C11A C12A C13A -177.9(2) . . . . ?
F12A C12A C13A C14A -178.8(2) . . . . ?
C11A C12A C13A C14A 1.4(4) . . . . ?
C12A C13A C14A C15A -1.0(4) . . . . ?
C13A C14A C15A C16A 0.4(5) . . . . ?
C12A C11A C16A C15A 0.4(4) . . . . ?
N1A C11A C16A C15A 177.1(2) . . . . ?
C14A C15A C16A C11A -0.1(4) . . . . ?
O5A C5A C51A C52A 8.7(3) . . . . ?
N4A C5A C51A C52A -168.5(2) . . . . ?
O5A C5A C51A C56A -175.5(2) . . . . ?
N4A C5A C51A C56A 7.3(3) . . . . ?
C56A C51A C52A C53A -3.6(3) . . . . ?
C5A C51A C52A C53A 172.5(2) . . . . ?
C55A N54A C53A C52A 2.2(3) . . . . ?
C51A C52A C53A N54A 0.9(4) . . . . ?
C53A N54A C55A C56A -2.7(3) . . . . ?
N54A C55A C56A C51A 0.0(3) . . . . ?
C52A C51A C56A C55A 3.1(3) . . . . ?
C5A C51A C56A C55A -172.6(2) . . . . ?
C2B N3B N4B C5B 86.2(3) . . . . ?
C11B N1B C2B N3B -177.1(2) . . . . ?
C11B N1B C2B S2B 6.0(3) . . . . ?
N4B N3B C2B N1B 8.6(3) . . . . ?
N4B N3B C2B S2B -174.28(17) . . . . ?
N3B N4B C5B O5B -2.9(3) . . . . ?
N3B N4B C5B C51B 179.15(18) . . . . ?
C2B N1B C11B C16B -77.5(3) . . . . ?
C2B N1B C11B C12B 107.2(3) . . . . ?
C16B C11B C12B F12B -179.9(2) . . . . ?
N1B C11B C12B F12B -4.4(3) . . . . ?
C16B C11B C12B C13B -0.1(3) . . . . ?
N1B C11B C12B C13B 175.5(2) . . . . ?
F12B C12B C13B C14B 179.5(2) . . . . ?
C11B C12B C13B C14B -0.4(4) . . . . ?
C12B C13B C14B C15B 0.3(4) . . . . ?
C13B C14B C15B C16B 0.1(4) . . . . ?
C12B C11B C16B C15B 0.5(3) . . . . ?
N1B C11B C16B C15B -174.8(2) . . . . ?

```
C14B C15B C16B C11B -0.6(4) . . . . ?
O5B C5B C51B C52B -7.0(3) . . . . ?
N4B C5B C51B C52B 171.0(2) . . . . ?
O5B C5B C51B C56B 177.9(2) . . . . ?
N4B C5B C51B C56B -4.2(3) . . . . ?
C56B C51B C52B C53B 3.3(3) . . . . ?
C5B C51B C52B C53B -172.2(2) . . . . ?
C55B N54B C53B C52B -2.4(3) . . . . ?
C51B C52B C53B N54B -0.5(4) . . . . ?
C53B N54B C55B C56B 2.7(3) . . . . ?
N54B C55B C56B C51B 0.1(3) . . . . ?
C52B C51B C56B C55B -3.1(3) . . . . ?
C5B C51B C56B C55B 172.0(2) . . . . ?

_refine_diff_density_max    1.094
_refine_diff_density_min   -0.587
_refine_diff_density_rms    0.075

_shelx_res_file
;

    shelx.res created by SHELXL-2014/7


TITL mm34 in P21/n
CELL  1.54184  13.4658   9.8028  23.9666   90.000  104.402   90.000
ZERR     8.00   0.0002   0.0001   0.0003    0.000    0.001    0.000
LATT   1
SYMM  1/2 - X, 1/2 + Y, 1/2 - Z
SFAC  C    H    F    N    O    S
UNIT  116  120  8    32   12   8
MERG   2
FMAP   2
ACTA
BOND   $H
CONF
PLAN  -6
L.S.  40
WGHT    0.088000    3.792200
FVAR       0.25665
MOLE    1
S2A   6    0.027920    0.226972    0.343911    11.00000    0.01298    0.03152 =
        0.02391   -0.00052    0.00195    0.00229
O5A   5   -0.258721    0.094980    0.218529    11.00000    0.01871    0.02269 =
        0.04106   -0.00014    0.00463    0.00243
N1A   4   -0.163761    0.254983    0.355669    11.00000    0.01442    0.03785 =
        0.02432    0.00083    0.00272    0.00354
H1A   2   -0.230113    0.267505    0.339277    11.00000   -1.50000
N3A   4   -0.136943    0.303531    0.266894    11.00000    0.01150    0.03022 =
        0.02517    0.00204    0.00282    0.00048
H3A   2   -0.102480    0.297634    0.242613    11.00000   -1.50000
N4A   4   -0.242002    0.317249    0.243764    11.00000    0.01134    0.02209 =
        0.02711    0.00035    0.00102    0.00174
H4A   2   -0.262908    0.390675    0.245522    11.00000   -1.50000
N54A  4   -0.624067    0.253940    0.161290    11.00000    0.01591    0.03381 =
        0.02388    0.00188    0.00405   -0.00096
F12A  3   -0.210637   -0.009118    0.371395    11.00000    0.04562    0.04832 =
        0.04903    0.00059    0.01077   -0.00041
C2A   1   -0.099188    0.261520    0.321691    11.00000    0.01782    0.02035 =
        0.02419   -0.00193    0.00361    0.00050
C5A   1   -0.297350    0.208086    0.219130    11.00000    0.01635    0.02470 =
        0.02214    0.00338    0.00435    0.00063
C11A  1   -0.137287    0.198723    0.412033    11.00000    0.01635    0.05567 =
        0.02265    0.00059    0.00639    0.00904
C12A  1   -0.163990    0.063061    0.419101    11.00000    0.02317    0.06074 =
        0.03378    0.01384    0.01156    0.01346
C13A  1   -0.145073   -0.000989    0.471974    11.00000    0.03506    0.07784 =
        0.04336    0.02100    0.01733    0.01639
AFIX  43
H13A  2   -0.162507   -0.091979    0.475073    11.00000   -1.50000
```

```
AFIX   0
C14A  1   -0.100249    0.073191    0.519197    11.00000    0.03364    0.09730 =
        0.03994    0.01342    0.01435    0.01707
AFIX  43
H14A  2   -0.087874    0.032145    0.555256    11.00000   -1.50000
AFIX   0
C15A  1   -0.072773    0.205659    0.515590    11.00000    0.03197    0.11503 =
        0.03018   -0.02050    0.00323    0.01279
AFIX  43
H15A  2   -0.041788    0.253300    0.548933    11.00000   -1.50000
AFIX   0
C16A  1   -0.091297    0.272882    0.460471    11.00000    0.03047    0.08062 =
        0.03207   -0.01512    0.00409    0.00569
AFIX  43
H16A  2   -0.072790    0.363544    0.457656    11.00000   -1.50000
AFIX   0
C51A  1   -0.410365    0.231801    0.194985    11.00000    0.01620    0.02275 =
        0.02141    0.00167    0.00337   -0.00033
C52A  1   -0.472029    0.117360    0.179112    11.00000    0.01999    0.02611 =
        0.02755   -0.00283    0.00216   -0.00087
AFIX  43
H52A  2   -0.442760    0.031388    0.179260    11.00000   -1.50000
AFIX   0
C53A  1   -0.577283    0.132996    0.163125    11.00000    0.01913    0.02961 =
        0.02638   -0.00186    0.00198   -0.00422
AFIX  43
H53A  2   -0.617666    0.055533    0.153132    11.00000   -1.50000
AFIX   0
C55A  1   -0.564224    0.364326    0.173940    11.00000    0.01838    0.02798 =
        0.02958    0.00450    0.00498    0.00253
AFIX  43
H55A  2   -0.595594    0.449423    0.171243    11.00000   -1.50000
AFIX   0
C56A  1   -0.457741    0.358907    0.190893    11.00000    0.01718    0.02299 =
        0.02775    0.00381    0.00469   -0.00013
AFIX  43
H56A  2   -0.419104    0.438304    0.199297    11.00000   -1.50000
AFIX   0
MOLE    2
S2B   6   -0.035376    0.312217    0.155162    11.00000    0.01317    0.03063 =
        0.02516    0.00324    0.00284    0.00240
O5B   5    0.254562    0.452786    0.278553    11.00000    0.01869    0.02299 =
        0.03887   -0.00018    0.00420    0.00311
N1B   4    0.157029    0.290577    0.144225    11.00000    0.01374    0.03272 =
        0.02432    0.00556    0.00336    0.00310
H1B   2    0.217589    0.277683    0.157259    11.00000   -1.50000
N3B   4    0.130891    0.243491    0.233143    11.00000    0.01206    0.03083 =
        0.02308    0.00297    0.00240   -0.00011
H3B   2    0.097132    0.248009    0.257198    11.00000   -1.50000
N4B   4    0.235741    0.229159    0.256403    11.00000    0.01163    0.02354 =
        0.02626    0.00090    0.00145    0.00127
H4B   2    0.258922    0.149775    0.255410    11.00000   -1.50000
N54B  4    0.618302    0.290908    0.339001    11.00000    0.01590    0.03203 =
        0.02421    0.00063    0.00321    0.00042
F12B  3    0.215861    0.542319    0.117679    11.00000    0.04017    0.03808 =
        0.04806    0.00158    0.00733   -0.00522
C2B   1    0.091995    0.283086    0.177925    11.00000    0.01764    0.01845 =
        0.02498    0.00082    0.00380    0.00005
C5B   1    0.292243    0.338763    0.279446    11.00000    0.01747    0.02443 =
        0.02089    0.00219    0.00522    0.00063
C11B  1    0.130436    0.335802    0.086109    11.00000    0.01616    0.03632 =
        0.02522    0.00367    0.00645    0.00726
C12B  1    0.164332    0.462978    0.073157    11.00000    0.02150    0.03423 =
        0.03514    0.00656    0.00949    0.00601
C13B  1    0.148010    0.510821    0.017602    11.00000    0.03172    0.04511 =
        0.04131    0.01361    0.01676    0.00841
AFIX  43
H13B  2    0.171212    0.596647    0.010168    11.00000   -1.50000
AFIX   0
C14B  1    0.096519    0.428419   -0.026597    11.00000    0.03364    0.05596 =
```

```
        0.03197    0.01184    0.01433    0.01388
AFIX  43
H14B  2    0.085085    0.458811   -0.064423    11.00000   -1.50000
AFIX   0
C15B  1    0.061593    0.301488   -0.015708    11.00000    0.03113    0.05629 =
        0.03073   -0.00621    0.00529    0.00886
AFIX  43
H15B  2    0.027020    0.246888   -0.046093    11.00000   -1.50000
AFIX   0
C16B  1    0.078079    0.254627    0.041018    11.00000    0.02624    0.03804 =
        0.03043   -0.00098    0.00430    0.00338
AFIX  43
H16B  2    0.053977    0.169310    0.048417    11.00000   -1.50000
AFIX   0
C51B  1    0.404633    0.313834    0.304388    11.00000    0.01649    0.02355 =
        0.01960    0.00033    0.00316    0.00050
C52B  1    0.466498    0.428091    0.320675    11.00000    0.01974    0.02480 =
        0.03061   -0.00406    0.00107   -0.00034
AFIX  43
H52B  2    0.437320    0.514160    0.320384    11.00000   -1.50000
AFIX   0
C53B  1    0.571931    0.412167    0.337310    11.00000    0.01895    0.02923 =
        0.02902   -0.00411    0.00174   -0.00293
AFIX  43
H53B  2    0.612434    0.489349    0.347801    11.00000   -1.50000
AFIX   0
C55B  1    0.558269    0.181080    0.325809    11.00000    0.01969    0.02678 =
        0.02691    0.00314    0.00462    0.00348
AFIX  43
H55B  2    0.589526    0.095920    0.328404    11.00000   -1.50000
AFIX   0
C56B  1    0.451969    0.186555    0.308453    11.00000    0.01857    0.02291 =
        0.02470    0.00249    0.00410   -0.00111
AFIX  43
H56B  2    0.413330    0.107229    0.299759    11.00000   -1.50000
AFIX   0
MOLE    3
O2    5    0.169153    0.295991    0.477663    11.00000    0.05631    0.11370 =
        0.03951    0.01287   -0.00241   -0.02719
AFIX 143
H1    2    0.129174    0.277447    0.446783    11.00000   -1.50000
AFIX   0
C1    1    0.286194    0.454718    0.531026    11.00000    0.08900    0.06850 =
        0.05420   -0.00393   -0.00615   -0.00598
AFIX  33
H11   2    0.322999    0.538148    0.529939    11.00000   -1.50000
H12   2    0.242895    0.464560    0.557115    11.00000   -1.50000
H13   2    0.334011    0.381813    0.543880    11.00000   -1.50000
AFIX   0
C2    1    0.221926    0.423042    0.472147    11.00000    0.06413    0.05585 =
        0.04309    0.00441   -0.00136    0.01509
AFIX  13
H21   2    0.171763    0.496029    0.459172    11.00000   -1.50000
AFIX   0
C3    1    0.283037    0.401111    0.428756    11.00000    0.05419    0.07176 =
        0.06853    0.00643    0.00761   -0.00810
AFIX  33
H31   2    0.318683    0.483632    0.424308    11.00000   -1.50000
H32   2    0.331776    0.329228    0.441610    11.00000   -1.50000
H33   2    0.237933    0.376375    0.392459    11.00000   -1.50000
AFIX   0
HKLF    4

REM  mm34 in P21/n
REM R1 =  0.0567 for    5725 Fo > 4sig(Fo)  and  0.0605 for all    6328 data
REM    415 parameters refined using      0 restraints

END

WGHT      0.0878      3.7974
```

```
REM Highest difference peak  1.094,  deepest hole -0.587,  1-sigma level  0.075
Q1    1   0.1992  0.3737  0.5656  11.00000  0.05    1.09
Q2    1   0.1781  0.3975  0.5044  11.00000  0.05    0.78
Q3    1   0.0503  0.1309  0.0574  11.00000  0.05    0.74
Q4    1  -0.0578  0.4428  0.4350  11.00000  0.05    0.64
Q5    1  -0.0484  0.3066  0.5112  11.00000  0.05    0.56
Q6    1   0.2691  0.5047  0.4920  11.00000  0.05    0.52
;
```

CIF for 14

```
data_shelx

_audit_creation_method            'SHELXL-2014/7'
_shelx_SHELXL_version_number      '2014/7'
_chemical_name_systematic         ?
_chemical_name_common             ?
_chemical_melting_point           ?
_chemical_formula_moiety          ?
_chemical_formula_sum
 'C14 H14 Cl N5 O2 S'
_chemical_formula_weight          351.81

loop_
 _atom_type_symbol
 _atom_type_description
 _atom_type_scat_dispersion_real
 _atom_type_scat_dispersion_imag
 _atom_type_scat_source
 'C'  'C'   0.0181   0.0091
 'International Tables Vol C Tables 4.2.6.8 and 6.1.1.4'
 'H'  'H'   0.0000   0.0000
 'International Tables Vol C Tables 4.2.6.8 and 6.1.1.4'
 'Cl'  'Cl'   0.3639   0.7018
 'International Tables Vol C Tables 4.2.6.8 and 6.1.1.4'
 'N'  'N'   0.0311   0.0180
 'International Tables Vol C Tables 4.2.6.8 and 6.1.1.4'
 'O'  'O'   0.0492   0.0322
 'International Tables Vol C Tables 4.2.6.8 and 6.1.1.4'
 'S'  'S'   0.3331   0.5567
 'International Tables Vol C Tables 4.2.6.8 and 6.1.1.4'

_space_group_crystal_system       monoclinic
_space_group_IT_number            15
_space_group_name_H-M_alt         'C 2/c'
_space_group_name_Hall            '-C 2yc'

_shelx_space_group_comment
;
The symmetry employed for this shelxl refinement is uniquely defined
by the following loop, which should always be used as a source of
symmetry information in preference to the above space-group names.
They are only intended as comments.
;

loop_
 _space_group_symop_operation_xyz
 'x, y, z'
 '-x, y, -z+1/2'
 'x+1/2, y+1/2, z'
 '-x+1/2, y+1/2, -z+1/2'
 '-x, -y, -z'
 'x, -y, z-1/2'
 '-x+1/2, -y+1/2, -z'
 'x+1/2, -y+1/2, z-1/2'

_cell_length_a                    24.0692(5)
_cell_length_b                    9.7448(2)
_cell_length_c                    13.4379(3)
```

```
_cell_angle_alpha                90
_cell_angle_beta                 103.383(2)
_cell_angle_gamma                90
_cell_volume                     3066.26(12)
_cell_formula_units_Z            8
_cell_measurement_temperature     293(2)
_cell_measurement_reflns_used     237
_cell_measurement_theta_min       4.90
_cell_measurement_theta_max       77.50

_exptl_crystal_description        prism
_exptl_crystal_colour             colourless
_exptl_crystal_density_meas       ?
_exptl_crystal_density_method     ?
_exptl_crystal_density_diffrn     1.524
_exptl_crystal_F_000              1456
_exptl_transmission_factor_min    ?
_exptl_transmission_factor_max    ?
_exptl_crystal_size_max           0.50
_exptl_crystal_size_mid           0.16
_exptl_crystal_size_min           0.03
_exptl_absorpt_coefficient_mu     3.640
_shelx_estimated_absorpt_T_min    ?
_shelx_estimated_absorpt_T_max    ?
_exptl_absorpt_correction_type    multi-scan
_exptl_absorpt_correction_T_min   0.3444
_exptl_absorpt_correction_T_max   1.0000
_exptl_absorpt_process_details
;
CrysAlisPro 1.171.39.16b (Rigaku Oxford Diffraction, 2015)
Numerical absorption correction based on gaussian integration over
                                        a multifaceted crystal model
Empirical absorption correction using spherical harmonics,
 implemented in SCALE3 ABSPACK scaling algorithm.
;
_exptl_absorpt_special_details    ?
_diffrn_ambient_temperature       293(2)
_diffrn_radiation_wavelength      1.54184
_diffrn_radiation_type            CuK\a
_diffrn_source                    ?
_diffrn_measurement_device_type   'Rigaku Oxford Diffraction XtaLAB Synergy, Pilatus 300K diffractometer'
_diffrn_measurement_method        '\w scans'
_diffrn_detector_area_resol_mean  ?
_diffrn_reflns_number             8718
_diffrn_reflns_av_unetI/netI      0.0328
_diffrn_reflns_av_R_equivalents   0.0376
_diffrn_reflns_limit_h_min        -28
_diffrn_reflns_limit_h_max        30
_diffrn_reflns_limit_k_min        -12
_diffrn_reflns_limit_k_max        11
_diffrn_reflns_limit_l_min        -16
_diffrn_reflns_limit_l_max        6
_diffrn_reflns_theta_min          3.775
_diffrn_reflns_theta_max          78.547
_diffrn_reflns_theta_full         67.684
_diffrn_measured_fraction_theta_max   0.952
_diffrn_measured_fraction_theta_full  0.996
_diffrn_reflns_Laue_measured_fraction_max    0.952
_diffrn_reflns_Laue_measured_fraction_full   0.996
_diffrn_reflns_point_group_measured_fraction_max   0.952
_diffrn_reflns_point_group_measured_fraction_full  0.996
_reflns_number_total              3143
_reflns_number_gt                 2901
_reflns_threshold_expression      'I > 2\s(I)'
_reflns_Friedel_coverage          0.000
_reflns_Friedel_fraction_max      .
_reflns_Friedel_fraction_full     .

_reflns_special_details
;
```

Reflections were merged by SHELXL according to the crystal
class for the calculation of statistics and refinement.

_reflns_Friedel_fraction is defined as the number of unique
Friedel pairs measured divided by the number that would be
possible theoretically, ignoring centric projections and
systematic absences.
;

```
_computing_data_collection        'CrysAlisPro 1.171.39.16b (Rigaku OD, 2015)'
_computing_cell_refinement        'CrysAlisPro 1.171.39.16b (Rigaku OD, 2015)'
_computing_data_reduction         'CrysAlisPro 1.171.39.16b (Rigaku OD, 2015)'
_computing_structure_solution     'SHELXS-2013/1 (Sheldrick, 2014)'
_computing_structure_refinement 'SHELXL-2014/7 (Sheldrick, 2014)'
_computing_molecular_graphics     'ORTEP3 for Windows'
_computing_publication_material   'SHELXL-2014/7 and WINGX'
_refine_special_details           ?
_refine_ls_structure_factor_coef  Fsqd
_refine_ls_matrix_type            full
_refine_ls_weighting_scheme       calc
_refine_ls_weighting_details
'w=1/[\s^2^(Fo^2^)+(0.1112P)^2^+7.2925P] where P=(Fo^2^+2Fc^2^)/3'
_atom_sites_solution_primary      difmap
_atom_sites_solution_secondary    difmap
_atom_sites_solution_hydrogens    mixed
_refine_ls_hydrogen_treatment     mixed
_refine_ls_extinction_method      none
_refine_ls_extinction_coef        .
_refine_ls_number_reflns          3143
_refine_ls_number_parameters      235
_refine_ls_number_restraints      15
_refine_ls_R_factor_all           0.0688
_refine_ls_R_factor_gt            0.0661
_refine_ls_wR_factor_ref          0.1919
_refine_ls_wR_factor_gt           0.1890
_refine_ls_goodness_of_fit_ref    1.125
_refine_ls_restrained_S_all       1.132
_refine_ls_shift/su_max           0.000
_refine_ls_shift/su_mean          0.000

loop_
 _atom_site_label
 _atom_site_type_symbol
 _atom_site_fract_x
 _atom_site_fract_y
 _atom_site_fract_z
 _atom_site_U_iso_or_equiv
 _atom_site_adp_type
 _atom_site_occupancy
 _atom_site_site_symmetry_order
 _atom_site_calc_flag
 _atom_site_refinement_flags_posn
 _atom_site_refinement_flags_adp
 _atom_site_refinement_flags_occupancy
 _atom_site_disorder_assembly
 _atom_site_disorder_group
Cl12 Cl 0.38777(4) -0.05295(8) 0.27896(6) 0.0446(3) Uani 1 1 d . . . . .
S2 S 0.34476(3) 0.20423(7) 0.53257(5) 0.0309(2) Uani 1 1 d . . . . .
O5 O 0.21855(11) 0.0596(2) 0.24156(15) 0.0390(5) Uani 1 1 d . . . . .
N1 N 0.35812(11) 0.2259(3) 0.34078(17) 0.0298(5) Uani 1 1 d . . . . .
H1 H 0.3445(17) 0.239(4) 0.274(3) 0.045 Uiso 1 1 d . U . . .
N3 N 0.26808(11) 0.2635(3) 0.36561(17) 0.0320(5) Uani 1 1 d . . . . .
H3 H 0.2359(17) 0.266(4) 0.405(3) 0.048 Uiso 1 1 d . U . . .
N4 N 0.24621(11) 0.2806(3) 0.26136(17) 0.0287(5) Uani 1 1 d . . . . .
H4 H 0.2513(17) 0.361(5) 0.238(3) 0.043 Uiso 1 1 d . U . . .
N54 N 0.16221(10) 0.2328(3) -0.11929(17) 0.0286(5) Uani 1 1 d . . . . .
C2 C 0.32318(13) 0.2312(3) 0.4047(2) 0.0277(6) Uani 1 1 d . . . . .
C5 C 0.22070(12) 0.1735(3) 0.2050(2) 0.0271(6) Uani 1 1 d . . . . .
C11 C 0.41694(13) 0.1907(4) 0.3717(2) 0.0366(7) Uani 1 1 d . . . . .
C12 C 0.43618(13) 0.0646(4) 0.3455(2) 0.0394(8) Uani 1 1 d . . . . .
```

C13 C 0.49308(19) 0.0284(7) 0.3708(3) 0.0692(15) Uani 1 1 d D . . . .
H13 H 0.5082 -0.0549 0.3559 0.104 Uiso 1 1 calc R U . A 1
C14A C 0.5260(8) 0.146(3) 0.426(2) 0.133(17) Uani 0.5 1 d D . P B 1
H14A H 0.5648 0.1260 0.4482 0.199 Uiso 0.5 1 calc R U P B 1
C15A C 0.5128(10) 0.275(2) 0.451(2) 0.089(8) Uani 0.5 1 d D . P B 1
H15A H 0.5383 0.3429 0.4815 0.134 Uiso 0.5 1 calc R U P B 1
C14B C 0.5365(5) 0.1026(15) 0.4235(11) 0.051(3) Uani 0.5 1 d D . P B 2
H14B H 0.5748 0.0769 0.4389 0.076 Uiso 0.5 1 calc R U P B 2
C15B C 0.5152(9) 0.2229(19) 0.4510(18) 0.075(7) Uani 0.5 1 d D . P B 2
H15B H 0.5422 0.2773 0.4941 0.112 Uiso 0.5 1 calc R U P B 2
C16 C 0.4569(2) 0.2834(6) 0.4249(3) 0.0697(14) Uani 1 1 d D . . . .
H16 H 0.4417 0.3638 0.4448 0.105 Uiso 1 1 calc R U . B 1
C51 C 0.19668(11) 0.2021(3) 0.0930(2) 0.0253(6) Uani 1 1 d . . . . .
C52 C 0.19583(11) 0.3309(3) 0.04790(19) 0.0251(5) Uani 1 1 d . . . . .
H52 H 0.2068 0.4087 0.0877 0.038 Uiso 1 1 calc R U . . .
C53 C 0.17829(11) 0.3409(3) -0.0578(2) 0.0275(6) Uani 1 1 d . . . . .
H53 H 0.1777 0.4272 -0.0875 0.041 Uiso 1 1 calc R U . . .
C55 C 0.16129(12) 0.1103(3) -0.0746(2) 0.0322(6) Uani 1 1 d . . . . .
H55 H 0.1490 0.0346 -0.1161 0.048 Uiso 1 1 calc R U . . .
C56 C 0.17771(13) 0.0904(3) 0.0298(2) 0.0305(6) Uani 1 1 d . . . . .
H56 H 0.1761 0.0035 0.0576 0.046 Uiso 1 1 calc R U . . .
O21 O 0.0690(4) 0.0851(8) 0.2142(7) 0.192(4) Uani 1 1 d D U . . .
N22 N 0.0261(5) 0.076(2) 0.2720(9) 0.328(10) Uani 1 1 d D U . . .
C23 C 0.0508(7) 0.053(3) 0.3844(10) 0.351(10) Uani 1 1 d D U . . .
H231 H 0.0917 0.0584 0.3982 0.421 Uiso 1 1 calc R U . . .
H232 H 0.0367 0.1221 0.4233 0.421 Uiso 1 1 calc R U . . .
H233 H 0.0397 -0.0359 0.4034 0.421 Uiso 1 1 calc R U . . .

loop_
 _atom_site_aniso_label
 _atom_site_aniso_U_11
 _atom_site_aniso_U_22
 _atom_site_aniso_U_33
 _atom_site_aniso_U_23
 _atom_site_aniso_U_13
 _atom_site_aniso_U_12
Cl12 0.0606(5) 0.0401(5) 0.0331(4) 0.0023(3) 0.0112(4) 0.0117(3)
S2 0.0426(4) 0.0324(4) 0.0153(4) 0.0033(2) 0.0017(3) -0.0011(3)
O5 0.0635(15) 0.0297(11) 0.0237(10) 0.0050(8) 0.0101(10) -0.0032(9)
N1 0.0384(13) 0.0328(12) 0.0168(11) 0.0014(9) 0.0033(9) -0.0012(10)
N3 0.0442(14) 0.0372(13) 0.0135(10) 0.0011(9) 0.0045(9) 0.0085(11)
N4 0.0429(13) 0.0278(12) 0.0137(10) 0.0015(8) 0.0031(9) 0.0047(10)
N54 0.0301(11) 0.0360(13) 0.0189(10) -0.0016(9) 0.0040(9) 0.0041(9)
C2 0.0427(15) 0.0181(11) 0.0201(12) 0.0002(9) 0.0029(11) -0.0007(10)
C5 0.0358(14) 0.0273(13) 0.0196(12) 0.0006(10) 0.0090(10) 0.0046(11)
C11 0.0328(15) 0.0541(19) 0.0208(13) 0.0101(12) 0.0018(11) -0.0133(13)
C12 0.0323(15) 0.065(2) 0.0222(13) 0.0112(13) 0.0084(11) 0.0044(14)
C13 0.0344(19) 0.143(5) 0.0325(18) 0.032(2) 0.0123(16) 0.022(3)
C14A 0.028(7) 0.28(4) 0.095(15) 0.11(2) 0.029(8) 0.063(14)
C15A 0.061(9) 0.129(18) 0.068(8) 0.034(12) -0.007(6) -0.073(12)
C14B 0.010(5) 0.101(7) 0.040(4) 0.029(5) 0.004(4) 0.007(6)
C15B 0.054(9) 0.098(15) 0.050(7) 0.040(9) -0.032(6) -0.063(11)
C16 0.072(3) 0.090(3) 0.039(2) 0.006(2) -0.0039(19) -0.047(3)
C51 0.0295(13) 0.0277(13) 0.0192(12) -0.0002(9) 0.0069(10) 0.0028(10)
C52 0.0297(13) 0.0261(12) 0.0193(12) -0.0005(9) 0.0055(10) 0.0033(10)
C53 0.0299(13) 0.0319(14) 0.0206(12) 0.0024(10) 0.0054(10) 0.0060(11)
C55 0.0350(14) 0.0363(15) 0.0241(13) -0.0049(11) 0.0041(11) -0.0047(12)
C56 0.0391(15) 0.0290(13) 0.0234(13) -0.0006(10) 0.0070(11) -0.0032(11)
O21 0.190(7) 0.134(5) 0.203(8) 0.047(5) -0.054(6) -0.026(5)
N22 0.179(11) 0.58(3) 0.181(12) -0.002(15) -0.053(7) -0.092(13)
C23 0.202(12) 0.59(3) 0.220(13) -0.010(16) -0.030(10) -0.080(14)

_geom_special_details
;
 All esds (except the esd in the dihedral angle between two l.s. planes)
 are estimated using the full covariance matrix. The cell esds are taken
 into account individually in the estimation of esds in distances, angles
 and torsion angles; correlations between esds in cell parameters are only
 used when they are defined by crystal symmetry. An approximate (isotropic)
 treatment of cell esds is used for estimating esds involving l.s. planes.

;

loop_
 _geom_bond_atom_site_label_1
 _geom_bond_atom_site_label_2
 _geom_bond_distance
 _geom_bond_site_symmetry_2
 _geom_bond_publ_flag
Cl12 C12 1.728(4) . ?
S2 C2 1.696(3) . ?
O5 C5 1.220(3) . ?
N1 C2 1.335(4) . ?
N1 C11 1.422(4) . ?
N1 H1 0.89(4) . ?
N3 C2 1.346(4) . ?
N3 N4 1.388(3) . ?
N3 H3 1.03(4) . ?
N4 C5 1.351(4) . ?
N4 H4 0.86(4) . ?
N54 C55 1.339(4) . ?
N54 C53 1.339(4) . ?
C5 C51 1.508(4) . ?
C11 C12 1.388(5) . ?
C11 C16 1.391(5) . ?
C12 C13 1.378(5) . ?
C13 C14B 1.333(14) . ?
C13 C14A 1.49(3) . ?
C13 H13 0.9300 . ?
C14A C15A 1.36(3) . ?
C14A H14A 0.9300 . ?
C15A C16 1.31(2) . ?
C15A H15A 0.9300 . ?
C14B C15B 1.36(3) . ?
C14B H14B 0.9300 . ?
C15B C16 1.49(2) . ?
C15B H15B 0.9300 . ?
C16 H16 0.9300 . ?
C51 C56 1.390(4) . ?
C51 C52 1.392(4) . ?
C52 C53 1.388(4) . ?
C52 H52 0.9300 . ?
C53 H53 0.9300 . ?
C55 C56 1.381(4) . ?
C55 H55 0.9300 . ?
C56 H56 0.9300 . ?
O21 N22 1.430(9) . ?
N22 N22 1.26(2) 2 ?
N22 C23 1.507(9) . ?
C23 H231 0.9600 . ?
C23 H232 0.9600 . ?
C23 H233 0.9600 . ?

loop_
 _geom_angle_atom_site_label_1
 _geom_angle_atom_site_label_2
 _geom_angle_atom_site_label_3
 _geom_angle
 _geom_angle_site_symmetry_1
 _geom_angle_site_symmetry_3
 _geom_angle_publ_flag
C2 N1 C11 123.7(2) . . ?
C2 N1 H1 120(3) . . ?
C11 N1 H1 116(3) . . ?
C2 N3 N4 122.1(2) . . ?
C2 N3 H3 127(2) . . ?
N4 N3 H3 111(2) . . ?
C5 N4 N3 119.4(2) . . ?
C5 N4 H4 126(3) . . ?
N3 N4 H4 115(3) . . ?
C55 N54 C53 117.2(2) . . ?

N1 C2 N3 117.9(2) . . ?
N1 C2 S2 123.7(2) . . ?
N3 C2 S2 118.4(2) . . ?
O5 C5 N4 122.2(2) . . ?
O5 C5 C51 121.9(3) . . ?
N4 C5 C51 115.9(2) . . ?
C12 C11 C16 118.3(4) . . ?
C12 C11 N1 120.5(3) . . ?
C16 C11 N1 121.1(4) . . ?
C13 C12 C11 122.3(4) . . ?
C13 C12 Cl12 118.1(4) . . ?
C11 C12 Cl12 119.6(2) . . ?
C14B C13 C12 127.2(8) . . ?
C12 C13 C14A 108.2(8) . . ?
C12 C13 H13 125.9 . . ?
C14A C13 H13 125.9 . . ?
C15A C14A C13 135.0(16) . . ?
C15A C14A H14A 112.5 . . ?
C13 C14A H14A 112.5 . . ?
C16 C15A C14A 106.0(17) . . ?
C16 C15A H15A 127.0 . . ?
C14A C15A H15A 127.0 . . ?
C13 C14B C15B 108.2(12) . . ?
C13 C14B H14B 125.9 . . ?
C15B C14B H14B 125.9 . . ?
C14B C15B C16 132.1(13) . . ?
C14B C15B H15B 114.0 . . ?
C16 C15B H15B 114.0 . . ?
C15A C16 C11 129.9(13) . . ?
C11 C16 C15B 111.5(8) . . ?
C15A C16 H16 115.1 . . ?
C11 C16 H16 115.1 . . ?
C56 C51 C52 118.1(2) . . ?
C56 C51 C5 117.4(2) . . ?
C52 C51 C5 124.4(2) . . ?
C53 C52 C51 118.6(2) . . ?
C53 C52 H52 120.7 . . ?
C51 C52 H52 120.7 . . ?
N54 C53 C52 123.5(3) . . ?
N54 C53 H53 118.2 . . ?
C52 C53 H53 118.2 . . ?
N54 C55 C56 123.4(3) . . ?
N54 C55 H55 118.3 . . ?
C56 C55 H55 118.3 . . ?
C55 C56 C51 119.1(3) . . ?
C55 C56 H56 120.4 . . ?
C51 C56 H56 120.4 . . ?
N22 N22 C23 126.0(17) 2 . ?
N22 N22 O21 120.9(15) 2 . ?
C23 N22 O21 112.7(11) . . ?
N22 C23 H231 109.5 . . ?
N22 C23 H232 109.5 . . ?
H231 C23 H232 109.5 . . ?
N22 C23 H233 109.5 . . ?
H231 C23 H233 109.5 . . ?
H232 C23 H233 109.5 . . ?

loop_
 _geom_torsion_atom_site_label_1
 _geom_torsion_atom_site_label_2
 _geom_torsion_atom_site_label_3
 _geom_torsion_atom_site_label_4
 _geom_torsion
 _geom_torsion_site_symmetry_1
 _geom_torsion_site_symmetry_2
 _geom_torsion_site_symmetry_3
 _geom_torsion_site_symmetry_4
 _geom_torsion_publ_flag
C2 N3 N4 C5 94.4(3) . . . . ?
C11 N1 C2 N3 -178.8(3) . . . . ?

C11 N1 C2 S2 3.1(4) . . . . ?
N4 N3 C2 N1 5.1(4) . . . . ?
N4 N3 C2 S2 -176.7(2) . . . . ?
N3 N4 C5 O5 -3.6(4) . . . . ?
N3 N4 C5 C51 178.3(2) . . . . ?
C2 N1 C11 C12 107.2(3) . . . . ?
C2 N1 C11 C16 -75.5(4) . . . . ?
C16 C11 C12 C13 0.3(5) . . . . ?
N1 C11 C12 C13 177.7(3) . . . . ?
C16 C11 C12 Cl12 -179.8(3) . . . . ?
N1 C11 C12 Cl12 -2.4(4) . . . . ?
C11 C12 C13 C14B 1.6(9) . . . . ?
Cl12 C12 C13 C14B -178.4(8) . . . . ?
C11 C12 C13 C14A -0.8(11) . . . . ?
Cl12 C12 C13 C14A 179.3(10) . . . . ?
C12 C13 C14A C15A -3(3) . . . . ?
C13 C14A C15A C16 6(4) . . . . ?
C12 C13 C14B C15B 1.0(17) . . . . ?
C13 C14B C15B C16 -6(3) . . . . ?
C14A C15A C16 C11 -7(3) . . . . ?
C12 C11 C16 C15A 4.2(15) . . . . ?
N1 C11 C16 C15A -173.1(14) . . . . ?
C12 C11 C16 C15B -3.9(11) . . . . ?
N1 C11 C16 C15B 178.7(10) . . . . ?
C14B C15B C16 C11 8(3) . . . . ?
O5 C5 C51 C56 -7.3(4) . . . . ?
N4 C5 C51 C56 170.8(3) . . . . ?
O5 C5 C51 C52 177.2(3) . . . . ?
N4 C5 C51 C52 -4.7(4) . . . . ?
C56 C51 C52 C53 -2.5(4) . . . . ?
C5 C51 C52 C53 173.0(2) . . . . ?
C55 N54 C53 C52 2.6(4) . . . . ?
C51 C52 C53 N54 -0.2(4) . . . . ?
C53 N54 C55 C56 -2.2(4) . . . . ?
N54 C55 C56 C51 -0.5(4) . . . . ?
C52 C51 C56 C55 2.8(4) . . . . ?
C5 C51 C56 C55 -173.0(3) . . . . ?

_refine_diff_density_max   0.618
_refine_diff_density_min   -0.843
_refine_diff_density_rms   0.102

_shelx_res_file
;

    shelx.res created by SHELXL-2014/7

```
TITL mm-39a in C2/c
CELL  1.54184  24.0692   9.7448  13.4379   90.000  103.383   90.000
ZERR     8.00   0.0005   0.0002   0.0003    0.000    0.002    0.000
LATT   7
SYMM  - X,   Y, 1/2 - Z
SFAC  C    H    CL   N    O    S
UNIT  112  112   8    40   16   8
MERG   2
FMAP   2
PLAN  -5
ACTA
BOND   $H
CONF
DFIX  1.400 0.01  N22 O21
DFIX  1.500 0.01  N22 C23
DFIX  1.400 0.05  C13 C14A C14A C15A C15A C16
DFIX  1.400 0.05  C13 C14B C14B C15B C15B C16
DELU  0.01  0.01  O21  N22
SIMU  0.01  0.01  N22  C23
L.S.  40
WGHT    0.111200    7.292500
FVAR       0.24752
```

```
MOLE    1
CL12  3    0.387774   -0.052948    0.278960    11.00000    0.06062    0.04012 =
        0.03310    0.00230    0.01117    0.01173
S2    6    0.344757    0.204227    0.532572    11.00000    0.04259    0.03239 =
        0.01532    0.00330    0.00174   -0.00109
O5    5    0.218553    0.059641    0.241565    11.00000    0.06353    0.02968 =
        0.02373    0.00497    0.01008   -0.00320
N1    4    0.358123    0.225923    0.340779    11.00000    0.03837    0.03280 =
        0.01680    0.00137    0.00332   -0.00119
H1    2    0.344538    0.239082    0.274136    11.00000   -1.50000
N3    4    0.268083    0.263548    0.365615    11.00000    0.04418    0.03725 =
        0.01349    0.00112    0.00455    0.00849
H3    2    0.235885    0.266081    0.404531    11.00000   -1.50000
N4    4    0.246214    0.280580    0.261361    11.00000    0.04287    0.02780 =
        0.01375    0.00152    0.00306    0.00472
H4    2    0.251303    0.361020    0.238104    11.00000   -1.50000
N54   4    0.162206    0.232781   -0.119292    11.00000    0.03010    0.03599 =
        0.01892   -0.00157    0.00397    0.00407
C2    1    0.323177    0.231192    0.404736    11.00000    0.04272    0.01809 =
        0.02012    0.00025    0.00290   -0.00072
C5    1    0.220702    0.173493    0.204964    11.00000    0.03578    0.02732 =
        0.01955    0.00064    0.00904    0.00463
C11   1    0.416936    0.190735    0.371667    11.00000    0.03283    0.05411 =
        0.02078    0.01008    0.00176   -0.01331
C12   1    0.436175    0.064563    0.345502    11.00000    0.03230    0.06481 =
        0.02222    0.01116    0.00844    0.00440
C13   1    0.493079    0.028409    0.370797    11.00000    0.03443    0.14289 =
        0.03255    0.03208    0.01233    0.02162
AFIX  43
H13   2    0.508224   -0.054861    0.355936    11.00000   -1.50000
AFIX   0
PART  1
C14A  1    0.526049    0.145556    0.425940    10.50000    0.02829    0.28186 =
        0.09488    0.11146    0.02918    0.06278
AFIX  43
H14A  2    0.564760    0.126031    0.448244    10.50000   -1.50000
AFIX   0
C15A  1    0.512849    0.274662    0.451420    10.50000    0.06142    0.12883 =
        0.06769    0.03391   -0.00660   -0.07296
AFIX  43
H15A  2    0.538268    0.342864    0.481491    10.50000   -1.50000
AFIX   0
PART  0
PART  2
C14B  1    0.536545    0.102641    0.423517    10.50000    0.00989    0.10129 =
        0.04010    0.02869    0.00432    0.00656
AFIX  43
H14B  2    0.574790    0.076905    0.438914    10.50000   -1.50000
AFIX   0
C15B  1    0.515211    0.222920    0.450980    10.50000    0.05439    0.09775 =
        0.05046    0.03974   -0.03205   -0.06274
AFIX  43
H15B  2    0.542204    0.277332    0.494113    10.50000   -1.50000
AFIX   0
PART  0
C16   1    0.456904    0.283377    0.424908    11.00000    0.07179    0.08977 =
        0.03942    0.00636   -0.00388   -0.04665
AFIX  43
H16   2    0.441698    0.363810    0.444785    11.00000   -1.50000
AFIX   0
C51   1    0.196683    0.202108    0.092968    11.00000    0.02954    0.02773 =
        0.01916   -0.00021    0.00686    0.00281
C52   1    0.195829    0.330932    0.047898    11.00000    0.02970    0.02609 =
        0.01935   -0.00045    0.00554    0.00332
AFIX  43
H52   2    0.206777    0.408692    0.087736    11.00000   -1.50000
AFIX   0
C53   1    0.178287    0.340857   -0.057777    11.00000    0.02986    0.03194 =
        0.02059    0.00237    0.00537    0.00605
AFIX  43
```

```
H53   2    0.177665    0.427213   -0.087477    11.00000   -1.50000
AFIX   0
C55   1    0.161293    0.110284   -0.074624    11.00000    0.03498    0.03625 =
        0.02410   -0.00486    0.00405   -0.00470
AFIX  43
H55   2    0.148967    0.034650   -0.116067    11.00000   -1.50000
AFIX   0
C56   1    0.177709    0.090445    0.029815    11.00000    0.03910    0.02904 =
        0.02341   -0.00061    0.00705   -0.00324
AFIX  43
H56   2    0.176095    0.003513    0.057565    11.00000   -1.50000
AFIX   0
MOLE    2
O21   5    0.069044    0.085148    0.214245    11.00000    0.18953    0.13372 =
        0.20302    0.04697   -0.05401   -0.02645
N22   4    0.026143    0.076461    0.271966    11.00000    0.17873    0.57893 =
        0.18106   -0.00190   -0.05281   -0.09230
C23   1    0.050784    0.053144    0.384429    11.00000    0.20189    0.59128 =
        0.21966   -0.01003   -0.03032   -0.07976
AFIX  33
H231  2    0.091684    0.058372    0.398210    11.00000   -1.20000
H232  2    0.036701    0.122133    0.423295    11.00000   -1.20000
H233  2    0.039660   -0.035922    0.403402    11.00000   -1.20000
AFIX   0
HKLF    4

REM  mm-39a in C2/c
REM R1 =  0.0661 for    2901 Fo > 4sig(Fo)  and  0.0688 for all    3143 data
REM    235 parameters refined using     15 restraints

END

WGHT      0.1381      6.7709

REM Highest difference peak  0.618,  deepest hole -0.843,  1-sigma level  0.102
Q1    1   0.5177  0.6229  0.2860  11.00000  0.05    0.62
Q2    1   0.4608  0.5533  0.2206  11.00000  0.05    0.62
Q3    1   0.0000  0.0120  0.2500  10.50000  0.05    0.50
Q4    1   0.4303  0.4588  0.3251  11.00000  0.05    0.46
Q5    1   0.4444  0.4258  0.4317  11.00000  0.05    0.33
;
```
